# Multi-Locus Genome-Wide Association Studies for 14 Main Agronomic Traits in Barley

**DOI:** 10.3389/fpls.2018.01683

**Published:** 2018-11-20

**Authors:** Xin Hu, Jianfang Zuo, Jibin Wang, Lipan Liu, Genlou Sun, Chengdao Li, Xifeng Ren, Dongfa Sun

**Affiliations:** ^1^College of Plant Science and Technology, Huazhong Agricultural University, Wuhan, China; ^2^Guiyang College of Traditional Chinese Medicine, Guiyang, China; ^3^Biology Department, Saint Mary's University, Halifax, NS, Canada; ^4^School of Veterinary and Life Sciences, Murdoch University, Murdoch, WA, Australia; ^5^Hubei Collaborative Innovation Center for Grain Industry, Jingzhou, China

**Keywords:** genome-wide association study, barley, multi-locus model, doubled haploid population, quantitative trait nucleotide, candidate gene

## Abstract

The agronomic traits, including morphological and yield component traits, are important in barley breeding programs. In order to reveal the genetic foundation of agronomic traits of interest, in this study 122 doubled haploid lines from a cross between cultivars “Huaai 11” (six-rowed and dwarf) and “Huadamai 6” (two-rowed) were genotyped by 9680 SNPs and phenotyped 14 agronomic traits in 3 years, and the two datasets were used to conduct multi-locus genome-wide association studies. As a result, 913 quantitative trait nucleotides (QTNs) were identified by five multi-locus GWAS methods to be associated with the above 14 traits and their best linear unbiased predictions. Among these QTNs and their adjacent genes, 39 QTNs (or QTN clusters) were repeatedly detected in various environments and methods, and 10 candidate genes were identified from gene annotation. Nineteen QTNs and two genes (*sdw1/denso* and *Vrs1*) were previously reported, and eight candidate genes need to be further validated. The *Vrs1* gene, controlling the number of rows in the spike, was found to be associated with spikelet number of main spike, spikelet number per plant, grain number per plant, grain number per spike, and 1,000 grain weight in multiple environments and by multi-locus GWAS methods. Therefore, the above results evidenced the feasibility and reliability of genome-wide association studies in doubled haploid population, and the QTNs and their candidate genes detected in this study are useful for marker-assisted selection breeding, gene cloning, and functional identification in barley.

## Introduction

Barley (*Hordeum vulgare* L, 2*n* = 2*x* = 14), one of the first domesticated grains in the Fertile Crescent (Zohary et al., 2012), has been used widely as animal feed, human health foods, and a source of beer. Its yield and quality are the most important breeding objectives in crop breeding programs.

Most agronomic traits, such as plant height- and yield-related traits, are controlled by quantitative trait loci (QTLs) in barley, so it is difficult to obtain their genetic foundation and molecular mechanism. Plant height and its component traits serve as major plant morphological traits affecting barley seed yield. An appropriate plant height is a prerequisite for obtaining the desired yield in barley-breeding programs. To date, more than 30 types of dwarfing or semi-dwarfing genes have been detected, while only a few have been successfully used in barley breeding program, such as *uzu* and *sdw1*/*denso* (Jia et al., 2009; Ren et al., 2010, 2013). Moreover, a large number of QTLs for plant height related traits were reported to be located on all the seven chromosomes (Sameri et al., 2006; Baghizadeh et al., 2007; Wang et al., 2010; Ren et al., 2014). Grain yield is the key trait for the breeder in barley breeding program, therefore, the yield related traits including spike number per plant (SP), grain number per plant (GP), grain weight per plant (GWP), and 1,000 grain weight (TGW), have gained more attentions in the genetic dissection of yield related traits. A huge number of QTLs for yield related traits were detected to be located across all the chromosomes (Li et al., 2005; Sameri et al., 2006; Baghizadeh et al., 2007; Wang et al., 2010, 2016a; Ren et al., 2013).

Traditionally, QTL mapping has been widely applied in the genetic dissection of quantitative traits in barley (Zhuang et al., 1997; Li et al., 2005, 2007; Peng et al., 2011). As the development of DNA sequencing technologies, it is relatively easy to obtain high-density SNP genotypes for association mapping (AM) population, which offers a huge convenience for genomic and genetic research in different species. Therefore, genome-wide association studies (GWAS) present a powerful tool to reconnect the complex quantitative traits with their genes. Due to the development of cheaper, faster and higher-throughput molecular markers, AM has been widely used for mapping QTLs and genes in many crops, such as maize, soybean, rice, barley and wheat (Huang et al., 2010; Yang et al., 2010; Pasam et al., 2012; Hu et al., 2015). In comparison with traditional QTL mapping, AM has three obvious advantages, including shorter construction time, much higher mapping resolution and a greater number of alleles (Zhang et al., 2005; Yu and Buckler, 2006). In barley, AM has been widely applied for complex traits including disease resistance (Massman et al., 2011), drought tolerance (Varshney et al., 2012; Wójcik-Jagła et al., 2018), salinity tolerance (Fan et al., 2016) and especially agronomic traits (Gawenda et al., 2015; Xu et al., 2018).

Beside for natural population, nowadays GWAS have been widely applied to the genetic analysis for complex traits in family-based populations, such as nested association mapping (NAM) and multi-parent advanced generation intercross (MAGIC) populations, and proved to be powerful tool for uncovering the basis of key agronomic traits in maize and barley (Tian et al., 2011; Cook et al., 2012; Maurer et al., 2015, 2016). Moreover, there are also successful cases that combine linkage analysis with GWAS in several bi-parental segregation populations, such as recombinant inbred line (RIL) population (Lu et al., 2010; Reif et al., 2010). For single segregating population, successful but fewer cases were performed using GWAS (Gao et al., 2015; Henning et al., 2016; Liu et al., 2018). Henning et al. (2016) conducted GWAS for downy mildew resistance in a segregating population of “Teamaker” × USDA 21422M in hop (*Humulus lupulus* L.), Gao et al. (2015) determined the location of TTKSK resistance in the 108 doubled haploid (DH) lines using a GWAS method implemented by R package rrBLUP (Endelman, 2011), and Liu et al. (2018) used two strategies (QTL mapping and GWAS) to reveal the genetic bases of fiber quality traits and yield components in 231 RILs. Therefore, it is feasible to use GWAS to dissect the genetic foundations of complex traits in single bi-parental segregating population. A combination of linkage and association methodologies should provide the more accurate and powerful approach for revealing the genetic bases of complex traits (Ott et al., 2011). Association mapping of drought tolerance-related traits was performed in barley to complement a traditional bi-parental QTL mapping study in Wójcik-Jagła et al. (2018).

The objectives of this study were to: (a) use GWAS to further dissect the genetic foundations for main agronomic traits in our previous studies of Ren et al. (2013, 2014) and Wang et al. (2016a), and compare the quantitative trait nucleotide (QTN) results with those in previous studies, (b) evaluate if GWAS is feasible and reliable in the genetic dissection of complex traits in DH population, and (c) mine the candidate genes in the regions of the QTNs. The outcome of this study will provide more precise and complete information for further gene cloning, and marker-assisted selection in barley breeding.

## Materials and methods

### Plant materials and field experiments

One hundred and twenty-two DH lines, derived from a cross between barley cultivar “Huaai 11” (six-rowed and dwarfing) and barley cultivar “Huadamai 6” (two-rowed), was used in this study. The details of the materials and field experiments were described in the previous studies of Ren et al. (2010, 2013, 2014) and Wang et al. (2016a).

### Phenotyping data

Fourteen agronomic traits for the above DH population were measured in 2008–2009, 2009–2010, and 2011–2012, and all the three datasets had been reported by Ren et al. (2013, 2014) and Wang et al. (2016a). These traits included plant height (PH), first internode length (IL1), second internode length (IL2), third internode length (IL3), fourth internode length (IL4), main spike length (MSL), spike number per plant (SP), spikelet number of main spike (SMS), spikelet number per plant (SLP), grain number per plant (GP), grain number per spike (GS), grain weight per plant (GWP), grain weight per spike (GWS), and 1,000 grain weight (TGW). All the details have been described in Ren et al. (2013, 2014).

The best linear unbiased predictions (BLUPs) for each trait of 3 years were calculated using the R package Lme4 (Bates et al., 2014) with the following model: *y* = lmer (Trait ~ (1|Genetype) + (1|Year)). The three single-year phenotypic values (Ren et al., 2013, 2014; Wang et al., 2016a) and their BLUP values were used for GWAS. The results of phenotype statistics of BLUP for each trait were summarized in Table S1.

### Genotyping data

All the above DH lines were genotyped by 10,367 polymorphic SNPs. After excluding low quality SNP markers, 9680 SNPs were used in this study. Base on the recent genome sequence release in barley (Ibsc, 2016; Beier et al., 2017; Mascher et al., 2017), all the SNP markers were aligned to the most reliable genome position (http://webblast.ipk-gatersleben.de/barley_ibsc/). The *Vrs1* locus controlling row number of barley was integrated with SNP markers for GWAS. All the above information has been described in Ren et al. (2016).

### GWAS

Q matrix was calculated by STRUCTURE software (Falush et al., 2003), and the optimal K was inferred in Figure S1. The kinship (K) matrix between the lines was calculated as previously described in Wang et al. (2016b). All the 9680 SNPs for the above 122 DH lines were used to conduct GWAS for the above 14 traits in 3 years and their BLUP values using five multi-locus GWAS methods, including mrMLM (Wang et al., 2016b), FASTmrMLM (Zhang and Tamba, 2018), FASTmrEMMA (Wen et al., 2018), pLARmEB (Zhang et al., 2017) and ISIS EM-BLASSO (Tamba et al., 2017), which were included in the R package mrMLM v3.1 (https://cran.r-project.org/web/packages/mrMLM/index.html). All parameters in GWAS were set at default values. The critical thresholds of significant association for the five methods were set as LOD = 3 (or *P*-value = 2 × 10^−4^; Wang et al., 2016b).

The significant QTNs, repeatedly detected in at least two environments or methods, were viewed as reliable. The associated regions on chromosomes, repeatedly located on same or similar traits in at least 2 years or methods, were viewed as reliable QTN clusters. These QTNs (or clusters) were named as “qtn (qtnc)” + trait name abbreviation + chromosome + detected QTL order on chromosome.

### Phenotypes difference corresponding to QTNs

For each QTN, all the DH lines were firstly divided into two groups based on their QTN genotypes, then *t*-test was used to test the phenotypic difference between the two genotypes.

### Identification of candidate genes

According to the recent genome sequence release of barley (Ibsc, 2016; Beier et al., 2017; Mascher et al., 2017) and the gene annotation information (http://plants.ensembl.org/Hordeum_vulgare/Info/Index and https://www.uniprot.org/uniprot), some genes around reliable QTNs (or clusters) were selected for each trait. By combining gene annotation information, protein domain function in database and previous reports, and expressional information (http://barlex.barleysequence.org), then, candidate genes for each trait were mined.

## Results

### GWAS for 14 agronomic traits

**Figure 1:**
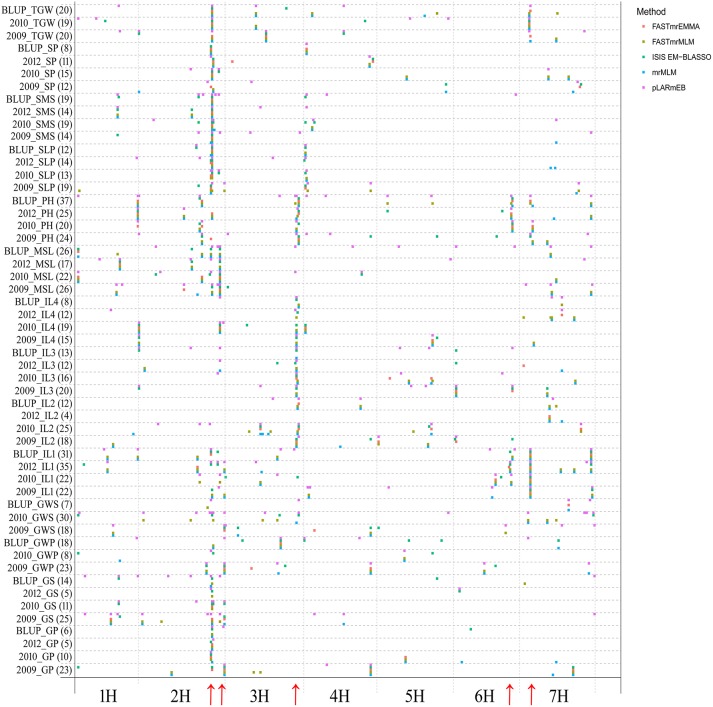
Chromosomal distribution of QTNs identified in this study. The *x*-axis indicates genomic locations by chromosomal order, and the significant QTNs are plotted against genome location. Each row represents one QTN identified by a different method. The red arrows show the QTN hotspots.

**Figure 2:**
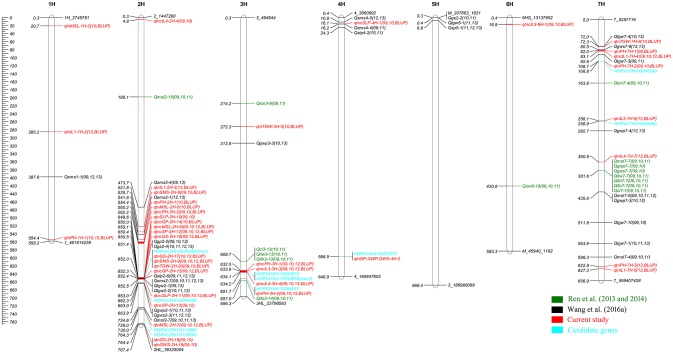
Chromosomes location of reliable QTLs for 14 agronomic traits in both previous studies (Ren et al., 2013, 2014; Wang et al., 2016a) and the current studies. The peak positions of previous QTLs were used for mapping, Genetic distance scale in physic position (Mb) is placed at left margin. Green is for the QTLs detected in Ren et al. (2013, 2014), black is for the QTLs Wang et al. (2016a), red is for the QTNs and QTNs clusters of current study, the region of QTNs clusters was marked with red on the bar, the cyan is for the candidate genes.

Using five multi-locus GWAS methods in the R package mrMLM v3.1, GWAS for 14 agronomic traits were performed. A total of 913 significant QTNs were found to be associated with the 14 agronomic traits in 3 years and their BLUP values (Table S2). The number of significant QTNs varied across various traits, ranging from 4 for 2012_IL4 to 37 for BLUP_PH (Figure 1; Table S2), the chromosomal distribution of all identified QTNs revealed that 2H had the maximum number of significant QTNs, which weren't evenly distributed on the genome, and five QTN hotspots on chromosomes 2H, 3H, 6H, and 7H were observed (Figures 1, 2).

The significant QTNs repeatedly detected by multiple methods were list in Table S3. The reliable QTNs (or clusters) for 14 agronomic traits were summarized in Table 1. Totally, there were 39 reliable QTNs (or clusters) for 14 agronomic traits [8 for PH, 4 for IL1, 1 for IL2, 2 for IL3, 3 for IL4, 4 for MSL, 2 for SP, 2 for SMS, 3 for SLP, 3 for TGW, 2 for GP, 3 for GS, 1 for GWS, and 1 QTN cluster for (GP-GWP-GWS)].

**Table 1:** Reliable QTNs and QTN clusters for 14 agronomic traits using multi-locus GWAS methods.

**QTN (QTN cluster)^a^**	**Trait**	**Marker associated**	**Physic position (bp)**	**LOD score**	***r*^2^ (%)**	**Year**	**Previous QTL**
*qtnPH-1H-1*	PH	*1_463006138*	1H: 554371992	3.39–7.64	0.63–4.07	2010, 2012, BLUP
*qtnPH-2H-1*	PH	*M_1999039_472*	2H: 540094243	5.54–16.21	2.82–4.64	2010, BLUP
*qtncPH-2H-2*	PH	*2_447773331*–*M_1663886_573*	2H: 560195592–564116957	3.51–12.47	2.12–3.79	2009, 2010, BLUP
*qtncPH-3H-1*	PH	*3HL_37004393*–*3_511749149*	3H: 631870705–633068955	3.38–15.96	2.15–7.23	2009, 2010, 2012, BLUP	*Qcl3-13, Qitw3-13, Qith3-13* (Ren et al., 2014)
*qtnPH-3H-2*	PH	*2HS_32409186*	3H: 651696476	4.18–16.72	3.13–9.03	2009, 2010, 2012, BLUP	*Qifo3-14* (Ren et al., 2014)
*qtnPH-7H-1*	PH	*7HS_12212266*	7H: 81959684	3.33–9.13	1.07–2.39	2009, BLUP	*qIN3-7HS, qIN4-7HS* (Sameri et al., 2009)
*qtnPH-7H-2*	PH	*7HS_33062962*	7H: 108670637	3.72–6.60	2.31–6.07	2009, 2010, BLUP	*qIN3-7HS, qIN4-7HS* (Sameri et al., 2009)
*qtnPH-7H-3*	PH	*M_249593_1037*	7H: 622802079	3.38–10.17	0.69–2.68	2012, BLUP
*qtnIL1-1H-2*	IL1	*M_173442_1610*	1H: 285199675	4.31–5.02	2.20–4.69	2012, BLUP
*qtnIL1-2H-3*	IL1	*2_399823529*	2H: 521774247	3.23–5.44	2.26–5.96	2012, BLUP
*qtncIL1-7H-4*	IL1	*7HS_21829337*–*7_95992736*	7H: 81889341–84350472	3.21–14.46	3.53–22.62	2009, 2010, 2012, BLUP	*qIN3-7HS, qIN4-7HS* (Sameri et al., 2009)
*qtnIL1-7H-5*	IL1	*7_575487388*	7H: 627311039	3.11–7.81	1.60–6.27	2012, BLUP
*qtnIL2-7H-6*	IL2	*M_114215_455*	7H: 258071311	3.13–7.27	25.82–55.77	2012, BLUP
*qtncIL3-3H-3*	IL3	*3HL_37004393*–*3_511668322*	3H: 631870705–636535362	3.01–21.75	4.88–21.70	2009, 2010, 2012, BLUP	*Qith3-13* (Ren et al., 2014)
*qtncIL3-6H-1*	IL3	*6_14536026*–*6_18118681*	6H: 16165407–17542081	3.15–6.71	1.89–3.68	2009, 2012, BLUP
*qtncIL4-2H-4*	IL4	*M_1778358_754*–*2_4900503*	2H: 4629895–4950022	3.58–5.09	1.79–6.02	2009, 2010
*qtncIL4-3H-4*	IL4	*3HL_37004393*–*3_511668322*	3H: 631870705–636535362	5.69–14.07	5.73–15.97	2009, 2010, 2012, BLUP	*Qifo3-14* (Ren et al., 2014)
*qtnIL4-7H-7*	IL4	*7HL_11281033*	7H: 360793216	3.00–3.46	6.10–19.57	2012, BLUP	*Qifo7-7* (Ren et al., 2014)
*qtnMSL-1H-3*	MSL	*1_35132055*	1H: 20685614	3.21–6.62	1.49–3.46	2010, BLUP
*qtnMSL-2H-5*	MSL	*2_447773331*	2H: 560195592	3.99–8.37	2.92–6.35	2010, BLUP
*qtncMSL-2H-6*	MSL	*2_522610509*–*2HL_34260490*	2H: 648821931–651436685	3.61–10.16	1.57–4.65	2009, 2010, 2012, BLUP
*qtnMSL-2H-7*	MSL	*2_600749073*	2H: 727985438	3.29–22.92	2.09–18.16	2009, 2010, 2012, BLUP	*Qmsl2-7* (Wang et al., 2016a)
*qtnSMS-2H-8*	SMS	*2_406934594*	2H: 535680815	3.62–12.96	3.14–5.39	2009, 2010, BLUP	*Qsms2-1* (Wang et al., 2016a)
*qtnSMS-2H-9*	SMS	*Vrs1*	2H: 652030802	6.24–82.16	65.07–90.41	2009, 2010, 2012, BLUP	*Qsms2-7* (Wang et al., 2016a)
*qtnSLP-2H-10*	SLP	*2_524762464*	2H: 649558019	4.51–21.01	14.87–55.05	2009, 2010	*Qslp2-6* (Wang et al., 2016a)
*qtncSLP-2H-11*	SLP	*Vrs1*–*2HL_17075593*	2H: 652030802–653982961	3.32–51.41	8.97–80.30	2009, 2010, 2012, BLUP	*Qslp2-6* (Wang et al., 2016a)
*qtncSLP-4H-1*	SLP	*4_16553551*–*M_1605646_794*	4H: 15498372–16761959	3.26–9.76	2.82–4.14	2009, 2010, BLUP	*Qslp4-2* (Wang et al., 2016a)
*qtncSP-2H-12*	SP	*2_522610509*–*Vrs1*	2H: 648821931–652030802	4.02–30.41	7.61–52.32	2009, 2010, 2012, BLUP
*qtncSP-2H-13*	SP	*2_531255437*–*M_124056_833*	2H: 662335248–663628734	3.73–7.32	6.49–11.95	2009, 2012
*qtncGP-2H-14*	GP	*2_524762464*–*M_1589358_1352*	2H: 649558019–650438830	3.46–17.05	19.56–37.44	2010, BLUP	*Qgp2-2* (Wang et al., 2016a)
*qtncGP-2H-15*	GP	*Vrs1*–*2_527241334*	2H: 652030802–652604015	3.42–16.71	3.41–34.59	2009, 2012, BLUP	*Qgp2-2* (Wang et al., 2016a)
*qtncGS-2H-16*	GS	*2_524762464*–*2_527636020*	2H: 649558019–651399477	3.04–17.50	9.77–71.50	2009, 2012, BLUP
*qtnGS-2H-17*	GS	*Vrs1*	2H: 652030802	11.28–48.96	49.99–72.25	2010, 2012, BLUP	*Qgs2-4* (Wang et al., 2016a)
*qtnGS-2H-18*	GS	*2_625783669*	2H: 764361924	3.48–5.87	1.06–10.05	2009, 2010
*qtnGWS-2H-19*	GWS	*2_625783669*	2H: 764361924	3.29–7.37	0.74–6.25	2009, 2010
*qtn(GP-GWP- GWS)-4H-2*	GP, GWP, GWS	*4_497278091*	4H: 596447744	3.04–6.81	3.97–9.17	2009
*qtnTGW-2H-20*	TGW	*Vrs1*	2H: 652030802	4.82–49.42	32.77–56.66	2009, 2010, BLUP	*Qtgw2-1, Qtgw2-2* (Wang et al., 2016a)
*qtnTGW-3H-5*	TGW	*3HS_23539468*	3H: 272283784	3.88–5.98	2.19–4.39	2010, BLUP
*qtnTGW-7H-8*	TGW	*7HS_10887541*	7H: 72344563	6.27–13.16	4.39–11.22	2010, BLUP	*Qtgw7-4* (Wang et al., 2016a)

a *Reliable QTNs and QTN clusters which was detected at least in 2 years environments and multiple GWAS methods; b, physic position of chromosome based on the blast result for the sequence of marker in barley genome database (http://webblast.ipk-gatersleben.de/barley_ibsc/)*.

#### The QTNs for plant height and its components traits

Six reliable QTNs and two reliable QTN clusters, distributed on four chromosomes, were significantly associated with PH (Table 1; Figure 2). Six reliable QTNs for PH, *qtnPH-1H-1* (1H: 554,371,992 bp), *qtnPH-2H-1* (2H: 540,094,243 bp), *qtnPH-3H-2* (3H: 651,696,476 bp), *qtnPH-7H-1* (7H: 81,959,684 bp), *qtnPH-7H-2* (7H:108,670,637 bp), and *qtnPH-7H-3* (7H: 622,802,079 bp), located on chromosome 1H, 2H, 3H, 7H, 7H, and 7H, explained 0.63–4.07, 2.82–4.64, 3.13–9.03, 1.07–2.39, 2.31–6.07, and 0.69–2.68% of total phenotypic variation, respectively. Two reliable QTN clusters for PH, *qtncPH-2H-2* (2H: 560,195,592–564,116,957 bp) and *qtncPH-3H-1* (3H: 631,870,705–633,068,955 bp), were identified in at least 2 years and methods, explained 2.12–3.79 and 2.15–7.23% of total phenotypic variation, respectively (Tables 1, S3).

4, 1, 2, and 3 reliable QTNs (or clusters) were found to be associated with plant height component traits IL1, IL2, IL3, and IL4, respectively. For IL1, *qtncIL1-7H-4*, close to *qtnPH-7H-1* (7H: 81,959,684 bp) for PH, was located on 7H (81,889,341–84,350,472 bp) accounting for a highest phenotypic variation (3.53–22.62%) among the four QTNs and QTN clusters (Table 1). For IL2, *qtnIL2-7H-6* on chromosome 7H: 258,071,311 bp, explained phenotypic variation of 25.82–55.77%. For IL3, *qtncIL3-3H-3* was located at 3H: 631,342,028–636,535,362 bp, close to the region of *qtncPH-3H-1* (3H: 631,870,705–633,068,955 bp) for PH, accounting for 4.88–21.70% of the phenotypic variation. *qtncIL3-6H-1* located on the chromosome 6H (16,165,407–17,542,081 bp), explained phenotypic variation of 1.89–3.68%. For IL4, *qtncIL4-3H-4* (3H: 631,870,705–636,535,362 bp), co-located in the same region of *qtncIL3-3H-3* for IL3 and *qtncPH-3H-1* for PH mentioned above, explained 5.73–15.97% of phenotypic variation. Therefore, the region on chromosome 3H: 631,342,028–636,535,362 bp is a more credible QTN cluster for PH and the components traits, regulating the PH through controlling IL3 and IL4 (Table 1). Another QTN cluster for IL4, *qtncIL4-2H-4* on 2H (4,629,895–4,950,022 bp) explained less (1.79–6.02%) phenotypic variation, while QTN *qtnIL4-7H-7* (7H: 360793216 bp) showed a high explanation (6.10–19.57%) for IL4 (Tables 1, S3; Figure 2).

#### The QTNs for spike and yield related traits

Main spike length (MSL): Three reliable QTNs and one QTN cluster were detected for MSL. Among which, *qtnMSL-2H-7* located on chromosome 2H: 727,985,438 bp, was repeatedly detected not only in three environments and BLUP values but also by multiple methods to be significantly associated with MSL, and explained phenotypic variation about 2.09–18.16% (LOD score: 3.29–22.92; Tables 1, S3; Figure 2).

Spikelet number of main spike (SMS): *qtnSMS-2H-9* referred to *Vrs1* (the morphological markers for row number of barley), located at 2H (position: 652,030,802 bp), was identified in all the situations and methods to be significantly associated with SMS (Tables 1, S3), accounting for the largest phenotypic variation (65.07–90.41%). As already known, *Vrs1*, a gene controlling row number of barley, was validated controlling row number in the DH population derived from a cross between the six-rowed barley cultivar “Huaai 11” and the two-rowed barley cultivar “Huadamai 6.” Thus, *Vrs1* should control the spike related traits, such as SMS and SLP. Moreover, *qtnSMS-2H-8* (2H: 535,680,815 bp) with minor effect was associated with SMS (Table 1).

Spikelet number per plant (SLP): One reliable QTN *qtnSLP-2H-10* (2H: 649,558,019 bp) and one reliable QTN clusters *qtncSLP-2H-11* (2H: 652,030,802–653,982,961 bp) close to *Vrs1* (2H: 652,030,802 bp) were identified in multiple environments and by multiple methods to be significantly associated with SLP, explaining high proportions of total phenotypic variation, 14.87–55.05 and 8.97–80.30%, respectively. The reliable QTN cluster *qtncSLP-4H-1* with a minor effect (2.82–4.14%), mapped on the region 15,498,372–16,168,735 bp of chromosome 4H, was detected in 2 years and the BLUP (Tables 1, S3; Figure 2).

Spike number per plant (SP): Two reliable QTN clusters for SLP were detected. *qtncSP-2H-12*, located on 2H: 648,821,931–652,030,802 bp overlapping with *Vrs1*, was detected in three environments and BLUP value and by multiple methods to be associated with SP, accounting for 7.61–52.32% of the phenotypic variation. *qtncSP-2H-13*, located on chromosome 2H (Position: 662,335,248–663,628,734 bp), was detected in two environments and by multiple methods to be associated with SP, explaining 6.49–11.95% of the phenotypic variation (Tables 1, S3; Figure 2).

Grain number per plant (GP) and grain number per spike (GS): The QTN cluster *qtncGP-2H-14* with a high proportion of total phenotypic variation (19.56–37.44%), located at 2H: 649,558,019–650,438,830 bp, was detected in 2010 and BLUP value to be associated with GP. The *qtncGP-2H-15* with a high explanation (3.41–34.59%), close to *Vrs1* (2H: 652,030,802–652,604,015 bp), was detected in three environments and by multiple methods to be associated with GP (Table 1). One small-effect QTN on 4H (Position: 596,447,744 bp), was detected in 2009 to be significantly associated with GP. Meanwhile, this QTN was also detected for GWP and GWS (Table 1). One QTN cluster and two QTNs were detected for GS in at least two environments and by multiple methods. The *qtncGS-2H-16* (2H: 649,558,019–651,399,477 bp), close to *Vrs1*, showed a high proportion of phenotypic variation (9.77–71.5%) for GS. The *qtnGS-2H-17* referred to *Vrs1* (2H: 652,030,802 bp) was significantly associated with GS, explaining a high percentage (49.99–72.25%) of the phenotypic variation. The reliable QTN *qtnGS-2H-18*, located at chromosome 2H (Position: 764,361,924 bp), was significantly associated with GS in 2009 and 2010 and multiple methods, accounting for 1.06–10.05% of the phenotypic variation (Tables 1, S3; Figure 2).

Grain weight per plant (GWP) and grain weight per spike (GWS): One QTN on 4H (Position: 596,447,744 bp) was found to be associated with GP, GWP and GWS with 3.97–9.17% proportions for the phenotypic variation. The QTN *qtnGWS-2H-19*, derived from the same associated SNP *2_625783669* (2H: 764,361,924 bp) as GS, was detected for GWS in 2009 and 2010 and by multiple GWAS methods with 0.74–6.25% proportion of phenotypic variation (Tables 1, S3; Figure 2).

1,000 grain weight (TGW): three reliable QTNs were detected for TGW. The *qtnTGW-2H-20*, located at *Vrs1* (2H: 652,030,802 bp), was significantly associated with TGW in multiple environments and GWAS methods, explaining high percentage (32.77–56.66%) of the phenotypic variation. The *qtnTGW-3H-5* (3H: 272,283,784 bp) and *qtnTGW-7H-8* (7H: 72,344,563 bp) were significantly associated with TGW, explaining 2.19–4.39 and 4.39–11.22% of the phenotypic variation, respectively (Tables 1, S3; Figure 2).

### Phenotypic difference corresponding to QTNs

**Figure 3:**
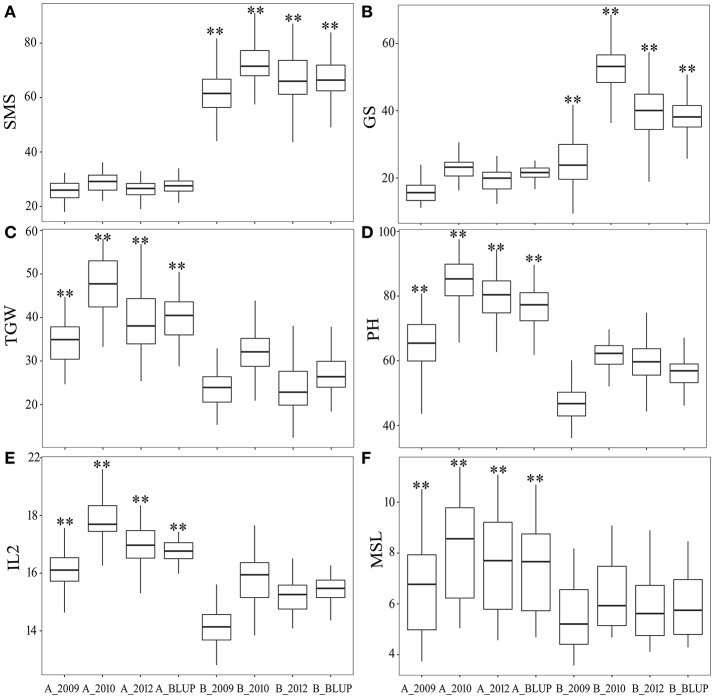
The difference of phenotypes between two kinds of genotypes for each of the six QTNs. **(A)**: SMS at *qtnSMS-2H-9*, **(B)**: GS at *qtnGS-2H-17*, **(C)**: TGW at *qtnTGW-2H-20*, **(D)**: PH at *qtnPH-7H-2*, **(E)**: IL2 at *qtnIL2-7H-6*, **(F)**: MSL at *qtnMSL-2H-7*. **Significant difference at *P* < 0.01.

According to the QTN genotypes, all the DH lines were divided into two different groups to test whether the significant difference of corresponding phenotypes of the QTN genotypes exist using *t*-test. Here, six reliable QTNs were used to underlying the phenotypes difference as an example. The details were showed in Figure 3.

Among six QTNs, three yield related QTNs (*qtnSMS-2H-9* for SMS, *qtnGS-2H-17* for GS, and *qtnTGW-2H-20* for TGW) had the significant differences of phenotypic averages between their two genotypes in all four environments (Figures 3A–C), and three PH related QTNs (*qtnPH-7H-2, qtnIL2-7H-6*, and *qtnMSL-2H-7*) had the significant differences in all four environments (Figures 3D–F), indicating their reliability. It was worth noting that the *qtnMSL-2H-7* and *qtnSMS-2H-9* was detected in all the four environments, the *qtnPH-7H-2, qtnTGW-2H-20*, and *qtnGS-2H-17* was only detected in three environments, and the *qtnIL2-7H-6* was detected only in 2012 and the BLUP (Table 1).

### Identification of candidate genes around reliable QTNs (or clusters)

According to the recently released genome sequence of barley (Ibsc, 2016; Beier et al., 2017; Mascher et al., 2017) and the gene annotation information, ten candidate genes for the traits of interest were detected around the reliable QTNs and QTN clusters (Table 2). The chromosomal distribution of candidate genes was showed in Figure 2. Among which, two genes correspond to the previously reported genes, such as *sdw1/denso* and *Vrs1*, while eight candidate genes were new and their functions were derived from the annotated information, which need to be further validated (Table 2). Among eight new genes, *HORVU3Hr1G090970, HORVU7Hr1G040290, HORVU7Hr1G058360*, and *HORVU3Hr1G096010* are related to plant height and its component traits, encoding SAM-dependent_Mtases, Alpha-mannosidase, DHHC-cysteine-rich domain S-acyltransferase and Homeobox-like, respectively. *HORVU2Hr1G113880, HORVU2Hr1G094080, HORVU2Hr1G126690*, and *HORVU4Hr1G075070* are associated with spike and yield related traits, encoding AP2-like ethylene-responsive transcription factor, BTB/POZ domain protein, Acyl-CoA N-acetyltransferase and Patatin, respectively (Table 2).

**Table 2:** Candidate genes around the reliable QTNs and QTN clusters.

**QTN (QTN cluster)^a^**	**Trait**	**Marker**	**Physic position (bp)^b^**	**candidate gene^c^**	**Physic position (bp)**	**Annotation^d^**
*qtncPH-3H-1*	PH	*3HL_37004393*–*3_511749149*	3H: 631870705–633068955	*HORVU3Hr1G090980, HORVU3Hr1G090970*	3H: 634,077,598–634,081,600, 3H: 634,071,757–634,080,040	*sdw1/denso*, GA20-oxidases; SAM-dependent_Mtases, like *PvSAMS*
*qtncIL3-3H-3*	IL3	*3HL_37004393*–*3_511668322*	3H: 631870705–636535362	*HORVU3Hr1G090980, HORVU3Hr1G090970*	3H: 634,077,598–634,081,600, 3H: 634,071,757–634,080,040	*sdw1/denso*, GA20-oxidases; SAM-dependent_Mtases, like *PvSAMS*
*qtncIL4-3H-4*	IL4	*3HL_37004393*–*3_511668322*	3H: 631870705–636535362	*HORVU3Hr1G090980, HORVU3Hr1G090970*	3H: 634,077,598–634,081,600, 3H: 634,071,757–634,080,040	*sdw1/denso*, GA20-oxidases; SAM-dependent_Mtases, like *PvSAMS*
*qtnPH-7H-2*	PH	*7HS_33062962*	7H: 108670637	*HORVU7Hr1G040290*	7H:108834015–108839990	*AMS1p*, Alpha-mannosidase
*qtnIL2-7H-6*	IL2	*M_114215_455*	7H: 258071311	*HORVU7Hr1G058360*	7H: 258,860,422–258,866,854	DHHC-cysteine-rich domain S-acyltransferase
*qtnPH-3H-2*	PH	*2HS_32409186*	3H: 651696476	*HORVU3Hr1G096010*	3H: 651,659,644–651,663,119	Homeobox-like, SANT/Myb
*qtnSMS-2H-9*	SMS	*Vrs1*	2H: 652030802	*HORVU2Hr1G092290*	2H: 652,031,058–652,032,990	*Vrs1*, Homeobox
*qtncSLP-2H-11*	SLP	*Vrs1*–*2HL_17075593*	2H: 652030802–653982961	*HORVU2Hr1G092290*	2H: 652,031,058–652,032,990	*Vrs1*, Homeobox
*qtncGP-2H-15*	GP	*Vrs1*–*2_527241334*	2H: 652030802–652604015	*HORVU2Hr1G092290*	2H: 652,031,058–652,032,990	*Vrs1*, Homeobox
*qtnGS-2H-17*	GS	*Vrs1*	2H: 652030802	*HORVU2Hr1G092290*	2H: 652,031,058–652,032,990	*Vrs1*, Homeobox
*qtnTGW-2H-20*	TGW	*Vrs1*	2H: 652030802	*HORVU2Hr1G092290*	2H: 652,031,058–652,032,990	*Vrs1*, Homeobox
*qtnMSL-2H-7*	MSL	*2_600749073*	2H: 727985438	*HORVU2Hr1G113880*	2H:730027508–730030208	AP2-like ethylene-responsive transcription factor
*qtncSP-2H-13*	SP	*2_531255437*–*M_124056_833*	2H: 662335248–663628734	*HORVU2Hr1G094080*	2H: 662,298,334–662,300,891	BTB/POZ
*qtnGS-2H-18*	GS	*2_625783669*	2H: 764361924	*HORVU2Hr1G126690*	2H: 764,279,329–764,290,102	Acyl-CoA N-acetyltransferase
*qtnGWS-2H-19*	GWS	*2_625783669*	2H: 764361924	*HORVU2Hr1G126690*	2H: 764,279,329–764,290,102	Acyl-CoA N-acetyltransferase
*qtn(GP-GWP- GWS)-4H-2*	GP, GWP, GWS	*4_497278091*	4H: 596447744	*HORVU4Hr1G075070*	4H: 596,446,043–596,448,382	Patatin

a *Reliable QTNs and QTN clusters which were detected at least in 2 years environments and multiple GWAS methods*;

b *Physic position of chromosome based on the blast result for the sequence of marker in barley genome database (http://webblast.ipk-gatersleben.de/barley_ibsc/)*;

c *Candidate gene was acquired from http://plants.ensembl.org/Hordeum_vulgare/Info/Index*;

d *Annotation information was from the database http://plants.ensembl.org/Hordeum_vulgare/Info/Index and https://www.uniprot.org/uniprot*.

## Discussion

### Previously reported and novel QTNs detected with multi-locus GWAS analysis

The comparison between the reliable QTNs (or clusters) for the main agronomic traits and the reliable QTLs in previous studies (Ren et al., 2010, 2013, 2014; Wang et al., 2016a) were conducted (Table 1; Figure 2). According to the physic positions of associated markers, the reliable QTNs (or clusters) in this study were integrated to the physic map with the reliable QTLs using MapChart 2.32 (Voorrips, 2002; Figure 2). Among 39 reliable QTNs (QTN clusters) detected by GWAS, 19 were located on the same regions of QTLs in previous studies (Sameri et al., 2009; Ren et al., 2014; Wang et al., 2016a), while 20 reliable QTNs (QTN clusters) including some minor effect QTNs were novel (Table 1). Totally, 8 of 18 QTNs (QTN clusters) associated with plant height related traits, were same as those in previous studies (Sameri et al., 2009; Ren et al., 2014), and 10 were new. For spike and yield related traits, 11 of 21 QTNs (QTN clusters) were the same as the QTLs in Wang et al. (2016a), the others were new in the current study (Table 1).

Among 8 previously reported QTNs (QTN clusters) for PH related traits, QTN clusters *qtncPH-3H-1* (3H: 631,870,705–633,068,955 bp), *qtncIL3-3H-3* (3H: 631,870,705–636,535,362 bp), and *qtncIL4-3H-4* (3H: 631,870,705–636,535,362 bp), located on the hotspot of 3H (Figure 2), were significantly associated with PH, IL3, and IL4, respectively (Table 1). Close to the region of the QTNs clusters, *Qcl3-13* for CL (the length from the ground to the collar equal PH minus MSL), *Qitw3-13* for IL2, *Qith3-13* for IL3, and *Qifo3-14* for IL4 were detected close to the region of SSR markers *Bmag13* (Position: 608,671,381 bp) and *Bmag877* (Position: 657,045,459 bp) on 3H, respectively (Ren et al., 2014). Moreover, *qIN6-3HL* was detected to be significantly associated with IN6 (sixth internode) between *Bmag13* (3H: 608,671,381 bp) and *e06m30.8.3* in Sameri et al. (2009). Compared to the physical positions of the QTLs with those of QTNs, the QTN clusters for PH, IL3, and IL4 should correspond to the QTL *Qcl3-13* for CL, *Qith3-13* for IL3, and *Qifo3-14* for IL4, respectively (Ren et al., 2014; Table 1).

The *qtnPH-7H-1* (7H: 81,959,684 bp), *qtnPH-7H-2* (7H: 108,670,637 bp), and *qtncIL1-7H-4* (7H: 81,889,341–84,350,472 bp) were detected in same region of chromosome 7HS to be significantly associated with PH and IL1, respectively. These three QTNs are likely the same to the two QTLs *qIN3-7Hs* and *qIN4-7Hs* identified between markers *HVCMA* (7H: 75,227,158 bp) and *ABG701* (7H: 90,406,550 bp) in Sameri et al. (2009) (Table 1). No consistent QTLs were detected in the region of these QTNs by Ren et al. (2014). It seems that GWAS can detect more minor QTNs for interesting traits than traditional QTL analysis.

Among 11 previously reported QTNs (or clusters) for spike and yield related traits, it is worth noting that most were located close to the region of *Vrs1* gene. Four QTNs and three QTN clusters, such as *qtnSMS-2H-9* (2H: 652,030,802 bp) for SMS, *qtnGS-2H-17* (2H: 652,030,802 bp) for GS, *qtnTGW-2H-20* (2H: 652,030,802 bp) for TGW, *qtnSLP-2H-10* (2H: 649,558,019 bp) and *qtncSLP-2H-11* (2H: 652,030,802–653,982,961 bp) for SLP, and *qtncGP-2H-14* (2H: 649,558,019–650,438,830 bp) and *qtncGP-2H-15* (2H: 652,030,802–652,604,015 bp) for GP were detected at *Vrs1* (2H: 652,030,802 bp) and the nearby region of 2H with highly phenotypic variation (Table 1; Figure 2), which was consistent with the QTLs detected in Wang et al. (2016a), including *Qsms2-7* (2H: 652,507,869 bp) for SMS, *Qgs2-4* (2H: 651,436,685 bp) for GS, *Qtgw2-1* (2H: 652,507,869 bp) and *Qtgw2-2* (2H: 652,508,158 bp) for TGW, *Qslp2-6* (2H: 651,436,685 bp) for SLP, and *Qgp2-2* (2H: 651,436,685 bp) for GP (Table 1; Figure 2). Moreover, QTNs (or clusters) *qtnMSL-2H-7* (2H: 727,985,438 bp), *qtnSMS-2H-8* (2H: 535,680,815 bp), *qtncSLP-4H-1* (4H: 15,498,372–16,761,959 bp), and *qtnTGW-7H-8* (7H: 72,344,563 bp) were consistent with the QTLs *Qmsl2-7* (2H: 724,577,184 bp), *Qsms2-1* (2H: 541,758,123 bp), *Qslp4-2* (4H: 24,332,575 bp), and *Qtgw7-4* (7H: 71,957,427 bp) of Wang et al. (2016a) (Table 1; Figure 2).

For 20 novel reliable QTNs (QTN clusters) detected by multi-locus GWAS, most had minor effects (Table 1). It is worth noting that *qtnIL2-7H-6* was a novel QTN associated with IL2 accounting for a higher proportion of phenotypic variation (22.85–55.77%). And the candidate gene *HORVU7Hr1G058360* (7H: 258,860,422–258,866,854 bp), close to *qtnIL2-7H-6* (7H: 258,071,311 bp), may involve in regulating IL2. For other minor effect QTNs (QTN clusters), three reliable candidate genes (*HORVU2Hr1G094080, HORVU2Hr1G126690*, and *HORVU4Hr1G075070*) were identified to be close to the region of *qtncSP-2H-13, qtnGS-2H-18* (or *qtnGWS-2H-19*), and *qtn(GP-GWP-GWS)-4H-2*, respectively (Table 2). Therefore, the novel QTNs (QTN clusters) by multi-locus GWAS were reliable, even with minor effect. In other words, multi-locus GWAS can detect more minor effect QTNs than traditional QTL analysis.

The comparison between QTLs in previous studies (Sameri et al., 2009; Ren et al., 2013, 2014; Wang et al., 2016a) and in current study indicated that the consistent results should be much more reliable, which is valuable for further gene cloning and molecular marker assistant selection for breeding. Meanwhile, it illustrated that GWAS is feasible and reliable to detect significant associations for complex quantitative traits in DH population. Moreover, some new QTNs with minor effects were detected, suggesting that GWAS should be a good complementary to traditional QTL mapping. The combination of linkage and association analysis should provide the more accurate and powerful approach for reveling the genetic base of complex quantitative traits (Ott et al., 2011).

### Two previous reported genes reveal the reliability of multi-locus GWAS

*HORVU3Hr1G090980* (3H: 634,077,598–634,081,600 bp) was identified on the region (3H: 631,870,705–636,535,362 bp) of three QTN clusters for plant height related traits (*qtncPH-3H-1* for PH, *qtncIL3-3H-3* for IL3, and *qtncIL4-3H-4* for IL4; Table 2; Figure 2). This gene *HORVU3Hr1G090980* correspondeds to the previously reported gene *sdw1/denso*, which was a semi-dwarf gene encoding a gibberellin 20-oxidase enzyme in barley (Jia et al., 2009; Xu et al., 2017). It was clarified that GA 20-oxidases encoded by *sdw1/denso* affected the plant height involving in the later steps of GA biosynthesis (Spielmeyer et al., 2004; Jia et al., 2009; Xu et al., 2017; Table 2). Therefore, the association between three QTN clusters and three height related traits (PH, IL3, and IL4) might be the effect of the gene *sdw1/denso*. *Vrs1* (2H: 652,030,802 bp) was detected to be associated with SMS (*qtnSMS-2H-9*), GS (*qtnGS-2H-17*), and TGW (*qtnTGW-2H-20*) with high proportion of phenotypic variation in multiple environments and by multi-locus GWAS methods in current study (Tables 1, 2). Around the gene *Vrs1*, moreover, five QTNs (or clusters), including *qtnSLP-2H-10* (2H: 649,558,019 bp) and *qtncSLP-2H-11* (2H: 652,030,802–653,982,961 bp) for SLP, *qtncGP-2H-14* (2H: 649,558,019–650,438,830 bp), and *qtncGP-2H-15* (2H: 652,030,802–652,604,015 bp) for GP, and *qtncSP-2H-12* (2H: 648,821,931–652,030,802 bp) for SP, were detected in multiple situations and by multi-locus GWAS methods with high proportion of phenotypic variation (Table 1; Figure 2). *Vrs1*, controlling row number of barley, encodes homeobox and profoundly affects barley spike morphology (Komatsuda et al., 2007). The identification of the reliable QTNs (or clusters) around two previously reported genes using multi-locus GWAS further revealed the reliability of multi-locus GWAS in bi-parental segregation population.

### Novel candidate genes reveal the possible molecular basis of plant height- and yield-related traits

Gene *HORVU3Hr1G090970* (3H: 634,071,757–634,080,040 bp), encoded a S-adenosylmethionine (SAM)-dependent-MTases, which was identified on the region (3H: 631,870,705–636,535,362 bp) of three QTN clusters for plant height related traits (*qtncPH-3H-1* for PH, *qtncIL3-3H-3* for IL3, and *qtncIL4-3H-4* for IL4; Table 2; Figure 2). It was reported that SAM biosynthetic pathways affects lignin biosynthesis in switchgrass (*Panicum virgatum* L.; Bai et al., 2018; Table 2). Cystathionine c-synthase (CGS) is the first committed enzyme for the biosynthesis of Met that can be metabolized to SAM, CGS-RNAi transgenic switchgrass lines showed much shorter plant height and internode length (Bai et al., 2018). Therefore, *HORVU3Hr1G090970* might be a new semi-dwarf gene in barley.

*HORVU7Hr1G008720*, was identified at 7H:108,834,015–108,839,990 bp, which was 0.15Mb from the QTN *qtnPH-7H-2* for PH, encoding Alpha-mannosidase like *AMS1p* (Table 2; Figure 2). Alpha-mannosidase is the component of cell wall, involving in cell wall biosynthesis or modification, which participated in the cell growth of internodes with pectinesterase and alpha-xylosidase in plant (Wu and Cao, 2008). Moreover, Alpha-mannosidase is the member of cytoplasm-to-vacuole targeting (Cvt) pathway with *AuTophaGy8* (*ATG8*) gene, and soybean transgenic lines over-expressed *GmATG8c* showed higher plant height than the wild type (Xia et al., 2012). Therefore, *HORVU7Hr1G008720* should be a reliable candidate gene, which affected PH by regulating the cell growth as *AMS1p* does.

*HORVU7Hr1G058360* at 7H: 258,860,422-258,866,854 bp was close to *qtnIL2-7H-6* (7H: 258,071,311 bp) for IL2, encoding a S-acyltransferase with DHHC-cysteine-rich domain (Table 2; Figure 2). DHHC-cysteine-rich domain S-acyltransferase proteins are involved in plant development and stress responses in *Arabidopsis* (Li et al., 2016). *AtPAT10* is an S-acyl transferase, which affects the vascular development through controlling the cell division and expansion in *Arabidopsis*. *AtPAT10* mutants are semi-dwarfed, and the reduction of plant height is due to the reduced length of the internodes, which appears to be the result of reduction in both cell number and cell size in these tissues (Qi et al., 2013). Therefore, *HORVU7Hr1G058360* is a reliable candidate gene regulating the IL2 as *AtPAT10* dose.

*HORVU3Hr1G096010* (3H: 651,659,644–651,663,119 bp) encoding homeobox-like protein with SANT/MYB domain, was close to the QTN *qtnPH-3H-2* (3H: 651,696,476 bp) for PH (Table 2; Figure 2). Homeobox gene was reported to be involved in the regulation of morphological development in plants, homeobox gene *OSH15* affects the architecture of internodes resulting in d6 dwarf plants (Sato et al., 1999). Moreover, the *RAD* gene in *Arabidopsis*, encoding small plant-specific single SANT/MYB domain protein, affects the growth and development of *Arabidopsis*. Overexpression of the *RAD* gene can repress *Arabidopsis* growth, resulting in dwarfing and delaying flowering (Baxter et al., 2007; Zhang et al., 2011). Thus, *HORVU3Hr1G096010* is a reliable candidate gene regulating plant height as the function of homeobox gene or *RAD* gene.

For main spike length (MSL), *HORVU2Hr1G113880* corresponding to *Cly1* gene, was identified at 2H:730,027,508–730,030,208 bp, which was 3 Mb from the QTN *qtnMSL-2H-7* (2H: 727,985,438 bp) for MSL (Table 2; Figure 2). *Cly1* encodes for an AP2-protein that inhibits development of flower (Nair et al., 2010; Terzi et al., 2017). *HvAP2* regulates the length of a critical developmental window required for the elongation of the inflorescence internodes in barley (Houston et al., 2013). Therefore, the association between *qtnMSL-2H-3* and MSL might be the effect of *Cly1* gene.

For spike number per plant (SP), *HORVU2Hr1G094080* (2H: 662,298,334–662,300,891 bp), encoding a protein with BTB/POZ domain (Broad complex, Tramtrack, Bric à brac, Pox virus and Zinc finger), was detected about 40 Kb from the QTN cluster *qtncSP-2H-13* (2H: 662,335,248–663,628,734 bp; Table 2; Figure 2). *HvCul4* gene encodes a BLADE-ON-PETIOLE-like (BOP-Like) protein containing BTB/POZ domain, which shares high similarity with *Arabidopsis BOP1* and *BOP2* (Tavakol et al., 2015; Jost et al., 2016). It was reported that *HvCul4* controlled the tiller and leaf pattern in barley (Tavakol et al., 2015; Jost et al., 2016), and *Arabidopsis BOP1* and *BOP2* acted at boundary regions to regulate axillary development and leaf morphogenesis (Ha et al., 2004). In addition, according to the barley expression database from Barlex (http://barlex.barleysequence.org), *HORVU2Hr1G094080.1* showed highest level of expression in developing inflorescences. Therefore, candidate gene *HORVU2Hr1G094080* performed the similar function as *HvCul4, BOP1*, and *BOP2* to control the spike number per plant (SP).

For grain number per spike (GS) and grain weight per spike (GWS), marker *2_625783669* (2H: 764,361,924 bp) was detected significantly associated with these 2 traits. *HORVU2Hr1G126690* (2H: 764,279,329–764,290,102 bp), encoding a protein with N-acetyltransferase domain, was detected about 83 Kb from the marker *2_625783669* (Table 2; Figure 2). *OsSNAT1* encodes N-acetyltransferase1, it was reported that overexpression of T2 homozygous *OsSNAT1* in rice increased panicle number and seed weight per plant, while decreased spikelet numbers per panicle under paddy field conditions (Lee and Back, 2017). Moreover, the expression of *HORVU2Hr1G126690.4* is much higher in developing inflorescences than in other tissues according to the barley expression database from Barlex (http://barlex.barleysequence.org). Thus, the candidate gene *HORVU2Hr1G126690* may affect the GS and GWS through the similar function of *OsSNAT1*.

Marker *4_497278091* (4H: 596,447,744 bp) was significant associated with GP, GWP, and GWS. *HORVU4Hr1G075070* (4H: 596,446,043–596,448,382 bp), encoding Patatin, was detected at *4_497278091* (Table 2). Overexpression of a patatin-like protein in *Camelina sativa* (Li et al., 2015) or in *Arabidopsis* (Li et al., 2013) reduced growth and overall seed production, but increased seed oil content. Therefore, *HORVU4Hr1G075070* is a reliable candidate gene which might affect GP, GWP and GWS as the function of patatin-like gene.

Among the above ten candidate genes, two were previously reported, such as *sdw1/denso* and *Vrs1*, eight were new, which were derived from the annotated information. Based on the annotations of these candidate genes, homologous genes or proteins with same function or function domain were reported to be regulated the corresponding traits in barley, *Arabidopsis* and rice. The reliable QTNs and QTN clusters for these traits may be the effect of the candidate genes with similar function as the homologous genes or proteins does. The functions of eight reliable candidate genes need to be further validated. In summary, it is feasible and reliable to use multi-locus GWAS in bi-parental segregation populations.

### The new multi-locus GWAS for bi-parental segregation population

Traditionally, segregation populations were used for QTL analysis, and GWAS are commonly used in natural populations. Nowadays, as the development of high-throughput SNP markers and high-throughput phenotypes, GWAS have been widely applied to the genetic analysis for complex traits in family-based populations (such as NAM and MAGIC populations) and proved to be powerful tool for uncovering the basis of key agronomic traits in maize and barley (Tian et al., 2011; Cook et al., 2012; Maurer et al., 2015, 2016). However, for single segregating population, successful but fewer cases were performed using GWAS (Gao et al., 2015; Henning et al., 2016; Liu et al., 2018). It indicated that GWAS for segregating population are feasible. However, high false positive rate is an obvious problem in the traditional single-locus GWAS using general linear models (GLMs) and mixed linear models (MLMs) (Zhang et al., 2010; Pace et al., 2015). And the *P* threshold (*P* = 0.05/*n, n* is the number of SNPs) leads to missing many significant QTNs, particularly small-effect QTNs (Wang et al., 2016b). Some multi-locus GWAS methodologies, such as mrMLM (Wang et al., 2016b), FASTmrMLM (Zhang and Tamba, 2018), FASTmrEMMA (Wen et al., 2018), ISIS EM-BLASSO (Tamba et al., 2017), pLARmEB (Zhang et al., 2017), and pKWmEB (Ren et al., 2018) have been developed to remedy the shortcomings mentioned above. These multi-locus GWAS methods have been used to analyze the published data, indicated that these methods constituted effective approaches with high detection power and less stringent criteria (Wang et al., 2016b; Tamba et al., 2017; Zhang et al., 2017; Wen et al., 2018). Totally, five multi-locus GWAS methods were used in our study, which improved the detection power and accuracy of QTNs for interesting traits. Moreover, the QTNs (QTN clusters), which were repeatedly detected in multiple environments and GWAS methods, were selected as reliable QTNs (QTN clusters). This greatly improved the accuracy of the association results and reduced its false positive, and more small-effect QTNs were detected within a certain rate of false positive. In addition, the *t*-test results of the phenotypic difference corresponding to QTNs, demonstrated that GWAS have the more stringent threshold of significance than *t*-test, and the advantages of accuracy and false positive controlling. In our study, 39 reliable QTNs and QTN clusters were detected, among which 10 reliable candidate gene were identified. Meanwhile, several new reliable QTNs with small-effect were also detected, which were different from the previous reports (Table 1). There was a limitation to identify candidate genes for all the reliable QTNs and QTN clusters, especially the small-effect ones, based on the imperfect annotation database of barley. However, these results indicated that multi-locus GWAS methods are feasible and reliable for DH population, and good complementary to traditional QTL mapping for the detection of new reliable QTNs even with small-effect. which will provide more useful information for future works.

## Conclusions

Available online at: In this study, five multi-locus GWAS methods were performed for 14 main agronomic traits in 122 doubled haploid (DH) lines. Thirty-nine reliable QTNs and/or QTN clusters were repeatedly detected in multiple environments and methods 10 candidate genes for the interest traits were detected, 19 QTNs and two genes (*sdw1/denso* and *Vrs1*) were previously reported, and eight candidate genes need to be further validated. The results validated the feasibility and reliability of GWAS in DH population and the good complementary to traditional QTL analysis. All the results will facilitate elucidating genetic basis of agronomic traits and improving marker-assisted selection breeding in barley.

## Author contributions

DS and XR conceived and designed the experiments. XH, XR, JW, and LL conducted the experiments and phenotyping measurements. XH and JZ performed the analysis. XH and JZ wrote the paper. GS, XR, and DS modified the manuscript. CL produced the Huaai 11 and Huadamai 6 DH population. All the authors read and approved the final version of this manuscript.

### Conflict of interest statement

The authors declare that the research was conducted in the absence of any commercial or financial relationships that could be construed as a potential conflict of interest.
