# Supplementary material for: Multi-Locus Genome-Wide Association Studies for 14 Main Agronomic Traits in Barley
Source: Front Plant Sci. 2018 Nov 20;9:1683. doi: 10.3389/fpls.2018.01683 (PMC6257129; doi:10.3389/fpls.2018.01683)
Supplement: Supplementary file 1 [file Table_1.DOCX]

Supplementary Material

**Multi-Locus Genome-Wide Association Study for 14 Main Agronomic Traits in Barley**

Xin Hu ^1,2†^, Jianfang Zuo ^1†^, Jibin Wang ^1^, Lipan Liu ^1^, Genlou Sun ^3^, Chengdao Li ^4, 5^, Xifeng Ren ^1*^ and Dongfa Sun ^1, 5*^

^1^ College of Plant Science and Technology, Huazhong Agricultural University, Wuhan, 430070, China

^2^ Guiyang College of Traditional Chinese Medicine, Huaxi District, 550025, Guiyang

^3^ Biology Department, Saint Mary’s University, 923 Robie Street, Halifax, NS, B3H3C3, Canada

^4^ School of Veterinary and Life Sciences, Murdoch University, Murdoch, Western Australia 6150, Australia

^5^ Hubei Collaborative Innovation Center for Grain Industry, Jingzhou, 434025, Hubei, China

**Correspondence:** Dongfa Sun: sundongfa1@mail.hzau.edu.cn;

Xifeng Ren: [renxifeng@mail.hzau.edu.cn](mailto:renxifeng@mail.hzau.edu.cn)

# Supplementary Figures and Tables

## Supplementary Figure


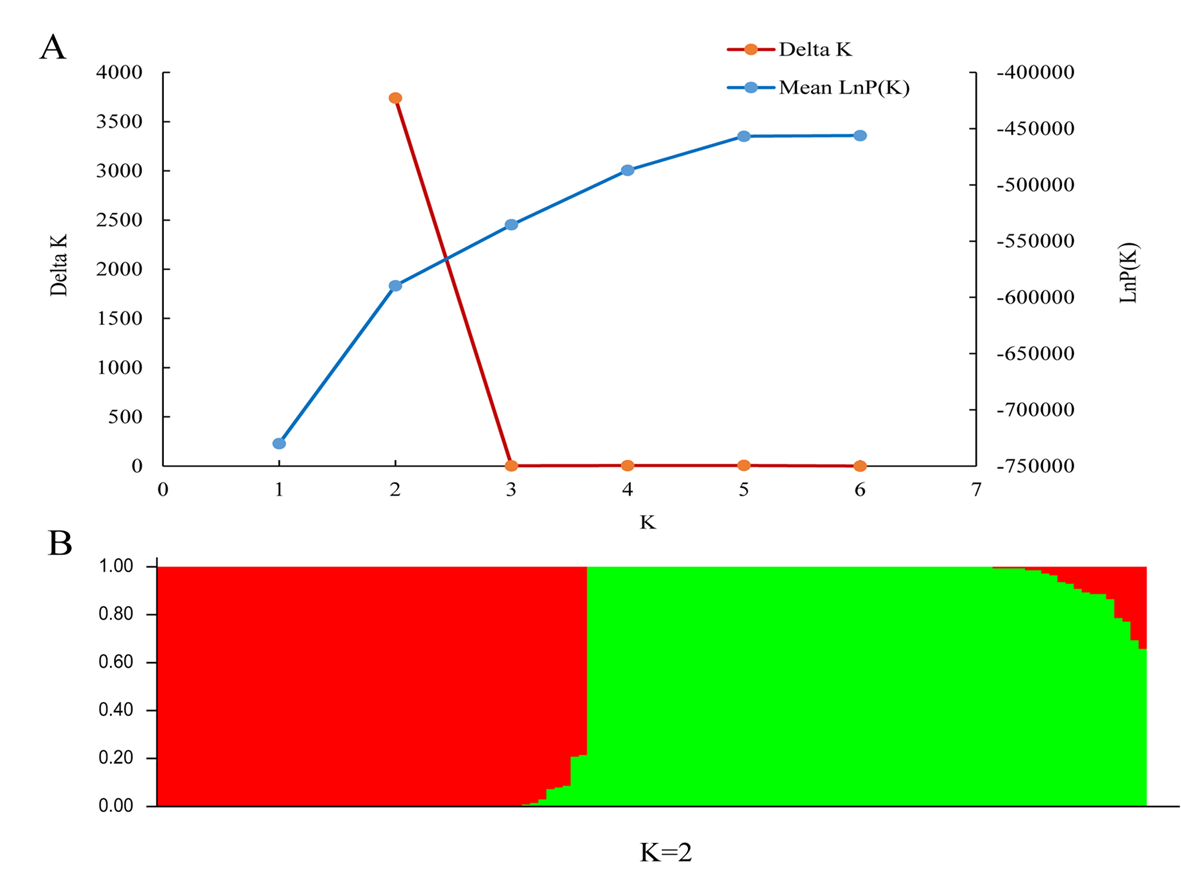


**Figure S1. Population structure estimates based on 9680 SNPs distributed across 7 chromosomes.** (A) Plot of lnP(D), (B) ΔK calculated for K = 2–9. (B) Population structure estimates (K = 2), the areas of the two colors (green and red) illustrate the proportion of each subgroup.

## Supplementary Tables

**Table S1. The Statistics of the BLUP for 14 agronomic traits of 122 DH lines and their parents.**

| Traits | Huadamai6  Mean | Huaai11  mean | DH lines | | | | | |
| --- | --- | --- | --- | --- | --- | --- | --- | --- |
|  |  |  | max | M in | Mean | Skewness | Kurtosis | CV(%) |
| PH | 91.88 | 51.14 | 94.62 | 44.34 | 67.68±12.05 | 0.04 | -1.23 | 17.80 |
| IL1 | 32.30 | 18.34 | 31.58 | 15.06 | 23.39±3.05 | -0.04 | -0.14 | 13.04 |
| IL2 | 15.69 | 12.39 | 21.40 | 10.73 | 14.52±1.73 | 0.43 | 1.07 | 11.91 |
| IL3 | 12.27 | 8.17 | 15.63 | 6.71 | 10.42±1.92 | 0.00 | -0.77 | 18.43 |
| IL4 | 9.73 | 6.24 | 14.09 | 4.79 | 8.2±1.78 | 0.37 | -0.19 | 21.71 |
| MSL | 10.24 | 4.94 | 10.69 | 4.00 | 6.68±1.7 | 0.46 | -0.80 | 25.45 |
| SMS | 35.18 | 56.89 | 88.03 | 21.31 | 55.61±19.1 | -0.63 | -1.12 | 34.35 |
| SLP | 460.76 | 477.98 | 770.25 | 253.89 | 512.79±133.76 | -0.27 | -1.17 | 26.08 |
| SP | 11.95 | 9.47 | 10.69 | 4.00 | 6.68±1.7 | 0.46 | -0.80 | 25.45 |
| GP | 344.37 | 313.77 | 662.11 | 223.41 | 332.91±59.4 | 1.45 | 6.62 | 17.84 |
| GS | 28.71 | 33.43 | 50.74 | 16.69 | 33.34±8.84 | -0.28 | -1.08 | 26.51 |
| GWP | 12.91 | 8.21 | 16.16 | 6.11 | 9.85±1.88 | 0.35 | 0.11 | 19.09 |
| GWS | 1.08 | 1.04 | 1.73 | 1.00 | 1.07±0.07 | 6.53 | 54.55 | 6.54 |
| TGW | 39.78 | 24.77 | 50.49 | 18.40 | 30.54±7.5 | 0.82 | -0.18 | 24.56 |

**Table S2. The significant associations between SNP markers and 14 agronomic traits detected in four environments and five multi-locus GWAS methods.**

| Trait ID ^a^ | Method | Markers | Physic position (bp) | QTN effect | LOD score | r^2^ (%) |
| --- | --- | --- | --- | --- | --- | --- |
| 2009_GP | ISIS EM-BLASSO | 1H_33742078 | 1H: 23002985 | -12.77 | 3.60 | 2.42 |
| 2009_GP | mrMLM | M_124041_539 | 2H: 296731074 | -20.41 | 3.61 | 5.21 |
| 2009_GP | FASTmrMLM | M_124041_539 | 2H: 296731074 | -17.56 | 4.12 | 4.08 |
| 2009_GP | FASTmrEMMA | 2_527241334 | 2H: 652604015 | -32.75 | 3.42 | 3.41 |
| 2009_GP | ISIS EM-BLASSO | 2_527241334 | 2H: 652604015 | -16.38 | 3.84 | 3.61 |
| 2009_GP | FASTmrMLM | 2_624008395 | 2H: 762295561 | -28.39 | 8.63 | 12.05 |
| 2009_GP | pLARmEB | 2_624008395 | 2H: 762295561 | -28.04 | 6.83 | 11.76 |
| 2009_GP | mrMLM | 2_625783669 | 2H: 764361924 | -28.54 | 6.94 | 11.91 |
| 2009_GP | FASTmrEMMA | 2_625783669 | 2H: 764361924 | -56.31 | 7.24 | 10.55 |
| 2009_GP | ISIS EM-BLASSO | 2_625783669 | 2H: 764361924 | -27.92 | 8.23 | 12.07 |
| 2009_GP | FASTmrMLM | 2HL_38899537 | 3H: 255072620 | 23.14 | 4.23 | 7.61 |
| 2009_GP | FASTmrMLM | 3HL_12333356 | 3H: 311188855 | -28.44 | 5.83 | 12.45 |
| 2009_GP | pLARmEB | M_2571394_329 | 4H: 205055735 | -14.59 | 3.00 | 2.57 |
| 2009_GP | mrMLM | 4_497278091 | 4H: 596447744 | 23.46 | 4.76 | 7.77 |
| 2009_GP | FASTmrMLM | 4_497278091 | 4H: 596447744 | 17.18 | 3.59 | 4.41 |
| 2009_GP | FASTmrEMMA | 4_497278091 | 4H: 596447744 | 37.72 | 4.25 | 4.70 |
| 2009_GP | pLARmEB | 4_497278091 | 4H: 596447744 | 22.12 | 5.02 | 7.32 |
| 2009_GP | ISIS EM-BLASSO | 4_497278091 | 4H: 596447744 | 19.26 | 4.98 | 5.55 |
| 2009_GP | mrMLM | 7_162243905 | 7H: 289468666 | -34.25 | 3.31 | 16.09 |
| 2009_GP | mrMLM | 7HL_29446583 | 7H: 462916216 | 42.23 | 4.05 | 25.57 |
| 2009_GP | FASTmrMLM | 7HL_29446583 | 7H: 462916216 | 36.81 | 4.80 | 20.59 |
| 2009_GP | FASTmrEMMA | 7HL_29446583 | 7H: 462916216 | 73.01 | 4.23 | 18.14 |
| 2009_GP | ISIS EM-BLASSO | 7HL_29446583 | 7H: 462916216 | 37.80 | 5.06 | 21.71 |
| 2009_GS | pLARmEB | 1_414284041 | 1H: 84055042 | -1.72 | 5.63 | 4.82 |
| 2009_GS | mrMLM | 1H_18337255 | 1H: 314487734 | 3.14 | 9.87 | 16.02 |
| 2009_GS | FASTmrMLM | 1H_18337255 | 1H: 314487734 | 3.00 | 10.45 | 14.67 |
| 2009_GS | FASTmrEMMA | 1H_18337255 | 1H: 314487734 | 3.97 | 4.57 | 5.80 |
| 2009_GS | pLARmEB | 1H_18337255 | 1H: 314487734 | 1.91 | 4.37 | 5.94 |
| 2009_GS | pLARmEB | M_192877_732 | 1H: 370824123 | 2.00 | 4.02 | 6.64 |
| 2009_GS | ISIS EM-BLASSO | 1H_67020570 | 1H: 398577778 | 2.59 | 6.16 | 10.91 |
| 2009_GS | mrMLM | 2_27682271 | 2H: 34487656 | 2.91 | 7.95 | 13.99 |
| 2009_GS | FASTmrMLM | 2_27682271 | 2H: 34487656 | 2.58 | 7.77 | 11.00 |
| 2009_GS | pLARmEB | 2_33141529 | 2H: 36724705 | 1.18 | 3.29 | 2.27 |
| 2009_GS | FASTmrMLM | M_155197_321 | 2H: 205793259 | -1.53 | 4.24 | 3.97 |
| 2009_GS | pLARmEB | 2_614228896 | 2H: 610878049 | -1.71 | 6.16 | 4.73 |
| 2009_GS | pLARmEB | 2_522710996 | 2H: 647661551 | -3.76 | 9.86 | 17.43 |
| 2009_GS | mrMLM | 2_527636020 | 2H: 651399477 | -4.51 | 17.44 | 26.71 |
| 2009_GS | FASTmrMLM | 2_527636020 | 2H: 651399477 | -4.75 | 17.50 | 29.64 |
| 2009_GS | FASTmrEMMA | 2_527636020 | 2H: 651399477 | -7.03 | 4.85 | 15.72 |
| 2009_GS | pLARmEB | 2_600474867 | 2H: 727925822 | 1.45 | 5.02 | 3.25 |
| 2009_GS | FASTmrMLM | 2_598715827 | 2H: 729178615 | 1.53 | 3.86 | 3.79 |
| 2009_GS | mrMLM | 2_625783669 | 2H: 764361924 | -2.17 | 3.48 | 7.80 |
| 2009_GS | FASTmrEMMA | 2_625783669 | 2H: 764361924 | -3.70 | 3.97 | 4.86 |
| 2009_GS | ISIS EM-BLASSO | 2_625783669 | 2H: 764361924 | -2.46 | 5.87 | 10.05 |
| 2009_GS | pLARmEB | M_1573088_3106 | 4H: 99459706 | -1.32 | 4.65 | 2.97 |
| 2009_GS | mrMLM | 4HL_45271612 | 4H: 357481348 | -2.13 | 4.73 | 7.69 |
| 2009_GS | pLARmEB | 4HL_45271612 | 4H: 357481348 | -1.54 | 5.44 | 4.04 |
| 2009_GS | pLARmEB | M_72984_1488 | 7H: 656211646 | 1.40 | 4.99 | 2.95 |
| 2009_GWP | mrMLM | 2_476515788 | 2H: 609930273 | 0.62 | 5.82 | 7.22 |
| 2009_GWP | FASTmrMLM | 2_476515788 | 2H: 609930273 | 0.53 | 5.25 | 5.30 |
| 2009_GWP | pLARmEB | 2_476515788 | 2H: 609930273 | 0.50 | 5.41 | 4.55 |
| 2009_GWP | ISIS EM-BLASSO | 2_476515788 | 2H: 609930273 | 0.47 | 5.25 | 4.02 |
| 2009_GWP | pLARmEB | 2_624008395 | 2H: 762295561 | -0.44 | 5.86 | 4.10 |
| 2009_GWP | mrMLM | 2HL_38325004 | 2H: 767379851 | -0.57 | 5.08 | 6.97 |
| 2009_GWP | FASTmrMLM | 2HL_38325004 | 2H: 767379851 | -0.45 | 4.14 | 4.30 |
| 2009_GWP | FASTmrEMMA | 2HL_38325004 | 2H: 767379851 | -0.86 | 3.74 | 3.40 |
| 2009_GWP | ISIS EM-BLASSO | 2HL_38325004 | 2H: 767379851 | -0.45 | 5.29 | 4.39 |
| 2009_GWP | FASTmrEMMA | 3HS_25446223 | 3H: 234821450 | -0.93 | 4.03 | 4.02 |
| 2009_GWP | pLARmEB | 3_369808840 | 3H: 462478623 | -0.42 | 4.51 | 3.85 |
| 2009_GWP | mrMLM | 3_542225534 | 3H: 476142806 | -0.55 | 5.05 | 6.64 |
| 2009_GWP | ISIS EM-BLASSO | 3_428711315 | 3H: 538784438 | 0.46 | 5.14 | 4.86 |
| 2009_GWP | mrMLM | 4_497278091 | 4H: 596447744 | 0.66 | 6.81 | 9.17 |
| 2009_GWP | FASTmrMLM | 4_497278091 | 4H: 596447744 | 0.54 | 5.49 | 6.18 |
| 2009_GWP | FASTmrEMMA | 4_497278091 | 4H: 596447744 | 0.95 | 4.03 | 4.26 |
| 2009_GWP | pLARmEB | 4_497278091 | 4H: 596447744 | 0.43 | 4.36 | 3.97 |
| 2009_GWP | mrMLM | 6HS_27276614 | 6H: 260948523 | -0.55 | 4.40 | 6.63 |
| 2009_GWP | FASTmrMLM | 6HS_27276614 | 6H: 260948523 | -0.45 | 4.23 | 4.41 |
| 2009_GWP | pLARmEB | 6HS_27276614 | 6H: 260948523 | -0.47 | 6.19 | 4.93 |
| 2009_GWP | ISIS EM-BLASSO | 6HL_32414003 | 6H: 364118739 | -0.55 | 7.46 | 6.71 |
| 2009_GWP | mrMLM | 7_549668049 | 7H: 608344982 | 0.47 | 3.57 | 4.62 |
| 2009_GWP | pLARmEB | 7_549668049 | 7H: 608344982 | 0.37 | 3.96 | 2.95 |
| 2009_GWS | mrMLM | 1_179136142 | 1H: 332254267 | 0.04 | 3.67 | 5.69 |
| 2009_GWS | FASTmrMLM | 1_179136142 | 1H: 332254267 | 0.04 | 5.47 | 3.93 |
| 2009_GWS | pLARmEB | 1_179136142 | 1H: 332254267 | 0.02 | 3.29 | 1.76 |
| 2009_GWS | FASTmrEMMA | 2_625783669 | 2H: 764361924 | -0.07 | 3.29 | 3.41 |
| 2009_GWS | pLARmEB | 2_625783669 | 2H: 764361924 | -0.04 | 5.28 | 4.95 |
| 2009_GWS | ISIS EM-BLASSO | 2_625783669 | 2H: 764361924 | -0.05 | 7.37 | 6.28 |
| 2009_GWS | pLARmEB | 3HS_2307376 | 3H: 763814 | -0.03 | 3.98 | 2.51 |
| 2009_GWS | ISIS EM-BLASSO | 3_93281834 | 3H: 112963308 | -0.02 | 3.08 | 1.61 |
| 2009_GWS | mrMLM | 3_104318354 | 3H: 118607226 | -0.05 | 4.15 | 7.98 |
| 2009_GWS | FASTmrEMMA | M_1573088_3106 | 4H: 99459706 | -0.08 | 3.60 | 3.99 |
| 2009_GWS | mrMLM | 4_497278091 | 4H: 596447744 | 0.05 | 5.14 | 8.83 |
| 2009_GWS | FASTmrMLM | 4_497278091 | 4H: 596447744 | 0.05 | 3.04 | 7.31 |
| 2009_GWS | ISIS EM-BLASSO | 4_497278091 | 4H: 596447744 | 0.04 | 3.61 | 5.59 |
| 2009_GWS | ISIS EM-BLASSO | 5HS_7374618 | 5H: 2568671 | 0.04 | 3.33 | 3.86 |
| 2009_GWS | FASTmrMLM | 7HL_2485950 | 6H: 458002332 | -0.02 | 3.67 | 1.90 |
| 2009_GWS | pLARmEB | 7HL_2485950 | 6H: 458002332 | -0.03 | 3.53 | 1.98 |
| 2009_GWS | pLARmEB | 7HL_30194632 | 7H: 366828881 | 0.04 | 5.82 | 4.90 |
| 2009_GWS | pLARmEB | 7HL_28046755 | 7H: 650527321 | 0.03 | 3.00 | 2.11 |
| 2009_IL1 | ISIS EM-BLASSO | 2_531247880 | 2H: 652500446 | 0.90 | 6.34 | 5.79 |
| 2009_IL1 | mrMLM | 2_534864652 | 2H: 659635806 | 1.06 | 7.00 | 8.71 |
| 2009_IL1 | FASTmrMLM | 2_534864652 | 2H: 659635806 | 0.91 | 6.86 | 6.41 |
| 2009_IL1 | pLARmEB | 2HL_19270925 | 2H: 712293659 | 0.75 | 4.92 | 4.82 |
| 2009_IL1 | mrMLM | 2HL_38325004 | 2H: 767379851 | -0.85 | 5.06 | 6.49 |
| 2009_IL1 | FASTmrMLM | 2HL_38325004 | 2H: 767379851 | -0.69 | 5.42 | 4.31 |
| 2009_IL1 | pLARmEB | M_114601_946 | 4H: 33779555 | -0.95 | 5.06 | 6.96 |
| 2009_IL1 | mrMLM | 4_59047254 | 4H: 49843189 | -0.74 | 3.61 | 5.05 |
| 2009_IL1 | FASTmrMLM | 4_59047254 | 4H: 49843189 | -0.42 | 3.05 | 1.65 |
| 2009_IL1 | pLARmEB | 4_137655999 | 4H: 58447848 | -1.21 | 7.87 | 13.65 |
| 2009_IL1 | pLARmEB | 5_66389622 | 5H: 96365591 | -0.52 | 3.31 | 2.07 |
| 2009_IL1 | mrMLM | 6HL_37474519 | 6H: 330735066 | -1.30 | 3.18 | 15.74 |
| 2009_IL1 | pLARmEB | 6HL_37474519 | 6H: 330735066 | -1.08 | 8.97 | 10.89 |
| 2009_IL1 | pLARmEB | 7HS_30257307 | 7H: 83769522 | 0.62 | 3.21 | 3.53 |
| 2009_IL1 | FASTmrMLM | 7_95992736 | 7H: 84350472 | 0.87 | 4.56 | 7.14 |
| 2009_IL1 | FASTmrEMMA | 7_95992736 | 7H: 84350472 | 2.08 | 3.56 | 8.43 |
| 2009_IL1 | ISIS EM-BLASSO | 7_95992736 | 7H: 84350472 | 0.79 | 3.78 | 5.82 |
| 2009_IL1 | mrMLM | 7HS_33683527 | 7H: 87379560 | 0.96 | 4.18 | 8.59 |
| 2009_IL1 | mrMLM | M_5654_1031 | 7H: 636383191 | 1.05 | 7.80 | 8.92 |
| 2009_IL1 | FASTmrMLM | M_5654_1031 | 7H: 636383191 | 0.91 | 7.08 | 6.70 |
| 2009_IL1 | pLARmEB | M_5654_1031 | 7H: 636383191 | 0.84 | 6.75 | 5.72 |
| 2009_IL1 | ISIS EM-BLASSO | M_5654_1031 | 7H: 636383191 | 0.77 | 5.37 | 4.76 |
| 2009_IL2 | mrMLM | 1_218835638 | 1H: 335266470 | -0.75 | 5.12 | 9.32 |
| 2009_IL2 | FASTmrMLM | 1_218835638 | 1H: 335266470 | -0.52 | 5.84 | 4.44 |
| 2009_IL2 | mrMLM | 3_511668322 | 3H: 636535362 | -0.63 | 6.07 | 5.49 |
| 2009_IL2 | FASTmrMLM | 3_511668322 | 3H: 636535362 | -0.54 | 5.91 | 4.07 |
| 2009_IL2 | FASTmrEMMA | 3_511668322 | 3H: 636535362 | -1.14 | 6.04 | 4.40 |
| 2009_IL2 | ISIS EM-BLASSO | 3_511668322 | 3H: 636535362 | -0.58 | 6.96 | 4.57 |
| 2009_IL2 | pLARmEB | 3HL_4899915 | 3H: 653353465 | -0.55 | 5.48 | 3.56 |
| 2009_IL2 | mrMLM | 4_345615059 | 4H: 326697662 | -0.34 | 3.33 | 2.07 |
| 2009_IL2 | ISIS EM-BLASSO | 4_497278091 | 4H: 596447744 | 0.28 | 3.62 | 1.36 |
| 2009_IL2 | FASTmrMLM | 5_7535464 | 5H: 7896309 | -0.29 | 3.28 | 1.34 |
| 2009_IL2 | FASTmrEMMA | 5_7535464 | 5H: 7896309 | -0.62 | 3.22 | 1.42 |
| 2009_IL2 | pLARmEB | 5_7535464 | 5H: 7896309 | -0.41 | 4.50 | 2.71 |
| 2009_IL2 | mrMLM | 1H_36913991 | 5H: 448670006 | 0.58 | 5.21 | 5.91 |
| 2009_IL2 | FASTmrMLM | 1H_36913991 | 5H: 448670006 | 0.40 | 4.73 | 2.84 |
| 2009_IL2 | ISIS EM-BLASSO | 6_8174922 | 6H: 7699770 | 0.24 | 3.01 | 0.99 |
| 2009_IL2 | FASTmrEMMA | M_95671_278 | 6H: 11483810 | 0.56 | 3.20 | 1.23 |
| 2009_IL2 | pLARmEB | M_95671_278 | 6H: 11483810 | 0.28 | 3.08 | 1.42 |
| 2009_IL2 | ISIS EM-BLASSO | M_203837_1773 | 6H: 515553605 | -0.28 | 3.40 | 1.33 |
| 2009_IL3 | pLARmEB | 2_1447297 | 2H: 304016 | 0.18 | 3.27 | 0.96 |
| 2009_IL3 | ISIS EM-BLASSO | M_1778358_754 | 2H: 4629895 | 0.23 | 3.26 | 1.64 |
| 2009_IL3 | pLARmEB | 3_275423042 | 3H: 311114825 | -0.24 | 4.58 | 1.72 |
| 2009_IL3 | ISIS EM-BLASSO | 3_508744417 | 3H: 631342028 | -0.45 | 3.05 | 4.65 |
| 2009_IL3 | mrMLM | 3HL_37004393 | 3H: 631870705 | -0.78 | 12.83 | 13.67 |
| 2009_IL3 | FASTmrMLM | 3HL_37004393 | 3H: 631870705 | -0.76 | 12.94 | 12.87 |
| 2009_IL3 | pLARmEB | 3HL_37004393 | 3H: 631870705 | -0.79 | 18.15 | 13.85 |
| 2009_IL3 | pLARmEB | 5HL_29111279 | 5H: 296237059 | -0.37 | 5.60 | 2.68 |
| 2009_IL3 | pLARmEB | 3HL_9150538 | 5H: 426436714 | 0.38 | 7.03 | 3.84 |
| 2009_IL3 | mrMLM | 6_14536026 | 6H: 16165407 | 0.33 | 3.27 | 3.27 |
| 2009_IL3 | FASTmrMLM | 6_14536026 | 6H: 16165407 | 0.25 | 4.13 | 1.89 |
| 2009_IL3 | FASTmrEMMA | 6HS_18979469 | 6H: 16292787 | 0.65 | 4.78 | 2.66 |
| 2009_IL3 | pLARmEB | 6HS_18979469 | 6H: 16292787 | 0.35 | 6.71 | 3.68 |
| 2009_IL3 | ISIS EM-BLASSO | 6_18118681 | 6H: 17542081 | 0.34 | 5.72 | 3.34 |
| 2009_IL3 | FASTmrEMMA | 6_480485322 | 6H: 518364045 | -0.50 | 3.58 | 1.71 |
| 2009_IL3 | pLARmEB | 6_480485322 | 6H: 518364045 | -0.22 | 3.43 | 1.43 |
| 2009_IL3 | ISIS EM-BLASSO | 6_480485322 | 6H: 518364045 | -0.24 | 3.71 | 1.75 |
| 2009_IL3 | mrMLM | 7HS_3991657 | 7H: 239608040 | -0.78 | 4.33 | 18.52 |
| 2009_IL3 | FASTmrMLM | 7HS_3991657 | 7H: 239608040 | -0.64 | 3.14 | 12.57 |
| 2009_IL3 | ISIS EM-BLASSO | 7HS_3991657 | 7H: 239608040 | -0.53 | 3.64 | 8.49 |
| 2009_IL4 | mrMLM | M_1778358_754 | 2H: 4629895 | 0.48 | 4.71 | 6.02 |
| 2009_IL4 | FASTmrMLM | M_1778358_754 | 2H: 4629895 | 0.40 | 4.48 | 4.27 |
| 2009_IL4 | pLARmEB | M_1778358_754 | 2H: 4629895 | 0.36 | 3.89 | 3.47 |
| 2009_IL4 | ISIS EM-BLASSO | M_1778358_754 | 2H: 4629895 | 0.35 | 3.58 | 3.26 |
| 2009_IL4 | mrMLM | 3_511071233 | 3H: 635159926 | -0.80 | 10.22 | 12.48 |
| 2009_IL4 | FASTmrMLM | 3_511071233 | 3H: 635159926 | -0.77 | 5.69 | 11.59 |
| 2009_IL4 | pLARmEB | 3_511071233 | 3H: 635159926 | -0.79 | 9.75 | 12.09 |
| 2009_IL4 | ISIS EM-BLASSO | 3_511071233 | 3H: 635159926 | -0.68 | 7.53 | 8.87 |
| 2009_IL4 | mrMLM | 5HL_20437964 | 5H: 487514985 | 0.42 | 3.55 | 3.93 |
| 2009_IL4 | FASTmrMLM | 5HL_20437964 | 5H: 487514985 | 0.33 | 3.26 | 2.44 |
| 2009_IL4 | FASTmrEMMA | M_320650_521 | 5H: 487800967 | 0.68 | 3.77 | 2.62 |
| 2009_IL4 | pLARmEB | M_320650_521 | 5H: 487800967 | 0.32 | 3.59 | 2.39 |
| 2009_IL4 | ISIS EM-BLASSO | 7HS_35651761 | 5H: 525925168 | 0.00 | 4.40 | 0.00 |
| 2009_IL4 | mrMLM | 7HS_21529115 | 7H: 116290148 | 0.54 | 3.40 | 7.63 |
| 2009_IL4 | FASTmrMLM | 7HS_21529115 | 7H: 116290148 | 0.41 | 3.86 | 4.51 |
| 2009_MSL | mrMLM | 1_247319494 | 1H: 364314125 | 0.30 | 5.57 | 3.01 |
| 2009_MSL | FASTmrMLM | 1_247319494 | 1H: 364314125 | 0.26 | 3.36 | 2.24 |
| 2009_MSL | pLARmEB | 1_247319494 | 1H: 364314125 | 0.21 | 5.52 | 1.53 |
| 2009_MSL | pLARmEB | 7HL_2845557 | 1H: 419047094 | 0.00 | 3.01 | 0.00 |
| 2009_MSL | FASTmrEMMA | 2HL_32290916 | 2H: 408126043 | -0.74 | 5.11 | 3.97 |
| 2009_MSL | pLARmEB | 2HL_32290916 | 2H: 408126043 | -0.42 | 3.34 | 6.19 |
| 2009_MSL | mrMLM | 2_399842372 | 2H: 522323980 | -0.46 | 10.16 | 7.45 |
| 2009_MSL | mrMLM | 2HL_34260490 | 2H: 651436685 | 0.40 | 7.85 | 4.51 |
| 2009_MSL | FASTmrMLM | 2HL_34260490 | 2H: 651436685 | 0.38 | 7.47 | 4.02 |
| 2009_MSL | pLARmEB | 2HL_34260490 | 2H: 651436685 | 0.41 | 10.16 | 4.65 |
| 2009_MSL | ISIS EM-BLASSO | 2HL_34260490 | 2H: 651436685 | 0.39 | 6.46 | 4.31 |
| 2009_MSL | pLARmEB | 2_585018981 | 2H: 707356497 | 0.09 | 3.92 | 0.25 |
| 2009_MSL | mrMLM | 2_600749073 | 2H: 727985438 | -0.52 | 14.46 | 9.02 |
| 2009_MSL | FASTmrMLM | 2_600749073 | 2H: 727985438 | -0.50 | 15.62 | 8.43 |
| 2009_MSL | FASTmrEMMA | 2_600749073 | 2H: 727985438 | -1.09 | 11.89 | 8.80 |
| 2009_MSL | pLARmEB | 2_600749073 | 2H: 727985438 | -0.25 | 3.82 | 2.09 |
| 2009_MSL | ISIS EM-BLASSO | 2_600749073 | 2H: 727985438 | -0.46 | 9.88 | 7.10 |
| 2009_MSL | pLARmEB | 2_600662188 | 2H: 728297145 | -0.36 | 4.34 | 4.21 |
| 2009_MSL | ISIS EM-BLASSO | 3_18518805 | 3H: 25691670 | -0.22 | 3.46 | 1.63 |
| 2009_MSL | pLARmEB | 7_41317909 | 7H: 40139310 | -0.10 | 3.17 | 0.33 |
| 2009_MSL | pLARmEB | 7_313370540 | 7H: 261177598 | -0.98 | 6.65 | 32.65 |
| 2009_MSL | mrMLM | M_1649541_1041 | 7H: 271703821 | -0.66 | 4.98 | 14.52 |
| 2009_MSL | FASTmrMLM | M_1649541_1041 | 7H: 271703821 | -0.54 | 4.48 | 9.71 |
| 2009_MSL | mrMLM | M_249593_1037 | 7H: 622802079 | 0.26 | 4.06 | 2.09 |
| 2009_MSL | FASTmrMLM | M_249593_1037 | 7H: 622802079 | 0.21 | 4.32 | 1.35 |
| 2009_MSL | pLARmEB | 7HL_2460896 | 7H: 631357047 | 0.18 | 5.12 | 1.00 |
| 2009_PH | pLARmEB | 2_4900496 | 2H: 5054168 | 1.32 | 4.54 | 1.31 |
| 2009_PH | mrMLM | M_1663886_573 | 2H: 564116957 | -2.07 | 4.28 | 3.22 |
| 2009_PH | FASTmrMLM | M_1663886_573 | 2H: 564116957 | -1.87 | 6.39 | 2.61 |
| 2009_PH | pLARmEB | M_1663886_573 | 2H: 564116957 | -1.78 | 5.66 | 2.37 |
| 2009_PH | ISIS EM-BLASSO | M_1663886_573 | 2H: 564116957 | -1.84 | 4.91 | 2.52 |
| 2009_PH | FASTmrEMMA | 2_522600068 | 2H: 649657420 | 3.49 | 3.86 | 1.70 |
| 2009_PH | pLARmEB | 3_18518805 | 3H: 25691670 | -1.17 | 3.65 | 0.96 |
| 2009_PH | pLARmEB | 3HL_37004393 | 3H: 631870705 | -2.96 | 9.78 | 4.75 |
| 2009_PH | mrMLM | 2HS_32409186 | 3H: 651696476 | -3.34 | 9.77 | 6.73 |
| 2009_PH | FASTmrMLM | 2HS_32409186 | 3H: 651696476 | -3.25 | 9.95 | 6.38 |
| 2009_PH | FASTmrEMMA | 2HS_32409186 | 3H: 651696476 | -7.45 | 9.66 | 8.12 |
| 2009_PH | ISIS EM-BLASSO | 2HS_32409186 | 3H: 651696476 | -3.46 | 12.23 | 7.23 |
| 2009_PH | pLARmEB | M_2038491_176 | 4H: 23190930 | -1.86 | 5.87 | 2.36 |
| 2009_PH | pLARmEB | 4HS_19718929 | 4H: 237182549 | -1.54 | 5.32 | 1.76 |
| 2009_PH | ISIS EM-BLASSO | 4_497278091 | 4H: 596447744 | 1.19 | 3.52 | 1.00 |
| 2009_PH | ISIS EM-BLASSO | 7HS_35651761 | 5H: 525925168 | 1.15 | 3.59 | 0.96 |
| 2009_PH | ISIS EM-BLASSO | 6HL_27943178 | 6H: 370050578 | -1.25 | 3.74 | 1.17 |
| 2009_PH | pLARmEB | 7HS_12212266 | 7H: 81959684 | 1.79 | 4.78 | 2.40 |
| 2009_PH | ISIS EM-BLASSO | 7HS_12212266 | 7H: 81959684 | 1.78 | 4.10 | 2.36 |
| 2009_PH | mrMLM | 7HS_33062962 | 7H: 108670637 | 2.86 | 4.82 | 6.07 |
| 2009_PH | FASTmrMLM | 7HS_33062962 | 7H: 108670637 | 2.62 | 6.60 | 5.11 |
| 2009_PH | mrMLM | 7HS_3991657 | 7H: 239608040 | -3.63 | 3.50 | 9.79 |
| 2009_PH | FASTmrMLM | 7HS_3991657 | 7H: 239608040 | -2.85 | 4.14 | 6.02 |
| 2009_PH | pLARmEB | 7_542100185 | 7H: 598297823 | 0.76 | 3.15 | 0.40 |
| 2009_SLP | FASTmrMLM | 1_41186142 | 1H: 35607649 | -22.38 | 4.72 | 2.28 |
| 2009_SLP | ISIS EM-BLASSO | 2HL_25954643 | 2H: 535670579 | -43.81 | 6.55 | 8.49 |
| 2009_SLP | mrMLM | 2_524762464 | 2H: 649558019 | -112.54 | 21.01 | 42.66 |
| 2009_SLP | FASTmrMLM | 2_524762464 | 2H: 649558019 | -63.75 | 4.51 | 15.21 |
| 2009_SLP | FASTmrEMMA | 2_524762464 | 2H: 649558019 | -212.39 | 19.01 | 40.31 |
| 2009_SLP | ISIS EM-BLASSO | 2_524762464 | 2H: 649558019 | -122.24 | 25.72 | 55.05 |
| 2009_SLP | FASTmrMLM | 2HL_17075593 | 2H: 653982961 | -48.96 | 3.90 | 8.97 |
| 2009_SLP | pLARmEB | 2HL_17075593 | 2H: 653982961 | -56.87 | 3.87 | 11.44 |
| 2009_SLP | FASTmrMLM | 2_624008395 | 2H: 762295561 | -33.05 | 6.05 | 4.69 |
| 2009_SLP | pLARmEB | 2_624008395 | 2H: 762295561 | -30.23 | 4.35 | 3.71 |
| 2009_SLP | FASTmrEMMA | 4_16553551 | 4H: 15498372 | -54.80 | 3.26 | 3.09 |
| 2009_SLP | ISIS EM-BLASSO | 4_16553551 | 4H: 15498372 | -30.19 | 4.50 | 3.92 |
| 2009_SLP | FASTmrMLM | M_114601_946 | 4H: 33779555 | -32.18 | 4.91 | 3.87 |
| 2009_SLP | pLARmEB | M_114601_946 | 4H: 33779555 | -33.90 | 4.84 | 4.06 |
| 2009_SLP | pLARmEB | 4_491047019 | 4H: 590629320 | 28.38 | 4.23 | 3.32 |
| 2009_SLP | FASTmrMLM | 4_497278091 | 4H: 596447744 | 36.05 | 6.83 | 5.58 |
| 2009_SLP | mrMLM | M_69439_714 | 7H: 490849916 | 76.92 | 4.48 | 23.41 |
| 2009_SLP | pLARmEB | 7HL_11788203 | 7H: 516365523 | 47.18 | 3.53 | 9.50 |
| 2009_SLP | FASTmrMLM | 7HL_18029861 | 7H: 517554067 | 52.59 | 5.11 | 12.52 |
| 2009_SMS | ISIS EM-BLASSO | 1H_81523248 | 1H: 370825902 | 2.02 | 3.95 | 1.07 |
| 2009_SMS | pLARmEB | 2_406934594 | 2H: 535680815 | -4.58 | 12.96 | 5.39 |
| 2009_SMS | mrMLM | 2HL_42926196 | 2H: 641432178 | 3.31 | 3.15 | 2.51 |
| 2009_SMS | mrMLM | 2_518992096 | 2H: 646736532 | -5.24 | 3.94 | 6.29 |
| 2009_SMS | mrMLM | Vrs1 | 2H: 652030802 | -16.39 | 6.24 | 66.32 |
| 2009_SMS | FASTmrMLM | Vrs1 | 2H: 652030802 | -18.01 | 49.57 | 80.08 |
| 2009_SMS | FASTmrEMMA | Vrs1 | 2H: 652030802 | -36.05 | 19.47 | 80.21 |
| 2009_SMS | pLARmEB | Vrs1 | 2H: 652030802 | -17.74 | 30.51 | 68.71 |
| 2009_SMS | ISIS EM-BLASSO | Vrs1 | 2H: 652030802 | -19.71 | 59.21 | 84.53 |
| 2009_SMS | pLARmEB | 2_600749073 | 2H: 727985438 | 1.83 | 4.53 | 0.85 |
| 2009_SMS | pLARmEB | 7HL_38070013 | 3H: 229976992 | 1.88 | 4.28 | 0.91 |
| 2009_SMS | pLARmEB | 2HL_30364824 | 3H: 665158698 | -3.29 | 3.43 | 2.19 |
| 2009_SMS | mrMLM | 7_266488249 | 7H: 311430125 | 6.25 | 4.40 | 10.88 |
| 2009_SMS | pLARmEB | 7_570364042 | 7H: 624146130 | 1.72 | 4.18 | 0.68 |
| 2009_SP | mrMLM | 2_6760187 | 2H: 7494521 | 0.63 | 3.65 | 6.11 |
| 2009_SP | pLARmEB | 2HL_32765002 | 2H: 611631175 | 0.77 | 4.00 | 8.72 |
| 2009_SP | FASTmrEMMA | 2_522600068 | 2H: 649657420 | 1.81 | 6.58 | 12.75 |
| 2009_SP | mrMLM | M_124056_833 | 2H: 663628734 | 0.94 | 7.32 | 11.95 |
| 2009_SP | FASTmrMLM | M_124056_833 | 2H: 663628734 | 0.76 | 3.73 | 10.21 |
| 2009_SP | pLARmEB | 3HS_25446223 | 3H: 234821450 | -0.54 | 4.60 | 4.81 |
| 2009_SP | mrMLM | M_9654_521 | 5H: 605138008 | -0.59 | 3.14 | 5.16 |
| 2009_SP | ISIS EM-BLASSO | M_9654_521 | 5H: 605138008 | -0.50 | 4.32 | 4.96 |
| 2009_SP | mrMLM | 7HL_29446583 | 7H: 462916216 | 1.68 | 6.11 | 42.32 |
| 2009_SP | pLARmEB | 7_465676901 | 7H: 502307271 | 0.92 | 4.25 | 14.01 |
| 2009_SP | FASTmrEMMA | 7_492445868 | 7H: 529600430 | 2.17 | 4.11 | 21.00 |
| 2009_SP | ISIS EM-BLASSO | 7HL_17675878 | 7H: 532346138 | 0.82 | 3.70 | 12.91 |
| 2009_TGW | pLARmEB | 1H_31589318 | 1H: 396104564 | -1.21 | 4.81 | 3.03 |
| 2009_TGW | pLARmEB | 2_1447297 | 2H: 304016 | 0.96 | 4.09 | 2.08 |
| 2009_TGW | ISIS EM-BLASSO | 2_1447297 | 2H: 304016 | 1.03 | 3.29 | 2.39 |
| 2009_TGW | mrMLM | Vrs1 | 2H: 652030802 | 5.04 | 26.47 | 46.16 |
| 2009_TGW | FASTmrMLM | Vrs1 | 2H: 652030802 | 5.00 | 4.82 | 46.09 |
| 2009_TGW | FASTmrEMMA | Vrs1 | 2H: 652030802 | 9.87 | 23.84 | 44.89 |
| 2009_TGW | pLARmEB | Vrs1 | 2H: 652030802 | 4.65 | 27.80 | 39.86 |
| 2009_TGW | mrMLM | 3HL_24571314 | 3H: 362303507 | -1.55 | 4.01 | 5.09 |
| 2009_TGW | FASTmrMLM | 3HL_24571314 | 3H: 362303507 | -1.29 | 4.98 | 3.55 |
| 2009_TGW | FASTmrEMMA | 3HL_24571314 | 3H: 362303507 | -2.58 | 3.77 | 3.15 |
| 2009_TGW | pLARmEB | 3HL_24571314 | 3H: 362303507 | -1.15 | 4.13 | 2.86 |
| 2009_TGW | ISIS EM-BLASSO | 3HL_24571314 | 3H: 362303507 | -1.32 | 4.04 | 3.73 |
| 2009_TGW | pLARmEB | 4HS_39123780 | 4H: 351726574 | -1.26 | 4.66 | 3.55 |
| 2009_TGW | ISIS EM-BLASSO | 4HS_39123780 | 4H: 351726574 | -1.37 | 3.86 | 4.20 |
| 2009_TGW | pLARmEB | 7_87712361 | 7H: 74979339 | 1.31 | 4.48 | 3.85 |
| 2009_TGW | FASTmrEMMA | 7HS_30257307 | 7H: 83769522 | 3.02 | 3.67 | 4.29 |
| 2009_TGW | mrMLM | 7HS_33683527 | 7H: 87379560 | 1.57 | 3.26 | 5.49 |
| 2009_TGW | mrMLM | M_152293_651 | 7H: 315449828 | 2.96 | 3.25 | 16.69 |
| 2009_TGW | FASTmrMLM | M_152293_651 | 7H: 315449828 | 2.32 | 3.05 | 10.43 |
| 2009_TGW | pLARmEB | 7HL_8308593 | 7H: 563852248 | 1.40 | 3.69 | 4.41 |
| 2010_GP | FASTmrMLM | 2_524762464 | 2H: 649558019 | -59.22 | 6.23 | 28.89 |
| 2010_GP | FASTmrEMMA | 2_524762464 | 2H: 649558019 | -118.41 | 6.23 | 27.59 |
| 2010_GP | pLARmEB | 2_524762464 | 2H: 649558019 | -60.18 | 7.54 | 29.84 |
| 2010_GP | ISIS EM-BLASSO | 2_524762464 | 2H: 649558019 | -49.45 | 3.46 | 20.15 |
| 2010_GP | mrMLM | 2_527636020 | 2H: 651399477 | -86.73 | 3.37 | 53.35 |
| 2010_GP | mrMLM | 5HL_25927650 | 5H: 241609374 | -22.70 | 3.56 | 3.42 |
| 2010_GP | FASTmrMLM | 5HL_25927650 | 5H: 241609374 | -15.52 | 3.11 | 1.76 |
| 2010_GP | FASTmrEMMA | 5HL_25927650 | 5H: 241609374 | -30.90 | 3.11 | 1.70 |
| 2010_GP | mrMLM | 6_70321538 | 6H: 66806690 | -18.82 | 3.21 | 3.19 |
| 2010_GP | mrMLM | 7HS_13457157 | 7H: 312548378 | 43.51 | 3.01 | 15.83 |
| 2010_GS | pLARmEB | 1H_59728953 | 1H: 388389627 | 1.63 | 5.09 | 1.16 |
| 2010_GS | ISIS EM-BLASSO | 1H_59728953 | 1H: 388389627 | 1.44 | 3.60 | 0.91 |
| 2010_GS | pLARmEB | 2_395906653 | 2H: 510559263 | -1.87 | 5.07 | 1.59 |
| 2010_GS | FASTmrMLM | Vrs1 | 2H: 652030802 | -13.12 | 25.77 | 65.25 |
| 2010_GS | FASTmrEMMA | Vrs1 | 2H: 652030802 | -25.77 | 23.77 | 62.96 |
| 2010_GS | pLARmEB | Vrs1 | 2H: 652030802 | -13.19 | 11.28 | 65.93 |
| 2010_GS | ISIS EM-BLASSO | Vrs1 | 2H: 652030802 | -12.32 | 27.85 | 57.58 |
| 2010_GS | pLARmEB | 2_534773206 | 2H: 664161000 | -2.58 | 3.85 | 2.65 |
| 2010_GS | ISIS EM-BLASSO | 2_534773206 | 2H: 664161000 | -2.59 | 3.23 | 2.66 |
| 2010_GS | pLARmEB | 2_625783669 | 2H: 764361924 | -1.53 | 4.21 | 1.06 |
| 2010_GS | ISIS EM-BLASSO | 2_625783669 | 2H: 764361924 | -1.63 | 4.83 | 1.19 |
| 2010_GWP | ISIS EM-BLASSO | 1H_74333028 | 1H: 22261024 | 0.64 | 4.51 | 3.69 |
| 2010_GWP | mrMLM | 1_271672934 | 1H: 393641404 | -0.90 | 3.44 | 7.50 |
| 2010_GWP | pLARmEB | 2_524762464 | 2H: 649558019 | -0.84 | 6.17 | 5.64 |
| 2010_GWP | ISIS EM-BLASSO | 2HL_43143355 | 2H: 668047196 | -0.90 | 6.40 | 6.71 |
| 2010_GWP | mrMLM | 5_266863847 | 5H: 235681274 | -0.93 | 3.69 | 6.66 |
| 2010_GWP | FASTmrMLM | 5_266863847 | 5H: 235681274 | -0.69 | 3.82 | 3.68 |
| 2010_GWP | pLARmEB | 5_266863847 | 5H: 235681274 | -0.67 | 3.53 | 3.45 |
| 2010_GWP | ISIS EM-BLASSO | M_202999_157 | 5H: 487224801 | 0.00 | 3.15 | 0.00 |
| 2010_GWS | ISIS EM-BLASSO | 1H_84570760 | 1H: 29734717 | 0.07 | 4.27 | 2.22 |
| 2010_GWS | pLARmEB | 1H_62120332 | 1H: 37265716 | -0.06 | 3.95 | 1.51 |
| 2010_GWS | FASTmrMLM | 2HS_20878297 | 2H: 46670382 | 0.07 | 7.06 | 2.25 |
| 2010_GWS | pLARmEB | 2HS_20878297 | 2H: 46670382 | 0.06 | 4.27 | 1.54 |
| 2010_GWS | FASTmrMLM | 2_307625347 | 2H: 461221918 | -0.05 | 4.39 | 1.32 |
| 2010_GWS | pLARmEB | 2HL_37960700 | 2H: 519909276 | -0.08 | 7.96 | 3.41 |
| 2010_GWS | pLARmEB | 2_499268502 | 2H: 632909369 | -0.08 | 4.18 | 2.76 |
| 2010_GWS | pLARmEB | 2_527636020 | 2H: 651399477 | -0.14 | 4.27 | 7.14 |
| 2010_GWS | ISIS EM-BLASSO | 2HL_32227020 | 2H: 659617839 | -0.16 | 3.40 | 10.59 |
| 2010_GWS | FASTmrMLM | 2_531230596 | 2H: 662451129 | -0.17 | 3.85 | 11.83 |
| 2010_GWS | pLARmEB | 2_534773206 | 2H: 664161000 | -0.11 | 3.68 | 5.45 |
| 2010_GWS | pLARmEB | 2_625783669 | 2H: 764361924 | -0.04 | 4.42 | 0.74 |
| 2010_GWS | FASTmrMLM | 3HL_22314417 | 3H: 333448764 | -0.05 | 6.39 | 1.48 |
| 2010_GWS | pLARmEB | 3HL_8562861 | 3H: 352795330 | -0.04 | 4.22 | 0.92 |
| 2010_GWS | FASTmrMLM | 3HL_21083362 | 3H: 468078049 | -0.09 | 7.27 | 2.97 |
| 2010_GWS | ISIS EM-BLASSO | 3_379673146 | 3H: 487356990 | -0.07 | 4.26 | 2.46 |
| 2010_GWS | mrMLM | 2HL_33741786 | 3H: 635892859 | -0.11 | 4.93 | 4.51 |
| 2010_GWS | pLARmEB | 3_529115904 | 3H: 654698488 | -0.12 | 16.26 | 6.01 |
| 2010_GWS | ISIS EM-BLASSO | 3_529115904 | 3H: 654698488 | -0.09 | 5.11 | 3.18 |
| 2010_GWS | pLARmEB | M_90182_4183 | 5H: 629138570 | -0.04 | 3.48 | 0.62 |
| 2010_GWS | mrMLM | 7_75697079 | 7H: 65973576 | 0.11 | 5.96 | 4.96 |
| 2010_GWS | FASTmrMLM | 7_75697079 | 7H: 65973576 | 0.08 | 4.93 | 2.95 |
| 2010_GWS | pLARmEB | 7_75697079 | 7H: 65973576 | 0.08 | 7.58 | 3.26 |
| 2010_GWS | mrMLM | 7HS_37962673 | 7H: 235291724 | -0.32 | 4.75 | 43.54 |
| 2010_GWS | FASTmrMLM | 7HS_37962673 | 7H: 235291724 | -0.16 | 3.08 | 12.31 |
| 2010_GWS | FASTmrMLM | 7_266488249 | 7H: 311430125 | 0.08 | 3.48 | 2.65 |
| 2010_GWS | pLARmEB | M_422728_1186 | 7H: 564137246 | 0.11 | 5.05 | 5.75 |
| 2010_GWS | pLARmEB | 7HL_16064696 | 7H: 586075978 | 0.05 | 4.40 | 1.09 |
| 2010_GWS | pLARmEB | 7_591369444 | 7H: 644255542 | 0.05 | 4.94 | 0.84 |
| 2010_GWS | ISIS EM-BLASSO | 7_591369444 | 7H: 644255542 | 0.07 | 4.73 | 2.21 |
| 2010_IL1 | FASTmrMLM | M_1999039_472 | 2H: 540094243 | -0.38 | 3.09 | 1.20 |
| 2010_IL1 | pLARmEB | M_1999039_472 | 2H: 540094243 | -0.50 | 3.86 | 2.11 |
| 2010_IL1 | FASTmrMLM | M_7336_1752 | 2H: 722298563 | 0.45 | 3.04 | 1.48 |
| 2010_IL1 | pLARmEB | M_7336_1752 | 2H: 722298563 | 0.46 | 3.13 | 1.55 |
| 2010_IL1 | ISIS EM-BLASSO | 3_8289337 | 3H: 9771105 | -0.46 | 3.73 | 1.74 |
| 2010_IL1 | mrMLM | 3HL_25223194 | 3H: 315255195 | -0.88 | 4.39 | 6.17 |
| 2010_IL1 | FASTmrMLM | 3HL_25223194 | 3H: 315255195 | -0.67 | 4.04 | 3.62 |
| 2010_IL1 | pLARmEB | 3HL_6015573 | 3H: 466198066 | -0.72 | 5.76 | 4.15 |
| 2010_IL1 | ISIS EM-BLASSO | 3HL_29995344 | 3H: 647162400 | -0.54 | 3.19 | 1.82 |
| 2010_IL1 | mrMLM | M_1871926_1124 | 6H: 361430799 | -0.78 | 3.39 | 5.06 |
| 2010_IL1 | FASTmrMLM | M_1871926_1124 | 6H: 361430799 | -0.72 | 4.19 | 4.30 |
| 2010_IL1 | FASTmrEMMA | M_1871926_1124 | 6H: 361430799 | -2.46 | 9.45 | 10.64 |
| 2010_IL1 | pLARmEB | M_1871926_1124 | 6H: 361430799 | -0.75 | 4.87 | 4.67 |
| 2010_IL1 | ISIS EM-BLASSO | 6_385523543 | 6H: 413604841 | -1.32 | 13.04 | 14.61 |
| 2010_IL1 | mrMLM | 6HL_29286452 | 6H: 507694407 | -0.81 | 3.65 | 5.35 |
| 2010_IL1 | FASTmrMLM | 6HL_29286452 | 6H: 507694407 | -0.73 | 3.29 | 4.39 |
| 2010_IL1 | pLARmEB | 6HL_29286452 | 6H: 507694407 | -0.67 | 4.00 | 3.73 |
| 2010_IL1 | mrMLM | 7HS_12212266 | 7H: 81959684 | 1.65 | 12.01 | 22.62 |
| 2010_IL1 | FASTmrMLM | 7HS_12212266 | 7H: 81959684 | 1.56 | 12.10 | 20.22 |
| 2010_IL1 | FASTmrEMMA | 7HS_12212266 | 7H: 81959684 | 3.32 | 11.18 | 19.04 |
| 2010_IL1 | pLARmEB | 7HS_12212266 | 7H: 81959684 | 1.52 | 11.81 | 19.29 |
| 2010_IL1 | ISIS EM-BLASSO | 7HS_12212266 | 7H: 81959684 | 1.32 | 9.53 | 14.60 |
| 2010_IL2 | mrMLM | 7_492577145 | 1H: 518317517 | -0.65 | 4.78 | 5.78 |
| 2010_IL2 | pLARmEB | M_253582_2945 | 2H: 175846553 | 0.50 | 5.87 | 2.83 |
| 2010_IL2 | pLARmEB | M_1999039_472 | 2H: 540094243 | -0.46 | 5.27 | 2.38 |
| 2010_IL2 | pLARmEB | 2_499268502 | 2H: 632909369 | -0.22 | 3.48 | 0.43 |
| 2010_IL2 | FASTmrMLM | M_281428_1453 | 3H: 210769136 | 0.51 | 5.40 | 3.48 |
| 2010_IL2 | mrMLM | 3_399923962 | 3H: 312546312 | -1.05 | 4.24 | 14.18 |
| 2010_IL2 | FASTmrEMMA | 3_399923962 | 3H: 312546312 | -1.11 | 8.22 | 3.56 |
| 2010_IL2 | pLARmEB | 3_399923962 | 3H: 312546312 | -0.46 | 5.09 | 2.22 |
| 2010_IL2 | ISIS EM-BLASSO | 3_399923962 | 3H: 312546312 | -0.51 | 5.84 | 3.37 |
| 2010_IL2 | mrMLM | 4HL_23236962 | 3H: 336779957 | 1.17 | 6.26 | 18.36 |
| 2010_IL2 | mrMLM | 3HL_36311844 | 3H: 386796736 | -0.71 | 3.47 | 6.70 |
| 2010_IL2 | FASTmrMLM | 3_309492739 | 3H: 403514405 | -0.96 | 3.51 | 12.31 |
| 2010_IL2 | mrMLM | 3_525094736 | 3H: 649775820 | -1.09 | 4.04 | 13.68 |
| 2010_IL2 | FASTmrMLM | 3_525094736 | 3H: 649775820 | -1.32 | 27.71 | 20.11 |
| 2010_IL2 | FASTmrEMMA | 3HL_38740417 | 3H: 651817377 | -2.43 | 6.04 | 16.13 |
| 2010_IL2 | pLARmEB | 3HL_38740417 | 3H: 651817377 | -1.23 | 30.00 | 13.89 |
| 2010_IL2 | ISIS EM-BLASSO | 3HL_38740417 | 3H: 651817377 | -1.24 | 20.07 | 17.47 |
| 2010_IL2 | FASTmrMLM | 5HL_33488749 | 5H: 317467933 | 0.52 | 8.18 | 3.32 |
| 2010_IL2 | ISIS EM-BLASSO | M_272770_65 | 5H: 454425640 | 0.45 | 4.96 | 2.48 |
| 2010_IL2 | mrMLM | 5HL_49854454 | 5H: 470164909 | 0.57 | 8.69 | 3.87 |
| 2010_IL2 | FASTmrEMMA | 5HL_49854454 | 5H: 470164909 | 0.83 | 5.49 | 1.86 |
| 2010_IL2 | pLARmEB | 5HL_7623791 | 5H: 478872855 | 0.37 | 4.86 | 1.27 |
| 2010_IL2 | FASTmrMLM | 7_483789678 | 7H: 536116501 | -0.68 | 3.68 | 6.31 |
| 2010_IL2 | FASTmrEMMA | 7_483789678 | 7H: 536116501 | -1.50 | 4.48 | 6.29 |
| 2010_IL2 | pLARmEB | 7_483789678 | 7H: 536116501 | -0.62 | 5.40 | 4.29 |
| 2010_IL3 | pLARmEB | 2_579914673 | 2H: 702216890 | -0.29 | 3.03 | 1.51 |
| 2010_IL3 | FASTmrEMMA | 3_511668322 | 3H: 636535362 | -2.30 | 17.94 | 19.23 |
| 2010_IL3 | pLARmEB | 3_511668322 | 3H: 636535362 | -0.96 | 6.53 | 13.65 |
| 2010_IL3 | ISIS EM-BLASSO | 3_511668322 | 3H: 636535362 | -1.21 | 21.75 | 21.70 |
| 2010_IL3 | mrMLM | 3HL_9308548 | 3H: 645265496 | -1.20 | 24.36 | 21.73 |
| 2010_IL3 | FASTmrMLM | 3HL_9308548 | 3H: 645265496 | -0.92 | 6.27 | 12.66 |
| 2010_IL3 | FASTmrEMMA | 5HS_10934051 | 5H: 105264646 | -0.54 | 3.33 | 1.01 |
| 2010_IL3 | pLARmEB | M_168858_235 | 5H: 205032873 | -0.47 | 5.62 | 3.44 |
| 2010_IL3 | mrMLM | 5HL_34845177 | 5H: 278312622 | -0.58 | 5.86 | 4.91 |
| 2010_IL3 | FASTmrMLM | 5HL_34845177 | 5H: 278312622 | -0.42 | 5.37 | 2.62 |
| 2010_IL3 | mrMLM | 5HL_7623791 | 5H: 478872855 | 0.54 | 5.61 | 4.58 |
| 2010_IL3 | FASTmrEMMA | 5HL_7623791 | 5H: 478872855 | 0.68 | 4.36 | 1.72 |
| 2010_IL3 | FASTmrMLM | M_192118_810 | 5H: 487232751 | 0.35 | 5.00 | 2.18 |
| 2010_IL3 | pLARmEB | 5HL_36003694 | 6H: 428146248 | 0.43 | 4.14 | 2.97 |
| 2010_IL3 | mrMLM | 7HL_18906049 | 7H: 486024411 | 0.86 | 5.79 | 13.69 |
| 2010_IL3 | FASTmrMLM | 7HL_18906049 | 7H: 486024411 | 0.79 | 6.28 | 11.33 |
| 2010_IL4 | mrMLM | 2_4900503 | 2H: 4950022 | 0.39 | 4.06 | 2.81 |
| 2010_IL4 | FASTmrMLM | 2_4900503 | 2H: 4950022 | 0.34 | 4.57 | 2.17 |
| 2010_IL4 | FASTmrEMMA | 2_4900503 | 2H: 4950022 | 0.67 | 3.93 | 1.79 |
| 2010_IL4 | pLARmEB | 2_4900503 | 2H: 4950022 | 0.35 | 5.09 | 2.23 |
| 2010_IL4 | ISIS EM-BLASSO | 2_4900503 | 2H: 4950022 | 0.36 | 4.58 | 2.47 |
| 2010_IL4 | pLARmEB | M_1574035_171 | 2H: 724749018 | -0.26 | 3.17 | 1.14 |
| 2010_IL4 | FASTmrMLM | 2_600474867 | 2H: 727925822 | -0.36 | 3.41 | 2.16 |
| 2010_IL4 | ISIS EM-BLASSO | 2_600701693 | 2H: 728016483 | -0.35 | 4.37 | 2.24 |
| 2010_IL4 | mrMLM | 2_600662188 | 2H: 728297145 | -0.57 | 3.84 | 5.66 |
| 2010_IL4 | FASTmrEMMA | 2_600662188 | 2H: 728297145 | -0.80 | 3.70 | 2.46 |
| 2010_IL4 | pLARmEB | 2HL_2279899 | 2H: 754614212 | -0.32 | 3.61 | 1.85 |
| 2010_IL4 | ISIS EM-BLASSO | 2_623828038 | 3H: 197365628 | 0.29 | 3.74 | 1.48 |
| 2010_IL4 | mrMLM | 3HL_37004393 | 3H: 631870705 | -0.84 | 12.18 | 9.57 |
| 2010_IL4 | FASTmrMLM | 3HL_37004393 | 3H: 631870705 | -0.80 | 11.42 | 8.75 |
| 2010_IL4 | ISIS EM-BLASSO | 3HL_37004393 | 3H: 631870705 | -0.76 | 10.09 | 7.88 |
| 2010_IL4 | mrMLM | 4_11810060 | 4H: 10002186 | -0.43 | 4.33 | 3.15 |
| 2010_IL4 | FASTmrMLM | 4_11810060 | 4H: 10002186 | -0.35 | 4.18 | 2.09 |
| 2010_IL4 | FASTmrEMMA | 4_11810060 | 4H: 10002186 | -0.72 | 4.35 | 1.92 |
| 2010_IL4 | ISIS EM-BLASSO | 4_11810060 | 4H: 10002186 | -0.44 | 6.79 | 3.30 |
| 2010_MSL | mrMLM | 1_35132055 | 1H: 20685614 | -0.34 | 6.62 | 3.47 |
| 2010_MSL | FASTmrMLM | 1_35132055 | 1H: 20685614 | -0.31 | 6.61 | 2.91 |
| 2010_MSL | FASTmrEMMA | 1_35132055 | 1H: 20685614 | -0.66 | 4.95 | 2.85 |
| 2010_MSL | pLARmEB | 1_35132055 | 1H: 20685614 | -0.22 | 5.92 | 1.50 |
| 2010_MSL | ISIS EM-BLASSO | M_257577_174 | 2H: 150648140 | -0.37 | 6.28 | 4.10 |
| 2010_MSL | pLARmEB | 2HS_27655610 | 2H: 197618726 | -0.32 | 7.05 | 2.96 |
| 2010_MSL | mrMLM | 2_447773331 | 2H: 560195592 | -0.46 | 5.29 | 6.35 |
| 2010_MSL | FASTmrMLM | 2_447773331 | 2H: 560195592 | -0.34 | 5.75 | 3.46 |
| 2010_MSL | FASTmrEMMA | 2_447773331 | 2H: 560195592 | -0.90 | 8.37 | 5.08 |
| 2010_MSL | ISIS EM-BLASSO | 7HL_36204967 | 2H: 633973348 | 0.31 | 4.64 | 2.05 |
| 2010_MSL | pLARmEB | 2_522610509 | 2H: 648821931 | 0.31 | 6.68 | 2.31 |
| 2010_MSL | mrMLM | 2_600749073 | 2H: 727985438 | -0.74 | 22.85 | 15.54 |
| 2010_MSL | FASTmrMLM | 2_600749073 | 2H: 727985438 | -0.75 | 22.92 | 16.02 |
| 2010_MSL | FASTmrEMMA | 2_600749073 | 2H: 727985438 | -1.49 | 17.77 | 14.04 |
| 2010_MSL | pLARmEB | 2_600749073 | 2H: 727985438 | -0.45 | 3.29 | 5.80 |
| 2010_MSL | ISIS EM-BLASSO | 2_600749073 | 2H: 727985438 | -0.51 | 4.37 | 7.27 |
| 2010_MSL | pLARmEB | 2_600701693 | 2H: 728016483 | -0.39 | 4.64 | 4.52 |
| 2010_MSL | pLARmEB | 3HL_19292679 | 3H: 627356370 | -0.25 | 4.04 | 1.31 |
| 2010_MSL | pLARmEB | 4HL_15111896 | 4H: 514052389 | 0.19 | 4.16 | 1.07 |
| 2010_MSL | ISIS EM-BLASSO | 4HL_15111896 | 4H: 514052389 | 0.14 | 3.19 | 0.55 |
| 2010_MSL | mrMLM | 7HS_423705 | 7H: 318575387 | -0.53 | 4.23 | 8.09 |
| 2010_MSL | FASTmrMLM | 7HS_423705 | 7H: 318575387 | -0.47 | 4.12 | 6.42 |
| 2010_PH | FASTmrEMMA | 1_463006138 | 1H: 554371992 | -2.56 | 3.39 | 0.63 |
| 2010_PH | pLARmEB | 1_463006138 | 1H: 554371992 | -1.44 | 4.21 | 1.09 |
| 2010_PH | mrMLM | M_1999039_472 | 2H: 540094243 | -2.93 | 8.14 | 4.64 |
| 2010_PH | FASTmrMLM | M_1999039_472 | 2H: 540094243 | -2.61 | 8.50 | 3.69 |
| 2010_PH | ISIS EM-BLASSO | M_1999039_472 | 2H: 540094243 | -2.44 | 6.38 | 3.22 |
| 2010_PH | FASTmrEMMA | 2_447773331 | 2H: 560195592 | -5.01 | 8.57 | 2.85 |
| 2010_PH | pLARmEB | 2_447773331 | 2H: 560195592 | -2.63 | 10.42 | 3.69 |
| 2010_PH | mrMLM | 3_511749149 | 3H: 633068955 | -3.95 | 13.79 | 6.53 |
| 2010_PH | FASTmrMLM | 3_511749149 | 3H: 633068955 | -3.89 | 13.70 | 6.35 |
| 2010_PH | pLARmEB | 3_511749149 | 3H: 633068955 | -3.97 | 15.96 | 6.59 |
| 2010_PH | ISIS EM-BLASSO | 2HS_32409186 | 3H: 651696476 | -2.90 | 4.18 | 3.66 |
| 2010_PH | pLARmEB | 6_473168809 | 6H: 516791058 | -1.65 | 5.19 | 1.41 |
| 2010_PH | mrMLM | 6HL_6355520 | 6H: 516860898 | -1.73 | 3.68 | 1.59 |
| 2010_PH | FASTmrMLM | 6HL_6355520 | 6H: 516860898 | -1.42 | 3.48 | 1.08 |
| 2010_PH | FASTmrEMMA | 6HL_6355520 | 6H: 516860898 | -2.79 | 3.69 | 0.94 |
| 2010_PH | ISIS EM-BLASSO | 6HL_6355520 | 6H: 516860898 | -1.67 | 4.11 | 1.49 |
| 2010_PH | mrMLM | 7HS_33062962 | 7H: 108670637 | 2.92 | 4.92 | 4.57 |
| 2010_PH | FASTmrMLM | 7HS_33062962 | 7H: 108670637 | 2.55 | 5.35 | 3.49 |
| 2010_PH | FASTmrEMMA | 7HS_33062962 | 7H: 108670637 | 4.53 | 4.31 | 2.31 |
| 2010_PH | pLARmEB | 7HS_33062962 | 7H: 108670637 | 2.23 | 4.58 | 2.67 |
| 2010_SLP | FASTmrMLM | 2_522710996 | 2H: 647661551 | -61.41 | 3.91 | 13.43 |
| 2010_SLP | FASTmrEMMA | 2_522710996 | 2H: 647661551 | -147.03 | 5.32 | 18.55 |
| 2010_SLP | pLARmEB | 2_524762464 | 2H: 649558019 | -60.80 | 4.74 | 14.87 |
| 2010_SLP | pLARmEB | 2HL_17075593 | 2H: 653982961 | -68.55 | 5.90 | 18.90 |
| 2010_SLP | ISIS EM-BLASSO | 2HL_17075593 | 2H: 653982961 | -58.99 | 3.82 | 14.00 |
| 2010_SLP | FASTmrMLM | 2_534864652 | 2H: 659635806 | -76.70 | 7.00 | 24.05 |
| 2010_SLP | FASTmrEMMA | 2_534864652 | 2H: 659635806 | -135.02 | 5.19 | 17.95 |
| 2010_SLP | pLARmEB | 2_585267345 | 2H: 710337347 | -21.40 | 3.26 | 1.96 |
| 2010_SLP | ISIS EM-BLASSO | 2_585267345 | 2H: 710337347 | -20.25 | 3.12 | 1.75 |
| 2010_SLP | ISIS EM-BLASSO | M_1605646_794 | 4H: 16761959 | -24.30 | 4.35 | 2.82 |
| 2010_SLP | mrMLM | 4HS_28831116 | 4H: 22582088 | -40.22 | 4.34 | 7.26 |
| 2010_SLP | FASTmrMLM | 4HS_28831116 | 4H: 22582088 | -21.54 | 3.08 | 2.08 |
| 2010_SLP | pLARmEB | 4HS_28831116 | 4H: 22582088 | -27.31 | 4.67 | 3.35 |
| 2010_SMS | pLARmEB | 2HS_33824362 | 2H: 131796550 | -2.70 | 5.25 | 1.42 |
| 2010_SMS | ISIS EM-BLASSO | 2_406934594 | 2H: 535680815 | -4.20 | 5.68 | 3.91 |
| 2010_SMS | mrMLM | 2_527636020 | 2H: 651399477 | -5.48 | 3.71 | 5.35 |
| 2010_SMS | mrMLM | Vrs1 | 2H: 652030802 | -18.81 | 15.94 | 66.62 |
| 2010_SMS | FASTmrMLM | Vrs1 | 2H: 652030802 | -19.53 | 35.93 | 71.75 |
| 2010_SMS | FASTmrEMMA | Vrs1 | 2H: 652030802 | -43.84 | 35.22 | 90.41 |
| 2010_SMS | pLARmEB | Vrs1 | 2H: 652030802 | -21.20 | 57.22 | 71.96 |
| 2010_SMS | ISIS EM-BLASSO | Vrs1 | 2H: 652030802 | -21.69 | 56.11 | 88.54 |
| 2010_SMS | mrMLM | 2_530198498 | 2H: 659234271 | 3.46 | 4.30 | 2.21 |
| 2010_SMS | pLARmEB | 2_534773206 | 2H: 664161000 | -2.47 | 4.54 | 1.02 |
| 2010_SMS | ISIS EM-BLASSO | 2_534773206 | 2H: 664161000 | -2.39 | 3.91 | 1.13 |
| 2010_SMS | mrMLM | M_146190_773 | 2H: 676005041 | -4.62 | 3.07 | 4.16 |
| 2010_SMS | mrMLM | 2_558070055 | 2H: 678622445 | 3.31 | 3.08 | 1.98 |
| 2010_SMS | ISIS EM-BLASSO | M_1605646_794 | 4H: 16761959 | -1.41 | 3.40 | 0.45 |
| 2010_SMS | mrMLM | 4_121425641 | 4H: 78265040 | 2.20 | 4.76 | 1.06 |
| 2010_SMS | FASTmrMLM | 4_121425641 | 4H: 78265040 | 1.62 | 3.57 | 0.58 |
| 2010_SMS | pLARmEB | M_78036_3459 | 4H: 83755281 | 1.82 | 4.54 | 0.64 |
| 2010_SMS | ISIS EM-BLASSO | 4_107657424 | 4H: 94940577 | 1.41 | 3.35 | 0.46 |
| 2010_SMS | pLARmEB | 7_266488249 | 7H: 311430125 | 3.64 | 5.63 | 2.39 |
| 2010_SP | pLARmEB | M_254645_1263 | 2H: 468557461 | 0.27 | 3.49 | 1.29 |
| 2010_SP | mrMLM | Vrs1 | 2H: 652030802 | 1.41 | 23.32 | 31.20 |
| 2010_SP | FASTmrMLM | Vrs1 | 2H: 652030802 | 1.39 | 24.31 | 36.68 |
| 2010_SP | FASTmrEMMA | Vrs1 | 2H: 652030802 | 2.77 | 10.71 | 47.33 |
| 2010_SP | pLARmEB | Vrs1 | 2H: 652030802 | 1.64 | 30.41 | 39.81 |
| 2010_SP | ISIS EM-BLASSO | Vrs1 | 2H: 652030802 | 1.49 | 22.14 | 52.32 |
| 2010_SP | pLARmEB | 2_590708572 | 2H: 719198330 | -0.41 | 4.66 | 2.73 |
| 2010_SP | ISIS EM-BLASSO | 2_590708572 | 2H: 719198330 | -0.41 | 3.77 | 4.52 |
| 2010_SP | mrMLM | 5HL_31102351 | 5H: 259786971 | -0.48 | 3.55 | 3.30 |
| 2010_SP | FASTmrMLM | 5HL_31102351 | 5H: 259786971 | -0.36 | 4.62 | 2.27 |
| 2010_SP | mrMLM | 7HS_6212658 | 7H: 245851013 | -1.16 | 4.91 | 25.60 |
| 2010_SP | FASTmrMLM | 7HS_6212658 | 7H: 245851013 | -0.94 | 4.86 | 20.37 |
| 2010_SP | pLARmEB | 7HS_6212658 | 7H: 245851013 | -0.80 | 4.40 | 11.45 |
| 2010_SP | mrMLM | 7HL_31918207 | 7H: 422680039 | -0.99 | 3.41 | 18.05 |
| 2010_SP | FASTmrMLM | 7HL_31918207 | 7H: 422680039 | -0.70 | 4.04 | 11.00 |
| 2010_TGW | pLARmEB | 1H_75978121 | 1H: 21014205 | 0.92 | 4.55 | 0.95 |
| 2010_TGW | pLARmEB | 1H_74200878 | 1H: 180108719 | -1.18 | 6.01 | 1.61 |
| 2010_TGW | ISIS EM-BLASSO | 1H_62017645 | 1H: 264500454 | -0.93 | 3.17 | 1.01 |
| 2010_TGW | FASTmrMLM | Vrs1 | 2H: 652030802 | 7.48 | 43.57 | 54.79 |
| 2010_TGW | FASTmrEMMA | Vrs1 | 2H: 652030802 | 15.22 | 7.37 | 56.66 |
| 2010_TGW | pLARmEB | Vrs1 | 2H: 652030802 | 5.79 | 16.10 | 32.77 |
| 2010_TGW | ISIS EM-BLASSO | Vrs1 | 2H: 652030802 | 7.57 | 49.42 | 56.12 |
| 2010_TGW | pLARmEB | 2HL_22930005 | 2H: 652508158 | 2.07 | 3.58 | 3.95 |
| 2010_TGW | mrMLM | 2HL_39317790 | 2H: 659768675 | 7.27 | 5.69 | 49.00 |
| 2010_TGW | mrMLM | 3HS_23539468 | 3H: 272283784 | -1.89 | 3.89 | 3.78 |
| 2010_TGW | FASTmrMLM | 3HS_23539468 | 3H: 272283784 | -1.55 | 4.84 | 2.52 |
| 2010_TGW | ISIS EM-BLASSO | 4HL_35832908 | 4H: 545875758 | 1.40 | 5.73 | 2.24 |
| 2010_TGW | pLARmEB | 3HL_44939929 | 5H: 285041356 | -0.99 | 5.15 | 0.97 |
| 2010_TGW | pLARmEB | M_90182_4183 | 5H: 629138570 | -1.74 | 10.61 | 3.64 |
| 2010_TGW | mrMLM | 7HS_10887541 | 7H: 72344563 | 3.06 | 6.94 | 11.22 |
| 2010_TGW | FASTmrMLM | 7HS_10887541 | 7H: 72344563 | 2.44 | 8.79 | 7.11 |
| 2010_TGW | FASTmrEMMA | 7HS_10887541 | 7H: 72344563 | 5.05 | 8.93 | 7.15 |
| 2010_TGW | pLARmEB | 7HS_10887541 | 7H: 72344563 | 2.31 | 13.16 | 6.37 |
| 2010_TGW | ISIS EM-BLASSO | 7HS_10887541 | 7H: 72344563 | 2.37 | 10.46 | 6.70 |
| 2012_GP | ISIS EM-BLASSO | 2HL_4678325 | 2H: 646072880 | -111.37 | 6.65 | 16.42 |
| 2012_GP | mrMLM | Vrs1 | 2H: 652030802 | -119.91 | 7.31 | 21.00 |
| 2012_GP | FASTmrMLM | Vrs1 | 2H: 652030802 | -109.74 | 7.31 | 17.59 |
| 2012_GP | FASTmrEMMA | Vrs1 | 2H: 652030802 | -219.41 | 7.31 | 17.58 |
| 2012_GP | pLARmEB | 2_534686550 | 2H: 663878010 | -108.81 | 6.13 | 17.76 |
| 2012_GS | pLARmEB | 2_524762464 | 2H: 649558019 | -5.51 | 3.04 | 16.51 |
| 2012_GS | FASTmrMLM | Vrs1 | 2H: 652030802 | -9.96 | 25.21 | 53.95 |
| 2012_GS | ISIS EM-BLASSO | Vrs1 | 2H: 652030802 | -9.59 | 26.98 | 49.99 |
| 2012_GS | pLARmEB | 6HS_22677660 | 6H: 46063198 | -2.73 | 3.91 | 4.90 |
| 2012_GS | ISIS EM-BLASSO | 6HS_22677660 | 6H: 46063198 | -2.38 | 3.49 | 3.70 |
| 2012_IL1 | ISIS EM-BLASSO | 1H_43983274 | 1H: 76456545 | 0.87 | 5.50 | 4.91 |
| 2012_IL1 | mrMLM | M_173442_1610 | 1H: 285199675 | 0.78 | 4.31 | 3.10 |
| 2012_IL1 | FASTmrMLM | M_173442_1610 | 1H: 285199675 | 0.62 | 5.02 | 2.27 |
| 2012_IL1 | pLARmEB | M_173442_1610 | 1H: 285199675 | 0.58 | 4.98 | 2.20 |
| 2012_IL1 | mrMLM | 2_399823529 | 2H: 521774247 | -0.77 | 4.49 | 3.12 |
| 2012_IL1 | FASTmrMLM | 2_399823529 | 2H: 521774247 | -0.61 | 5.03 | 2.26 |
| 2012_IL1 | FASTmrEMMA | 2_399823529 | 2H: 521774247 | -1.31 | 3.23 | 2.38 |
| 2012_IL1 | ISIS EM-BLASSO | 2HL_18970523 | 2H: 647935163 | 0.72 | 4.01 | 2.84 |
| 2012_IL1 | pLARmEB | 2_524762464 | 2H: 649558019 | 1.22 | 3.46 | 8.27 |
| 2012_IL1 | ISIS EM-BLASSO | 3HL_25561608 | 2H: 704382692 | -0.74 | 4.45 | 3.57 |
| 2012_IL1 | pLARmEB | 2_581587377 | 2H: 708489886 | -0.52 | 6.25 | 1.59 |
| 2012_IL1 | mrMLM | 2_625783669 | 2H: 764361924 | -0.68 | 3.38 | 2.42 |
| 2012_IL1 | FASTmrMLM | 2_625783669 | 2H: 764361924 | -0.51 | 3.10 | 1.58 |
| 2012_IL1 | pLARmEB | 2_625783669 | 2H: 764361924 | -0.79 | 5.92 | 4.12 |
| 2012_IL1 | pLARmEB | 3HS_24205580 | 3H: 271139361 | -0.77 | 6.23 | 3.65 |
| 2012_IL1 | mrMLM | M_1613272_1572 | 3H: 325148517 | -1.42 | 12.11 | 10.24 |
| 2012_IL1 | FASTmrEMMA | 6HL_26059439 | 6H: 486288685 | -2.97 | 9.01 | 13.56 |
| 2012_IL1 | pLARmEB | 6_451975519 | 6H: 492894732 | -0.94 | 4.87 | 5.81 |
| 2012_IL1 | ISIS EM-BLASSO | 6_451975519 | 6H: 492894732 | -0.98 | 3.56 | 6.36 |
| 2012_IL1 | mrMLM | 4_79532838 | 6H: 499250546 | -1.94 | 20.94 | 19.78 |
| 2012_IL1 | FASTmrMLM | 4_79532838 | 6H: 499250546 | -1.82 | 21.49 | 19.71 |
| 2012_IL1 | mrMLM | 7HS_21829337 | 7H: 81889341 | 1.22 | 7.38 | 7.93 |
| 2012_IL1 | FASTmrMLM | 7HS_21829337 | 7H: 81889341 | 1.14 | 7.28 | 7.85 |
| 2012_IL1 | pLARmEB | 7HS_21829337 | 7H: 81889341 | 1.33 | 10.87 | 11.75 |
| 2012_IL1 | FASTmrEMMA | 7HS_31244294 | 7H: 82552493 | 2.06 | 3.82 | 5.61 |
| 2012_IL1 | ISIS EM-BLASSO | 7HS_33683527 | 7H: 87379560 | 0.85 | 3.67 | 4.90 |
| 2012_IL1 | mrMLM | 7HL_35226091 | 7H: 351767493 | -1.98 | 4.78 | 19.98 |
| 2012_IL1 | FASTmrMLM | 7HL_35226091 | 7H: 351767493 | -1.82 | 4.91 | 19.02 |
| 2012_IL1 | mrMLM | 7HL_25416678 | 7H: 479848646 | 1.78 | 5.18 | 16.61 |
| 2012_IL1 | FASTmrMLM | 7HL_25416678 | 7H: 479848646 | 1.59 | 5.62 | 15.14 |
| 2012_IL1 | pLARmEB | 7_522300442 | 7H: 580344802 | -1.01 | 7.15 | 6.66 |
| 2012_IL1 | mrMLM | 7_575487388 | 7H: 627311039 | 0.80 | 4.70 | 3.10 |
| 2012_IL1 | FASTmrMLM | 7_575487388 | 7H: 627311039 | 0.63 | 4.96 | 2.18 |
| 2012_IL1 | pLARmEB | 7_575487388 | 7H: 627311039 | 0.87 | 7.04 | 4.64 |
| 2012_IL1 | ISIS EM-BLASSO | 7_575487388 | 7H: 627311039 | 0.51 | 3.11 | 1.60 |
| 2012_IL2 | mrMLM | M_114215_455 | 7H: 258071311 | -5.24 | 7.27 | 55.77 |
| 2012_IL2 | FASTmrMLM | M_114215_455 | 7H: 258071311 | -3.83 | 6.80 | 51.34 |
| 2012_IL2 | FASTmrEMMA | M_114215_455 | 7H: 258071311 | -7.66 | 5.16 | 49.41 |
| 2012_IL2 | mrMLM | 7HL_15041022 | 7H: 362239935 | 3.12 | 3.03 | 19.48 |
| 2012_IL3 | mrMLM | 5HL_40513680 | 2H: 59001348 | -0.57 | 4.06 | 5.34 |
| 2012_IL3 | FASTmrMLM | 5HL_40513680 | 2H: 59001348 | -0.38 | 3.04 | 2.38 |
| 2012_IL3 | mrMLM | 2_600662188 | 2H: 728297145 | -0.50 | 3.34 | 3.86 |
| 2012_IL3 | pLARmEB | 2_600662188 | 2H: 728297145 | -0.43 | 3.18 | 2.92 |
| 2012_IL3 | ISIS EM-BLASSO | 3HL_15958290 | 3H: 468058138 | -0.69 | 4.70 | 6.12 |
| 2012_IL3 | ISIS EM-BLASSO | 3HL_22407667 | 3H: 623871879 | -0.61 | 3.12 | 5.04 |
| 2012_IL3 | mrMLM | 3_511749149 | 3H: 633068955 | -1.12 | 12.68 | 16.08 |
| 2012_IL3 | FASTmrMLM | 3_511749149 | 3H: 633068955 | -1.09 | 4.66 | 15.21 |
| 2012_IL3 | pLARmEB | 3_511668322 | 3H: 636535362 | -1.20 | 13.60 | 17.89 |
| 2012_IL3 | ISIS EM-BLASSO | 6_18118681 | 6H: 17542081 | 0.37 | 3.15 | 2.17 |
| 2012_IL3 | FASTmrEMMA | 7_28183341 | 7H: 29996800 | 0.88 | 3.43 | 2.72 |
| 2012_IL3 | mrMLM | M_45575_2028 | 7H: 362186578 | 1.02 | 3.12 | 15.28 |
| 2012_IL4 | pLARmEB | M_442637_386 | 1H: 318101615 | 0.64 | 3.53 | 3.51 |
| 2012_IL4 | pLARmEB | 3_500246440 | 3H: 625629121 | -1.04 | 6.03 | 7.78 |
| 2012_IL4 | FASTmrMLM | 3_511668322 | 3H: 636535362 | -1.07 | 6.37 | 5.73 |
| 2012_IL4 | ISIS EM-BLASSO | 3HL_11611908 | 3H: 641856204 | 0.00 | 4.35 | 0.00 |
| 2012_IL4 | FASTmrMLM | 7_24486635 | 7H: 23245860 | 0.72 | 4.80 | 3.45 |
| 2012_IL4 | FASTmrMLM | 2_287569753 | 7H: 265478343 | -2.13 | 5.67 | 28.60 |
| 2012_IL4 | mrMLM | 7HS_30003071 | 7H: 270546724 | -1.99 | 5.42 | 25.81 |
| 2012_IL4 | FASTmrMLM | 7HS_30003071 | 7H: 270546724 | -1.56 | 4.00 | 15.24 |
| 2012_IL4 | FASTmrEMMA | 7HL_11281033 | 7H: 360793216 | -3.19 | 3.00 | 18.98 |
| 2012_IL4 | pLARmEB | 7HL_11281033 | 7H: 360793216 | -1.59 | 3.46 | 19.57 |
| 2012_IL4 | mrMLM | 7HL_18660410 | 7H: 477578189 | -1.96 | 4.00 | 25.10 |
| 2012_IL4 | FASTmrMLM | 7HL_18660410 | 7H: 477578189 | -1.55 | 4.94 | 15.06 |
| 2012_MSL | pLARmEB | 1H_26181065 | 1H: 214888381 | -0.19 | 3.39 | 0.87 |
| 2012_MSL | mrMLM | 1H_44859655 | 1H: 390861283 | 0.40 | 3.91 | 4.52 |
| 2012_MSL | FASTmrMLM | 1H_44859655 | 1H: 390861283 | 0.31 | 3.66 | 2.68 |
| 2012_MSL | FASTmrEMMA | 1H_44859655 | 1H: 390861283 | 0.56 | 3.15 | 1.92 |
| 2012_MSL | pLARmEB | 1H_44859655 | 1H: 390861283 | 0.49 | 9.32 | 5.95 |
| 2012_MSL | ISIS EM-BLASSO | 1_276506071 | 1H: 395406283 | 0.31 | 3.77 | 2.64 |
| 2012_MSL | pLARmEB | 2_305360698 | 2H: 469269219 | -0.36 | 4.39 | 3.46 |
| 2012_MSL | mrMLM | M_223185_603 | 2H: 473725143 | -0.54 | 3.91 | 8.64 |
| 2012_MSL | FASTmrMLM | M_223185_603 | 2H: 473725143 | -0.39 | 5.00 | 4.53 |
| 2012_MSL | ISIS EM-BLASSO | 2HL_32158541 | 2H: 475151803 | -0.59 | 10.97 | 10.32 |
| 2012_MSL | pLARmEB | 2_522610509 | 2H: 648821931 | 0.27 | 3.61 | 1.57 |
| 2012_MSL | mrMLM | 2_600749073 | 2H: 727985438 | -0.73 | 13.09 | 15.01 |
| 2012_MSL | FASTmrMLM | 2_600749073 | 2H: 727985438 | -0.72 | 12.72 | 14.49 |
| 2012_MSL | ISIS EM-BLASSO | 2_600749073 | 2H: 727985438 | -0.89 | 17.33 | 22.38 |
| 2012_MSL | pLARmEB | 2_600701693 | 2H: 728016483 | -0.60 | 13.46 | 9.48 |
| 2012_MSL | pLARmEB | 7_549761498 | 5H: 645820225 | 0.37 | 6.47 | 3.36 |
| 2012_MSL | pLARmEB | 7_521962601 | 7H: 564740349 | -0.42 | 3.50 | 4.69 |
| 2012_PH | mrMLM | 1_463006138 | 1H: 554371992 | -2.59 | 6.64 | 4.07 |
| 2012_PH | FASTmrMLM | 1_463006138 | 1H: 554371992 | -1.96 | 5.32 | 2.32 |
| 2012_PH | FASTmrEMMA | 1_463006138 | 1H: 554371992 | -3.82 | 4.48 | 1.62 |
| 2012_PH | pLARmEB | 1_463006138 | 1H: 554371992 | -1.96 | 5.23 | 2.33 |
| 2012_PH | ISIS EM-BLASSO | 1_463006138 | 1H: 554371992 | -1.64 | 4.31 | 1.62 |
| 2012_PH | mrMLM | 2HL_29744782 | 2H: 407748829 | -1.95 | 5.06 | 2.38 |
| 2012_PH | FASTmrMLM | 2HL_29744782 | 2H: 407748829 | -2.09 | 5.25 | 2.71 |
| 2012_PH | pLARmEB | 2HL_29744782 | 2H: 407748829 | -2.18 | 5.54 | 2.96 |
| 2012_PH | mrMLM | 2HL_17075593 | 2H: 653982961 | 2.26 | 5.79 | 2.59 |
| 2012_PH | FASTmrMLM | 2HL_17075593 | 2H: 653982961 | 1.43 | 3.40 | 1.05 |
| 2012_PH | FASTmrEMMA | 2HL_17075593 | 2H: 653982961 | 3.83 | 4.88 | 1.79 |
| 2012_PH | mrMLM | 3_511749149 | 3H: 633068955 | -3.87 | 3.38 | 7.23 |
| 2012_PH | FASTmrMLM | M_180113_90 | 3H: 649810957 | -3.77 | 11.75 | 7.23 |
| 2012_PH | pLARmEB | M_180113_90 | 3H: 649810957 | -2.69 | 4.14 | 3.69 |
| 2012_PH | FASTmrEMMA | 2HS_32409186 | 3H: 651696476 | -7.63 | 11.12 | 7.07 |
| 2012_PH | ISIS EM-BLASSO | 2HS_32409186 | 3H: 651696476 | -4.25 | 14.09 | 9.03 |
| 2012_PH | ISIS EM-BLASSO | 5_149952696 | 5H: 86005119 | -1.51 | 3.88 | 1.18 |
| 2012_PH | ISIS EM-BLASSO | 6HL_32688979 | 6H: 420180564 | -2.15 | 5.83 | 2.88 |
| 2012_PH | mrMLM | 6_466015037 | 6H: 502018934 | -2.99 | 9.89 | 5.41 |
| 2012_PH | FASTmrMLM | 6_466015037 | 6H: 502018934 | -2.62 | 8.81 | 4.15 |
| 2012_PH | FASTmrEMMA | 6_466015037 | 6H: 502018934 | -3.74 | 4.56 | 1.87 |
| 2012_PH | pLARmEB | 6_466015037 | 6H: 502018934 | -2.42 | 7.55 | 3.54 |
| 2012_PH | mrMLM | 7_217306166 | 7H: 295389015 | -3.93 | 3.25 | 8.89 |
| 2012_PH | mrMLM | M_249593_1037 | 7H: 622802079 | 2.21 | 5.11 | 2.68 |
| 2012_PH | FASTmrMLM | M_249593_1037 | 7H: 622802079 | 1.33 | 3.38 | 0.98 |
| 2012_SLP | pLARmEB | 1_456568890 | 1H: 548913487 | -32.10 | 3.27 | 1.23 |
| 2012_SLP | pLARmEB | 2HL_27432067 | 2H: 547091788 | -52.18 | 5.06 | 3.48 |
| 2012_SLP | FASTmrEMMA | 2_522610509 | 2H: 648821931 | 186.87 | 3.53 | 6.62 |
| 2012_SLP | ISIS EM-BLASSO | 2_522610509 | 2H: 648821931 | 100.80 | 4.08 | 7.93 |
| 2012_SLP | mrMLM | Vrs1 | 2H: 652030802 | -211.36 | 3.32 | 29.16 |
| 2012_SLP | FASTmrMLM | Vrs1 | 2H: 652030802 | -286.97 | 17.19 | 68.19 |
| 2012_SLP | FASTmrEMMA | Vrs1 | 2H: 652030802 | -605.54 | 17.32 | 72.21 |
| 2012_SLP | pLARmEB | Vrs1 | 2H: 652030802 | -233.18 | 35.66 | 57.20 |
| 2012_SLP | ISIS EM-BLASSO | Vrs1 | 2H: 652030802 | -302.28 | 18.50 | 71.33 |
| 2012_SLP | pLARmEB | 3HL_41229929 | 3H: 427116027 | 35.84 | 3.34 | 1.56 |
| 2012_SLP | ISIS EM-BLASSO | 4HS_8953586 | 4H: 8747991 | -47.90 | 3.84 | 2.07 |
| 2012_SLP | pLARmEB | 4HS_28564354 | 4H: 16168735 | -54.72 | 6.00 | 3.51 |
| 2012_SLP | mrMLM | 7HS_30883454 | 7H: 260315190 | 148.21 | 4.21 | 16.73 |
| 2012_SLP | mrMLM | 7HS_24054991 | 7H: 308998830 | -225.50 | 3.41 | 39.00 |
| 2012_SMS | ISIS EM-BLASSO | 1H_40034863 | 1H: 379422580 | 2.46 | 5.43 | 1.31 |
| 2012_SMS | mrMLM | 1H_40034223 | 1H: 379423220 | 2.67 | 4.20 | 1.54 |
| 2012_SMS | FASTmrMLM | 1H_40034223 | 1H: 379423220 | 2.24 | 4.01 | 1.10 |
| 2012_SMS | pLARmEB | 1H_40034223 | 1H: 379423220 | 2.81 | 7.87 | 1.58 |
| 2012_SMS | mrMLM | 2HL_35761605 | 2H: 472012264 | -4.68 | 9.21 | 4.80 |
| 2012_SMS | FASTmrMLM | 2HL_35761605 | 2H: 472012264 | -4.34 | 9.35 | 4.20 |
| 2012_SMS | ISIS EM-BLASSO | 2HL_35761605 | 2H: 472012264 | -4.45 | 10.00 | 4.33 |
| 2012_SMS | mrMLM | Vrs1 | 2H: 652030802 | -21.82 | 19.99 | 86.00 |
| 2012_SMS | FASTmrMLM | Vrs1 | 2H: 652030802 | -21.68 | 32.99 | 86.32 |
| 2012_SMS | FASTmrEMMA | Vrs1 | 2H: 652030802 | -35.83 | 22.00 | 65.07 |
| 2012_SMS | pLARmEB | Vrs1 | 2H: 652030802 | -21.32 | 68.02 | 76.19 |
| 2012_SMS | ISIS EM-BLASSO | Vrs1 | 2H: 652030802 | -21.91 | 64.75 | 86.43 |
| 2012_SMS | pLARmEB | 3HL_21234462 | 3H: 317723222 | 1.36 | 3.06 | 0.36 |
| 2012_SMS | pLARmEB | 4HS_22370471 | 4H: 22470689 | -1.87 | 3.82 | 0.66 |
| 2012_SP | pLARmEB | 2_522610509 | 2H: 648821931 | 0.85 | 4.02 | 7.61 |
| 2012_SP | mrMLM | 2_531255437 | 2H: 662335248 | 1.02 | 3.83 | 10.91 |
| 2012_SP | FASTmrMLM | 2_531255437 | 2H: 662335248 | 0.81 | 4.68 | 6.88 |
| 2012_SP | FASTmrEMMA | 2_531255437 | 2H: 662335248 | 1.76 | 4.80 | 7.51 |
| 2012_SP | ISIS EM-BLASSO | 2_531255437 | 2H: 662335248 | 0.79 | 3.96 | 6.49 |
| 2012_SP | FASTmrEMMA | 3HS_30113020 | 3H: 64796275 | 1.25 | 3.53 | 4.29 |
| 2012_SP | mrMLM | 4HS_34129830 | 4H: 582682475 | -1.03 | 4.32 | 12.96 |
| 2012_SP | FASTmrMLM | 4HS_34129830 | 4H: 582682475 | -0.85 | 4.38 | 8.78 |
| 2012_SP | pLARmEB | 4HS_34129830 | 4H: 582682475 | -0.80 | 3.76 | 7.85 |
| 2012_SP | ISIS EM-BLASSO | 4_519876228 | 4H: 618857638 | -0.81 | 3.99 | 7.63 |
| 2012_SP | FASTmrEMMA | 4_519670615 | 4H: 619186445 | -1.98 | 5.97 | 10.61 |
| BLUP_GP | pLARmEB | 2_619663942 | 2H: 755106750 | 8.96 | 3.18 | 2.26 |
| BLUP_GP | ISIS EM-BLASSO | 6HS_4428068 | 6H: 149054125 | -12.42 | 3.61 | 4.28 |
| BLUP_GP | ISIS EM-BLASSO | M_1589358_1352 | 2H: 650438830 | -39.38 | 17.05 | 35.35 |
| BLUP_GP | mrMLM | M_1589358_1352 | 2H: 650438830 | -40.53 | 4.82 | 37.44 |
| BLUP_GP | pLARmEB | M_1589358_1352 | 2H: 650438830 | -29.30 | 5.43 | 19.56 |
| BLUP_GP | FASTmrMLM | vrs1 | 2H: 652030802 | -38.61 | 16.71 | 34.59 |
| BLUP_GS | pLARmEB | 1H_17722634 | 1H: 558131353 | -0.78 | 3.89 | 0.76 |
| BLUP_GS | ISIS EM-BLASSO | 1H_59728953 | 1H: 388389627 | 1.43 | 4.47 | 2.48 |
| BLUP_GS | pLARmEB | 1H_7826740 | 1H: 382736522 | 1.26 | 7.32 | 1.89 |
| BLUP_GS | pLARmEB | 2_307625347 | 2H: 461221918 | -1.32 | 7.29 | 2.24 |
| BLUP_GS | mrMLM | 2_524762464 | 2H: 649558019 | -8.27 | 3.07 | 71.50 |
| BLUP_GS | pLARmEB | 2_524762464 | 2H: 649558019 | -3.06 | 5.00 | 9.77 |
| BLUP_GS | pLARmEB | 2_598715827 | 2H: 729178615 | 1.35 | 8.18 | 2.19 |
| BLUP_GS | ISIS EM-BLASSO | 5_428226858 | 5H: 522253972 | -1.16 | 4.60 | 1.70 |
| BLUP_GS | FASTmrMLM | 5_554996602 | 7H: 30660407 | 1.36 | 3.27 | 2.10 |
| BLUP_GS | pLARmEB | 7_259742316 | 2H: 266352970 | -2.57 | 4.87 | 8.24 |
| BLUP_GS | pLARmEB | M_122697_158 | 1H: 82701585 | -0.73 | 4.87 | 0.68 |
| BLUP_GS | pLARmEB | M_72984_1488 | 7H: 656211646 | 1.09 | 6.38 | 1.33 |
| BLUP_GS | FASTmrMLM | vrs1 | 2H: 652030802 | -8.31 | 22.14 | 72.25 |
| BLUP_GS | ISIS EM-BLASSO | vrs1 | 2H: 652030802 | -7.90 | 48.96 | 65.23 |
| BLUP_GW | pLARmEB | 1_456568890 | 1H: 548913487 | -0.27 | 4.29 | 1.85 |
| BLUP_GW | pLARmEB | 2HL_38325004 | 2H: 767379851 | -0.28 | 3.50 | 2.06 |
| BLUP_GW | pLARmEB | 3_212420276 | 3H: 492785054 | -0.36 | 5.17 | 3.50 |
| BLUP_GW | FASTmrEMMA | 3HL_39017959 | 3H: 491163483 | -0.86 | 4.36 | 4.39 |
| BLUP_GW | FASTmrMLM | 3HL_39017959 | 3H: 491163483 | -0.46 | 4.62 | 5.83 |
| BLUP_GW | ISIS EM-BLASSO | 3HL_39017959 | 3H: 491163483 | -0.58 | 7.55 | 9.08 |
| BLUP_GW | mrMLM | 3HL_39017959 | 3H: 491163483 | -0.50 | 4.42 | 6.79 |
| BLUP_GW | ISIS EM-BLASSO | 3HS_19331121 | 3H: 159555748 | 0.30 | 3.62 | 2.43 |
| BLUP_GW | ISIS EM-BLASSO | 4_3860802 | 4H: 410542 | -0.24 | 3.28 | 1.57 |
| BLUP_GW | pLARmEB | 4HS_8953586 | 4H: 8747991 | -0.25 | 3.35 | 1.73 |
| BLUP_GW | ISIS EM-BLASSO | 5_464770127 | 5H: 560736343 | -0.28 | 4.12 | 2.09 |
| BLUP_GW | ISIS EM-BLASSO | 5HL_34845177 | 5H: 278312622 | -0.24 | 3.30 | 1.23 |
| BLUP_GW | pLARmEB | 7_75697079 | 7H: 65973576 | 0.24 | 4.01 | 1.63 |
| BLUP_GW | ISIS EM-BLASSO | 7HS_24599779 | 7H: 332349563 | 0.74 | 5.00 | 14.41 |
| BLUP_GW | mrMLM | 7HS_24599779 | 7H: 332349563 | 0.84 | 4.67 | 18.64 |
| BLUP_GW | FASTmrMLM | M_149956_1482 | 2H: 663437677 | -0.33 | 3.34 | 2.56 |
| BLUP_GW | mrMLM | M_149956_1482 | 2H: 663437677 | -0.42 | 3.21 | 4.31 |
| BLUP_GW | pLARmEB | M_92618_573 | 4H: 497919156 | -0.26 | 4.30 | 1.85 |
| BLUP_GWS | pLARmEB | 2_522600068 | 2H: 649657420 | -0.02 | 3.71 | 3.29 |
| BLUP_GWS | pLARmEB | 3_529115904 | 3H: 654698488 | -0.01 | 3.55 | 1.32 |
| BLUP_GWS | FASTmrEMMA | 7_235393318 | 7H: 424178069 | -0.09 | 3.54 | 26.83 |
| BLUP_GWS | mrMLM | 7_235393318 | 7H: 424178069 | -0.06 | 3.77 | 42.25 |
| BLUP_GWS | pLARmEB | 7_235393318 | 7H: 424178069 | -0.06 | 7.83 | 34.11 |
| BLUP_GWS | pLARmEB | 7HL_12993590 | 7H: 619894777 | 0.00 | 3.04 | 0.00 |
| BLUP_GWS | FASTmrMLM | M_1593566_399 | 2H: 610649728 | -0.01 | 3.53 | 1.49 |
| BLUP_IL1 | pLARmEB | 1_81117585 | 1H: 250712497 | 0.72 | 8.95 | 5.36 |
| BLUP_IL1 | FASTmrMLM | 1H_12073861 | 1H: 552237356 | -0.35 | 3.01 | 1.25 |
| BLUP_IL1 | mrMLM | 1H_12073861 | 1H: 552237356 | -0.60 | 3.01 | 3.73 |
| BLUP_IL1 | pLARmEB | 1H_12073861 | 1H: 552237356 | -0.36 | 3.74 | 1.31 |
| BLUP_IL1 | FASTmrMLM | 2_399823529 | 2H: 521774247 | -0.59 | 5.44 | 3.71 |
| BLUP_IL1 | mrMLM | 2_399823529 | 2H: 521774247 | -0.75 | 5.44 | 5.96 |
| BLUP_IL1 | ISIS EM-BLASSO | 2_522610509 | 2H: 648821931 | 0.64 | 5.46 | 3.58 |
| BLUP_IL1 | pLARmEB | 2_522610509 | 2H: 648821931 | 0.81 | 9.92 | 5.77 |
| BLUP_IL1 | FASTmrEMMA | 2_524762464 | 2H: 649558019 | 1.26 | 4.55 | 3.34 |
| BLUP_IL1 | FASTmrMLM | 3_359178487 | 3H: 464607242 | -0.68 | 6.98 | 4.93 |
| BLUP_IL1 | mrMLM | 3_359178487 | 3H: 464607242 | -0.80 | 6.98 | 6.81 |
| BLUP_IL1 | ISIS EM-BLASSO | 3HL_25561608 | 2H: 704382692 | -0.53 | 3.90 | 2.86 |
| BLUP_IL1 | pLARmEB | 3HL_39017959 | 3H: 491163483 | -0.73 | 9.37 | 5.54 |
| BLUP_IL1 | ISIS EM-BLASSO | 6_451975519 | 6H: 492894732 | -0.94 | 6.76 | 9.19 |
| BLUP_IL1 | pLARmEB | 6HL_35290853 | 6H: 314043344 | -0.90 | 8.06 | 8.61 |
| BLUP_IL1 | FASTmrMLM | 6HL_40780311 | 6H: 513374629 | -1.21 | 16.29 | 15.71 |
| BLUP_IL1 | mrMLM | 6HL_40780311 | 6H: 513374629 | -1.32 | 16.29 | 18.50 |
| BLUP_IL1 | pLARmEB | 6HL_7541370 | 6H: 539712296 | -0.96 | 3.39 | 9.03 |
| BLUP_IL1 | FASTmrEMMA | 7_575487388 | 7H: 627311039 | 1.04 | 3.73 | 2.43 |
| BLUP_IL1 | FASTmrMLM | 7_575487388 | 7H: 627311039 | 0.63 | 5.75 | 3.83 |
| BLUP_IL1 | ISIS EM-BLASSO | 7_575487388 | 7H: 627311039 | 0.56 | 4.19 | 3.00 |
| BLUP_IL1 | mrMLM | 7_575487388 | 7H: 627311039 | 0.81 | 5.75 | 6.27 |
| BLUP_IL1 | pLARmEB | 7_575487388 | 7H: 627311039 | 0.60 | 7.81 | 3.46 |
| BLUP_IL1 | pLARmEB | 7HS_30257307 | 7H: 83769522 | 1.20 | 14.46 | 15.41 |
| BLUP_IL1 | FASTmrEMMA | 7HS_31244294 | 7H: 82552493 | 1.88 | 5.35 | 7.34 |
| BLUP_IL1 | FASTmrMLM | 7HS_31244294 | 7H: 82552493 | 0.85 | 6.01 | 7.72 |
| BLUP_IL1 | ISIS EM-BLASSO | 7HS_31244294 | 7H: 82552493 | 0.83 | 4.53 | 7.28 |
| BLUP_IL1 | mrMLM | 7HS_31244294 | 7H: 82552493 | 0.96 | 6.01 | 9.69 |
| BLUP_IL1 | FASTmrMLM | M_173442_1610 | 1H: 285199675 | 0.50 | 4.87 | 2.53 |
| BLUP_IL1 | mrMLM | M_173442_1610 | 1H: 285199675 | 0.68 | 4.87 | 4.69 |
| BLUP_IL1 | pLARmEB | M_271800_535 | 3H: 617544338 | -0.37 | 3.82 | 1.23 |
| BLUP_IL2 | FASTmrEMMA | 3_528771637 | 3H: 655521657 | -1.05 | 5.75 | 6.64 |
| BLUP_IL2 | pLARmEB | 3_528771637 | 3H: 655521657 | -0.49 | 6.60 | 6.32 |
| BLUP_IL2 | FASTmrMLM | 3HL_29995344 | 3H: 647162400 | -0.55 | 7.39 | 7.35 |
| BLUP_IL2 | mrMLM | 3HL_29995344 | 3H: 647162400 | -0.58 | 5.93 | 8.20 |
| BLUP_IL2 | FASTmrMLM | 4HL_18507097 | 4H: 501176373 | -0.29 | 4.15 | 2.87 |
| BLUP_IL2 | mrMLM | 4HL_18507097 | 4H: 501176373 | -0.37 | 3.01 | 4.50 |
| BLUP_IL2 | pLARmEB | 4HL_18507097 | 4H: 501176373 | -0.24 | 3.38 | 1.95 |
| BLUP_IL2 | FASTmrMLM | 7HS_23491202 | 7H: 311354310 | 0.78 | 4.34 | 19.43 |
| BLUP_IL2 | FASTmrMLM | M_114215_455 | 7H: 258071311 | -0.96 | 6.17 | 29.32 |
| BLUP_IL2 | mrMLM | M_114215_455 | 7H: 258071311 | -0.90 | 3.13 | 25.82 |
| BLUP_IL2 | pLARmEB | M_124067_1097 | 7H: 286599493 | -0.64 | 3.37 | 13.20 |
| BLUP_IL2 | pLARmEB | M_1604359_2677 | 3H: 423537442 | -0.28 | 4.64 | 2.63 |
| BLUP_IL3 | pLARmEB | 2_600474867 | 2H: 727925822 | -0.21 | 4.98 | 1.11 |
| BLUP_IL3 | pLARmEB | 2HL_46013510 | 2H: 460915213 | -0.13 | 3.45 | 0.49 |
| BLUP_IL3 | ISIS EM-BLASSO | 2HS_27419377 | 2H: 4578432 | 0.17 | 3.19 | 0.75 |
| BLUP_IL3 | pLARmEB | 2HS_27419377 | 2H: 4578432 | 0.13 | 3.81 | 0.49 |
| BLUP_IL3 | mrMLM | 3_511668322 | 3H: 636535362 | -1.01 | 18.49 | 20.81 |
| BLUP_IL3 | pLARmEB | 3_511668322 | 3H: 636535362 | -0.54 | 4.55 | 6.06 |
| BLUP_IL3 | ISIS EM-BLASSO | 3_511749149 | 3H: 633068955 | -0.48 | 3.01 | 4.88 |
| BLUP_IL3 | ISIS EM-BLASSO | 3HL_37004393 | 3H: 631870705 | -0.51 | 3.31 | 5.12 |
| BLUP_IL3 | pLARmEB | 3HL_37004393 | 3H: 631870705 | -0.51 | 6.34 | 5.03 |
| BLUP_IL3 | pLARmEB | 5_123999409 | 5H: 197561258 | -0.33 | 7.28 | 2.14 |
| BLUP_IL3 | pLARmEB | 5_292540539 | 5H: 455677160 | 0.28 | 6.70 | 1.72 |
| BLUP_IL3 | ISIS EM-BLASSO | 6_18118681 | 6H: 17542081 | 0.29 | 4.12 | 2.23 |
| BLUP_IL3 | pLARmEB | 6_464518834 | 6H: 512431137 | -0.17 | 4.47 | 0.75 |
| BLUP_IL4 | pLARmEB | 2_600474867 | 2H: 727925822 | -0.27 | 3.47 | 2.15 |
| BLUP_IL4 | FASTmrMLM | 2HS_32409186 | 3H: 651696476 | -0.78 | 11.13 | 15.32 |
| BLUP_IL4 | mrMLM | 2HS_32409186 | 3H: 651696476 | -0.79 | 9.61 | 15.89 |
| BLUP_IL4 | ISIS EM-BLASSO | 3_511668322 | 3H: 636535362 | -0.77 | 9.53 | 14.04 |
| BLUP_IL4 | pLARmEB | 3_511668322 | 3H: 636535362 | -0.82 | 14.07 | 15.97 |
| BLUP_IL4 | FASTmrMLM | 7HL_11281033 | 7H: 360793216 | -0.65 | 3.03 | 11.97 |
| BLUP_IL4 | pLARmEB | 7HL_11281033 | 7H: 360793216 | -0.46 | 3.14 | 6.10 |
| BLUP_IL4 | pLARmEB | 7HS_30003071 | 7H: 270546724 | -0.52 | 3.09 | 7.87 |
| BLUP_MSL | FASTmrEMMA | 1_35132055 | 1H: 20685614 | -0.57 | 3.97 | 2.53 |
| BLUP_MSL | ISIS EM-BLASSO | 1_35132055 | 1H: 20685614 | -0.21 | 3.21 | 1.49 |
| BLUP_MSL | mrMLM | 1_35132055 | 1H: 20685614 | -0.28 | 4.59 | 2.79 |
| BLUP_MSL | FASTmrMLM | 1H_81523248 | 1H: 370825902 | 0.00 | 3.17 | 0.00 |
| BLUP_MSL | FASTmrMLM | 2_447773331 | 2H: 560195592 | -0.29 | 4.28 | 2.92 |
| BLUP_MSL | mrMLM | 2_447773331 | 2H: 560195592 | -0.30 | 3.99 | 3.13 |
| BLUP_MSL | FASTmrMLM | 2_522610509 | 2H: 648821931 | 0.33 | 5.84 | 3.19 |
| BLUP_MSL | ISIS EM-BLASSO | 2_522610509 | 2H: 648821931 | 0.32 | 4.99 | 2.89 |
| BLUP_MSL | mrMLM | 2_522610509 | 2H: 648821931 | 0.36 | 5.14 | 3.67 |
| BLUP_MSL | pLARmEB | 2_585018981 | 2H: 707356497 | 0.24 | 6.76 | 1.75 |
| BLUP_MSL | pLARmEB | 2_600701693 | 2H: 728016483 | -0.70 | 8.72 | 16.70 |
| BLUP_MSL | FASTmrMLM | 2_600749073 | 2H: 727985438 | -0.67 | 21.60 | 14.75 |
| BLUP_MSL | ISIS EM-BLASSO | 2_600749073 | 2H: 727985438 | -0.74 | 22.36 | 18.16 |
| BLUP_MSL | mrMLM | 2_600749073 | 2H: 727985438 | -0.68 | 18.99 | 15.29 |
| BLUP_MSL | ISIS EM-BLASSO | 2HL_32158541 | 2H: 475151803 | -0.35 | 5.65 | 4.21 |
| BLUP_MSL | pLARmEB | 2HL_34260490 | 2H: 651436685 | 0.31 | 6.72 | 2.71 |
| BLUP_MSL | pLARmEB | 3HL_19292679 | 3H: 627356370 | -0.18 | 5.10 | 0.83 |
| BLUP_MSL | pLARmEB | 5HS_7015067 | 5H: 189047507 | -0.19 | 3.41 | 0.90 |
| BLUP_MSL | pLARmEB | 6_242226254 | 6H: 539665499 | 0.15 | 5.24 | 0.78 |
| BLUP_MSL | pLARmEB | 6_369171949 | 4H: 435633871 | -0.12 | 4.08 | 0.46 |
| BLUP_MSL | pLARmEB | 6HS_18979469 | 6H: 16292787 | 0.10 | 5.11 | 0.34 |
| BLUP_MSL | FASTmrMLM | 7_313370540 | 7H: 261177598 | 0.00 | 5.01 | 0.00 |
| BLUP_MSL | mrMLM | 7_313370540 | 7H: 261177598 | -0.78 | 3.77 | 20.77 |
| BLUP_MSL | pLARmEB | 7_313370540 | 7H: 261177598 | -0.72 | 6.09 | 17.39 |
| BLUP_MSL | pLARmEB | M_249593_1037 | 7H: 622802079 | 0.18 | 5.22 | 1.04 |
| BLUP_MSL | pLARmEB | M_257577_174 | 2H: 150648140 | -0.39 | 7.72 | 5.37 |
| BLUP_PH | pLARmEB | 1_12797074 | 1H: 20434535 | -0.80 | 5.97 | 0.41 |
| BLUP_PH | FASTmrEMMA | 1_463006138 | 1H: 554371992 | -2.88 | 5.19 | 1.02 |
| BLUP_PH | FASTmrMLM | 1_463006138 | 1H: 554371992 | -1.13 | 3.77 | 0.86 |
| BLUP_PH | mrMLM | 1_463006138 | 1H: 554371992 | -1.66 | 4.15 | 1.86 |
| BLUP_PH | pLARmEB | 1_463006138 | 1H: 554371992 | -1.12 | 7.64 | 0.83 |
| BLUP_PH | FASTmrMLM | 1H_34445125 | 6H: 506237108 | -1.47 | 6.69 | 1.44 |
| BLUP_PH | pLARmEB | 1H_34445125 | 6H: 506237108 | -1.85 | 15.31 | 2.27 |
| BLUP_PH | FASTmrEMMA | 2_447773331 | 2H: 560195592 | -3.84 | 3.51 | 2.13 |
| BLUP_PH | FASTmrMLM | 2_447773331 | 2H: 560195592 | -2.29 | 12.47 | 3.56 |
| BLUP_PH | mrMLM | 2_447773331 | 2H: 560195592 | -2.37 | 8.71 | 3.79 |
| BLUP_PH | pLARmEB | 2HS_27419377 | 2H: 4578432 | 0.87 | 5.27 | 0.52 |
| BLUP_PH | FASTmrEMMA | 2HS_32409186 | 3H: 651696476 | -7.15 | 14.97 | 6.86 |
| BLUP_PH | FASTmrMLM | 2HS_32409186 | 3H: 651696476 | -2.38 | 5.77 | 3.13 |
| BLUP_PH | ISIS EM-BLASSO | 2HS_32409186 | 3H: 651696476 | -3.68 | 12.39 | 7.48 |
| BLUP_PH | mrMLM | 2HS_32409186 | 3H: 651696476 | -3.70 | 16.72 | 7.58 |
| BLUP_PH | pLARmEB | 3_379673146 | 3H: 487356990 | -0.94 | 5.80 | 0.59 |
| BLUP_PH | FASTmrMLM | 3_504106156 | 3H: 626219628 | -1.45 | 4.54 | 1.04 |
| BLUP_PH | pLARmEB | 3_504106156 | 3H: 626219628 | -1.30 | 4.24 | 0.84 |
| BLUP_PH | pLARmEB | 3HL_37004393 | 3H: 631870705 | -2.07 | 6.63 | 2.15 |
| BLUP_PH | pLARmEB | 4_608194 | 4H: 195266358 | -1.74 | 10.62 | 2.09 |
| BLUP_PH | FASTmrMLM | 5_149952696 | 5H: 86005119 | -1.73 | 5.20 | 1.72 |
| BLUP_PH | pLARmEB | 5_149952696 | 5H: 86005119 | -1.30 | 6.46 | 0.96 |
| BLUP_PH | pLARmEB | 5HL_7623791 | 5H: 478872855 | 1.50 | 9.13 | 1.24 |
| BLUP_PH | FASTmrEMMA | 6_480485322 | 6H: 518364045 | -2.72 | 4.25 | 1.15 |
| BLUP_PH | mrMLM | 6_480485322 | 6H: 518364045 | -1.75 | 5.41 | 2.04 |
| BLUP_PH | ISIS EM-BLASSO | 6HL_6355520 | 6H: 516860898 | -1.52 | 4.13 | 1.57 |
| BLUP_PH | FASTmrEMMA | 7HS_12212266 | 7H: 81959684 | 3.14 | 4.07 | 1.39 |
| BLUP_PH | FASTmrMLM | 7HS_12212266 | 7H: 81959684 | 1.25 | 3.33 | 1.08 |
| BLUP_PH | pLARmEB | 7HS_12212266 | 7H: 81959684 | 1.59 | 9.13 | 1.73 |
| BLUP_PH | mrMLM | 7HS_33062962 | 7H: 108670637 | 2.01 | 3.72 | 2.75 |
| BLUP_PH | FASTmrMLM | M_1577838_3038 | 5H: 480225037 | 1.58 | 6.57 | 1.61 |
| BLUP_PH | ISIS EM-BLASSO | M_1999039_472 | 2H: 540094243 | -2.03 | 5.54 | 2.83 |
| BLUP_PH | pLARmEB | M_1999039_472 | 2H: 540094243 | -2.44 | 16.21 | 4.11 |
| BLUP_PH | pLARmEB | M_2038491_176 | 4H: 23190930 | -1.43 | 7.85 | 1.28 |
| BLUP_PH | FASTmrMLM | M_249593_1037 | 7H: 622802079 | 1.07 | 5.39 | 0.69 |
| BLUP_PH | mrMLM | M_249593_1037 | 7H: 622802079 | 1.53 | 3.61 | 1.43 |
| BLUP_PH | pLARmEB | M_249593_1037 | 7H: 622802079 | 1.42 | 10.17 | 1.22 |
| BLUP_SLP | ISIS EM-BLASSO | 2_253685642 | 2H: 518515964 | -32.11 | 8.56 | 5.64 |
| BLUP_SLP | pLARmEB | 2_253685642 | 2H: 518515964 | -34.30 | 10.57 | 5.96 |
| BLUP_SLP | mrMLM | 2_522610509 | 2H: 648821931 | 37.34 | 4.04 | 4.79 |
| BLUP_SLP | FASTmrMLM | 4_16553551 | 4H: 15498372 | -24.57 | 5.29 | 3.22 |
| BLUP_SLP | ISIS EM-BLASSO | M_1605646_794 | 4H: 16761959 | -27.85 | 8.06 | 4.14 |
| BLUP_SLP | mrMLM | M_1605646_794 | 4H: 16761959 | -28.24 | 6.81 | 3.26 |
| BLUP_SLP | pLARmEB | M_1605646_794 | 4H: 16761959 | -28.40 | 9.76 | 3.99 |
| BLUP_SLP | FASTmrEMMA | vrs1 | 2H: 652030802 | -245.26 | 38.81 | 68.78 |
| BLUP_SLP | FASTmrMLM | vrs1 | 2H: 652030802 | -119.45 | 41.30 | 65.26 |
| BLUP_SLP | ISIS EM-BLASSO | vrs1 | 2H: 652030802 | -131.34 | 51.41 | 77.51 |
| BLUP_SLP | mrMLM | vrs1 | 2H: 652030802 | -152.87 | 4.66 | 80.30 |
| BLUP_SLP | pLARmEB | vrs1 | 2H: 652030802 | -119.45 | 27.21 | 59.40 |
| BLUP_SMS | pLARmEB | 1H_40034223 | 1H: 379423220 | 1.88 | 8.56 | 0.89 |
| BLUP_SMS | ISIS EM-BLASSO | 1H_68122776 | 1H: 387428286 | 2.11 | 8.35 | 1.02 |
| BLUP_SMS | ISIS EM-BLASSO | 2_406934594 | 2H: 535680815 | -4.41 | 3.63 | 4.40 |
| BLUP_SMS | pLARmEB | 2_406934594 | 2H: 535680815 | -3.57 | 8.46 | 3.14 |
| BLUP_SMS | mrMLM | 2_527636020 | 2H: 651399477 | -2.88 | 4.30 | 1.77 |
| BLUP_SMS | mrMLM | 2_530198498 | 2H: 659234271 | 2.09 | 4.54 | 0.96 |
| BLUP_SMS | pLARmEB | 2_555061031 | 2H: 686654327 | -1.43 | 3.78 | 0.45 |
| BLUP_SMS | pLARmEB | 2_588847616 | 2H: 712636517 | 1.22 | 4.18 | 0.35 |
| BLUP_SMS | ISIS EM-BLASSO | 4_20853756 | 4H: 19147541 | -1.91 | 6.99 | 0.79 |
| BLUP_SMS | pLARmEB | 4_26092518 | 4H: 26418897 | -1.63 | 7.74 | 0.64 |
| BLUP_SMS | pLARmEB | 5HL_40553345 | 2H: 578521457 | -1.29 | 3.07 | 0.42 |
| BLUP_SMS | pLARmEB | 6HL_18597185 | 6H: 548685973 | 1.15 | 4.22 | 0.31 |
| BLUP_SMS | pLARmEB | 6HL_7408863 | 6H: 542499733 | -1.52 | 5.33 | 0.55 |
| BLUP_SMS | pLARmEB | M_2571394_329 | 4H: 205055735 | -2.94 | 5.27 | 1.68 |
| BLUP_SMS | FASTmrEMMA | vrs1 | 2H: 652030802 | -36.56 | 29.32 | 74.96 |
| BLUP_SMS | FASTmrMLM | vrs1 | 2H: 652030802 | -18.15 | 29.32 | 73.88 |
| BLUP_SMS | ISIS EM-BLASSO | vrs1 | 2H: 652030802 | -21.61 | 82.16 | 90.03 |
| BLUP_SMS | mrMLM | vrs1 | 2H: 652030802 | -19.10 | 13.63 | 81.82 |
| BLUP_SMS | pLARmEB | vrs1 | 2H: 652030802 | -18.59 | 48.68 | 72.30 |
| BLUP_SP | FASTmrMLM | 2_522600068 | 2H: 649657420 | 0.60 | 17.52 | 36.37 |
| BLUP_SP | ISIS EM-BLASSO | 2_522600068 | 2H: 649657420 | 0.60 | 16.53 | 36.31 |
| BLUP_SP | mrMLM | 2_522600068 | 2H: 649657420 | 0.63 | 16.09 | 39.25 |
| BLUP_SP | FASTmrEMMA | 2_522610509 | 2H: 648821931 | 1.14 | 16.22 | 31.98 |
| BLUP_SP | FASTmrEMMA | 4HS_10254166 | 4H: 22549836 | -0.44 | 4.63 | 4.95 |
| BLUP_SP | FASTmrMLM | 4HS_10254166 | 4H: 22549836 | -0.18 | 3.45 | 3.78 |
| BLUP_SP | mrMLM | 4HS_10254166 | 4H: 22549836 | -0.24 | 3.30 | 6.60 |
| BLUP_SP | pLARmEB | 4HS_10254166 | 4H: 22549836 | -0.19 | 3.03 | 4.32 |
| BLUP_TGW | pLARmEB | 1_288679554 | 1H: 389944422 | -0.87 | 4.72 | 1.23 |
| BLUP_TGW | ISIS EM-BLASSO | 3_434739769 | 3H: 548414321 | 1.36 | 5.10 | 3.24 |
| BLUP_TGW | FASTmrMLM | 3HS_23539468 | 3H: 272283784 | -1.31 | 5.83 | 2.68 |
| BLUP_TGW | mrMLM | 3HS_23539468 | 3H: 272283784 | -1.67 | 5.98 | 4.39 |
| BLUP_TGW | pLARmEB | 3HS_23539468 | 3H: 272283784 | -1.18 | 5.97 | 2.19 |
| BLUP_TGW | FASTmrMLM | 4_121425641 | 4H: 78265040 | -1.38 | 6.71 | 3.23 |
| BLUP_TGW | mrMLM | 4_121425641 | 4H: 78265040 | -1.55 | 6.67 | 4.08 |
| BLUP_TGW | pLARmEB | 4HS_39123780 | 4H: 351726574 | -1.67 | 9.16 | 4.95 |
| BLUP_TGW | mrMLM | 5HL_16887912 | 5H: 416800216 | 1.22 | 3.19 | 1.82 |
| BLUP_TGW | FASTmrMLM | 5HL_49328035 | 5H: 521373280 | 0.69 | 3.05 | 0.83 |
| BLUP_TGW | FASTmrMLM | 7HS_10887541 | 7H: 72344563 | 1.57 | 6.27 | 4.39 |
| BLUP_TGW | mrMLM | 7HS_10887541 | 7H: 72344563 | 1.95 | 7.25 | 6.77 |
| BLUP_TGW | FASTmrEMMA | 7HS_22228446 | 7H: 81968748 | 3.20 | 3.44 | 3.98 |
| BLUP_TGW | pLARmEB | 7HS_22228446 | 7H: 81968748 | 1.88 | 8.13 | 6.23 |
| BLUP_TGW | FASTmrMLM | 7HS_7388828 | 7H: 324619556 | 1.68 | 3.02 | 4.66 |
| BLUP_TGW | FASTmrEMMA | vrs1 | 2H: 652030802 | 12.32 | 7.39 | 55.32 |
| BLUP_TGW | FASTmrMLM | vrs1 | 2H: 652030802 | 5.96 | 42.66 | 51.76 |
| BLUP_TGW | ISIS EM-BLASSO | vrs1 | 2H: 652030802 | 6.01 | 36.78 | 52.55 |
| BLUP_TGW | mrMLM | vrs1 | 2H: 652030802 | 5.84 | 11.89 | 49.60 |
| BLUP_TGW | pLARmEB | vrs1 | 2H: 652030802 | 5.84 | 46.32 | 49.72 |

^a^ Trait ID was defined as “year+ abbreviation trait name”

**Table S3. The significant associations between SNP markers and 14 agronomic traits repeatedly detected in multiple environments and GWAS methods.**

| Trait ID ^a^ | Method | Markers | Physic position (bp) | QTN effect | LOD score | r^2^ (%) |
| --- | --- | --- | --- | --- | --- | --- |
| 2010_PH | FASTmrEMMA | 1_463006138 | 1H: 554371992 | -2.56 | 3.39 | 0.63 |
| 2010_PH | pLARmEB | 1_463006138 | 1H: 554371992 | -1.44 | 4.21 | 1.09 |
| 2012_PH | mrMLM | 1_463006138 | 1H: 554371992 | -2.59 | 6.64 | 4.07 |
| 2012_PH | FASTmrMLM | 1_463006138 | 1H: 554371992 | -1.96 | 5.32 | 2.32 |
| 2012_PH | FASTmrEMMA | 1_463006138 | 1H: 554371992 | -3.82 | 4.48 | 1.62 |
| 2012_PH | pLARmEB | 1_463006138 | 1H: 554371992 | -1.96 | 5.23 | 2.33 |
| 2012_PH | ISIS EM-BLASSO | 1_463006138 | 1H: 554371992 | -1.64 | 4.31 | 1.62 |
| BLUP_PH | FASTmrEMMA | 1_463006138 | 1H: 554371992 | -2.88 | 5.19 | 1.02 |
| BLUP_PH | FASTmrMLM | 1_463006138 | 1H: 554371992 | -1.13 | 3.77 | 0.86 |
| BLUP_PH | mrMLM | 1_463006138 | 1H: 554371992 | -1.66 | 4.15 | 1.86 |
| BLUP_PH | pLARmEB | 1_463006138 | 1H: 554371992 | -1.12 | 7.64 | 0.83 |
| 2010_PH | mrMLM | M_1999039_472 | 2H: 540094243 | -2.93 | 8.14 | 4.64 |
| 2010_PH | FASTmrMLM | M_1999039_472 | 2H: 540094243 | -2.61 | 8.50 | 3.69 |
| 2010_PH | ISIS EM-BLASSO | M_1999039_472 | 2H: 540094243 | -2.44 | 6.38 | 3.22 |
| BLUP_PH | ISIS EM-BLASSO | M_1999039_472 | 2H: 540094243 | -2.03 | 5.54 | 2.83 |
| BLUP_PH | pLARmEB | M_1999039_472 | 2H: 540094243 | -2.44 | 16.21 | 4.11 |
| 2010_PH | FASTmrEMMA | 2_447773331 | 2H: 560195592 | -5.01 | 8.57 | 2.85 |
| 2010_PH | pLARmEB | 2_447773331 | 2H: 560195592 | -2.63 | 10.42 | 3.69 |
| BLUP_PH | FASTmrEMMA | 2_447773331 | 2H: 560195592 | -3.84 | 3.51 | 2.13 |
| BLUP_PH | FASTmrMLM | 2_447773331 | 2H: 560195592 | -2.29 | 12.47 | 3.56 |
| BLUP_PH | mrMLM | 2_447773331 | 2H: 560195592 | -2.37 | 8.71 | 3.79 |
| 2009_PH | mrMLM | M_1663886_573 | 2H: 564116957 | -2.07 | 4.28 | 3.22 |
| 2009_PH | FASTmrMLM | M_1663886_573 | 2H: 564116957 | -1.87 | 6.39 | 2.61 |
| 2009_PH | pLARmEB | M_1663886_573 | 2H: 564116957 | -1.78 | 5.66 | 2.37 |
| 2009_PH | ISIS EM-BLASSO | M_1663886_573 | 2H: 564116957 | -1.84 | 4.91 | 2.52 |
| 2009_PH | pLARmEB | 3HL_37004393 | 3H: 631870705 | -2.96 | 9.78 | 4.75 |
| BLUP_PH | pLARmEB | 3HL_37004393 | 3H: 631870705 | -2.07 | 6.63 | 2.15 |
| 2010_PH | mrMLM | 3_511749149 | 3H: 633068955 | -3.95 | 13.79 | 6.53 |
| 2010_PH | FASTmrMLM | 3_511749149 | 3H: 633068955 | -3.89 | 13.70 | 6.35 |
| 2010_PH | pLARmEB | 3_511749149 | 3H: 633068955 | -3.97 | 15.96 | 6.59 |
| 2012_PH | mrMLM | 3_511749149 | 3H: 633068955 | -3.87 | 3.38 | 7.23 |
| 2009_PH | mrMLM | 2HS_32409186 | 3H: 651696476 | -3.34 | 9.77 | 6.73 |
| 2009_PH | FASTmrMLM | 2HS_32409186 | 3H: 651696476 | -3.25 | 9.95 | 6.38 |
| 2009_PH | FASTmrEMMA | 2HS_32409186 | 3H: 651696476 | -7.45 | 9.66 | 8.12 |
| 2009_PH | ISIS EM-BLASSO | 2HS_32409186 | 3H: 651696476 | -3.46 | 12.23 | 7.23 |
| 2010_PH | ISIS EM-BLASSO | 2HS_32409186 | 3H: 651696476 | -2.90 | 4.18 | 3.66 |
| 2012_PH | FASTmrEMMA | 2HS_32409186 | 3H: 651696476 | -7.63 | 11.12 | 7.07 |
| 2012_PH | ISIS EM-BLASSO | 2HS_32409186 | 3H: 651696476 | -4.25 | 14.09 | 9.03 |
| BLUP_PH | FASTmrEMMA | 2HS_32409186 | 3H: 651696476 | -7.15 | 14.97 | 6.86 |
| BLUP_PH | FASTmrMLM | 2HS_32409186 | 3H: 651696476 | -2.38 | 5.77 | 3.13 |
| BLUP_PH | ISIS EM-BLASSO | 2HS_32409186 | 3H: 651696476 | -3.68 | 12.39 | 7.48 |
| BLUP_PH | mrMLM | 2HS_32409186 | 3H: 651696476 | -3.70 | 16.72 | 7.58 |
| 2009_PH | pLARmEB | 7HS_12212266 | 7H: 81959684 | 1.79 | 4.78 | 2.40 |
| 2009_PH | ISIS EM-BLASSO | 7HS_12212266 | 7H: 81959684 | 1.78 | 4.10 | 2.36 |
| BLUP_PH | FASTmrEMMA | 7HS_12212266 | 7H: 81959684 | 3.14 | 4.07 | 1.39 |
| BLUP_PH | FASTmrMLM | 7HS_12212266 | 7H: 81959684 | 1.25 | 3.33 | 1.08 |
| BLUP_PH | pLARmEB | 7HS_12212266 | 7H: 81959684 | 1.59 | 9.13 | 1.73 |
| 2009_PH | mrMLM | 7HS_33062962 | 7H: 108670637 | 2.86 | 4.82 | 6.07 |
| 2009_PH | FASTmrMLM | 7HS_33062962 | 7H: 108670637 | 2.62 | 6.60 | 5.11 |
| 2010_PH | mrMLM | 7HS_33062962 | 7H: 108670637 | 2.92 | 4.92 | 4.57 |
| 2010_PH | FASTmrMLM | 7HS_33062962 | 7H: 108670637 | 2.55 | 5.35 | 3.49 |
| 2010_PH | FASTmrEMMA | 7HS_33062962 | 7H: 108670637 | 4.53 | 4.31 | 2.31 |
| 2010_PH | pLARmEB | 7HS_33062962 | 7H: 108670637 | 2.23 | 4.58 | 2.67 |
| BLUP_PH | mrMLM | 7HS_33062962 | 7H: 108670637 | 2.01 | 3.72 | 2.75 |
| 2012_PH | mrMLM | M_249593_1037 | 7H: 622802079 | 2.21 | 5.11 | 2.68 |
| 2012_PH | FASTmrMLM | M_249593_1037 | 7H: 622802079 | 1.33 | 3.38 | 0.98 |
| BLUP_PH | FASTmrMLM | M_249593_1037 | 7H: 622802079 | 1.07 | 5.39 | 0.69 |
| BLUP_PH | mrMLM | M_249593_1037 | 7H: 622802079 | 1.53 | 3.61 | 1.43 |
| BLUP_PH | pLARmEB | M_249593_1037 | 7H: 622802079 | 1.42 | 10.17 | 1.22 |
| 2012_IL1 | mrMLM | M_173442_1610 | 1H: 285199675 | 0.78 | 4.31 | 3.10 |
| 2012_IL1 | FASTmrMLM | M_173442_1610 | 1H: 285199675 | 0.62 | 5.02 | 2.27 |
| 2012_IL1 | pLARmEB | M_173442_1610 | 1H: 285199675 | 0.58 | 4.98 | 2.20 |
| BLUP_IL1 | FASTmrMLM | M_173442_1610 | 1H: 285199675 | 0.50 | 4.87 | 2.53 |
| BLUP_IL1 | mrMLM | M_173442_1610 | 1H: 285199675 | 0.68 | 4.87 | 4.69 |
| 2012_IL1 | mrMLM | 2_399823529 | 2H: 521774247 | -0.77 | 4.49 | 3.12 |
| 2012_IL1 | FASTmrMLM | 2_399823529 | 2H: 521774247 | -0.61 | 5.03 | 2.26 |
| 2012_IL1 | FASTmrEMMA | 2_399823529 | 2H: 521774247 | -1.31 | 3.23 | 2.38 |
| BLUP_IL1 | FASTmrMLM | 2_399823529 | 2H: 521774247 | -0.59 | 5.44 | 3.71 |
| BLUP_IL1 | mrMLM | 2_399823529 | 2H: 521774247 | -0.75 | 5.44 | 5.96 |
| 2012_IL1 | mrMLM | 7HS_21829337 | 7H: 81889341 | 1.22 | 7.38 | 7.93 |
| 2012_IL1 | FASTmrMLM | 7HS_21829337 | 7H: 81889341 | 1.14 | 7.28 | 7.85 |
| 2012_IL1 | pLARmEB | 7HS_21829337 | 7H: 81889341 | 1.33 | 10.87 | 11.75 |
| 2010_IL1 | mrMLM | 7HS_12212266 | 7H: 81959684 | 1.65 | 12.01 | 22.62 |
| 2010_IL1 | FASTmrMLM | 7HS_12212266 | 7H: 81959684 | 1.56 | 12.10 | 20.22 |
| 2010_IL1 | FASTmrEMMA | 7HS_12212266 | 7H: 81959684 | 3.32 | 11.18 | 19.04 |
| 2010_IL1 | pLARmEB | 7HS_12212266 | 7H: 81959684 | 1.52 | 11.81 | 19.29 |
| 2010_IL1 | ISIS EM-BLASSO | 7HS_12212266 | 7H: 81959684 | 1.32 | 9.53 | 14.60 |
| 2012_IL1 | FASTmrEMMA | 7HS_31244294 | 7H: 82552493 | 2.06 | 3.82 | 5.61 |
| BLUP_IL1 | FASTmrEMMA | 7HS_31244294 | 7H: 82552493 | 1.88 | 5.35 | 7.34 |
| BLUP_IL1 | FASTmrMLM | 7HS_31244294 | 7H: 82552493 | 0.85 | 6.01 | 7.72 |
| BLUP_IL1 | ISIS EM-BLASSO | 7HS_31244294 | 7H: 82552493 | 0.83 | 4.53 | 7.28 |
| BLUP_IL1 | mrMLM | 7HS_31244294 | 7H: 82552493 | 0.96 | 6.01 | 9.69 |
| 2009_IL1 | pLARmEB | 7HS_30257307 | 7H: 83769522 | 0.62 | 3.21 | 3.53 |
| BLUP_IL1 | pLARmEB | 7HS_30257307 | 7H: 83769522 | 1.20 | 14.46 | 15.41 |
| 2009_IL1 | FASTmrMLM | 7_95992736 | 7H: 84350472 | 0.87 | 4.56 | 7.14 |
| 2009_IL1 | FASTmrEMMA | 7_95992736 | 7H: 84350472 | 2.08 | 3.56 | 8.43 |
| 2009_IL1 | ISIS EM-BLASSO | 7_95992736 | 7H: 84350472 | 0.79 | 3.78 | 5.82 |
| 2012_IL1 | mrMLM | 7_575487388 | 7H: 627311039 | 0.80 | 4.70 | 3.10 |
| 2012_IL1 | FASTmrMLM | 7_575487388 | 7H: 627311039 | 0.63 | 4.96 | 2.18 |
| 2012_IL1 | pLARmEB | 7_575487388 | 7H: 627311039 | 0.87 | 7.04 | 4.64 |
| 2012_IL1 | ISIS EM-BLASSO | 7_575487388 | 7H: 627311039 | 0.51 | 3.11 | 1.60 |
| BLUP_IL1 | FASTmrEMMA | 7_575487388 | 7H: 627311039 | 1.04 | 3.73 | 2.43 |
| BLUP_IL1 | FASTmrMLM | 7_575487388 | 7H: 627311039 | 0.63 | 5.75 | 3.83 |
| BLUP_IL1 | ISIS EM-BLASSO | 7_575487388 | 7H: 627311039 | 0.56 | 4.19 | 3.00 |
| BLUP_IL1 | mrMLM | 7_575487388 | 7H: 627311039 | 0.81 | 5.75 | 6.27 |
| BLUP_IL1 | pLARmEB | 7_575487388 | 7H: 627311039 | 0.60 | 7.81 | 3.46 |
| 2012_IL2 | mrMLM | M_114215_455 | 7H: 258071311 | -5.24 | 7.27 | 55.77 |
| 2012_IL2 | FASTmrMLM | M_114215_455 | 7H: 258071311 | -3.83 | 6.80 | 51.34 |
| 2012_IL2 | FASTmrEMMA | M_114215_455 | 7H: 258071311 | -7.66 | 5.16 | 49.41 |
| BLUP_IL2 | FASTmrMLM | M_114215_455 | 7H: 258071311 | -0.96 | 6.17 | 29.32 |
| BLUP_IL2 | mrMLM | M_114215_455 | 7H: 258071311 | -0.90 | 3.13 | 25.82 |
| 2009_IL3 | mrMLM | 3HL_37004393 | 3H: 631870705 | -0.78 | 12.83 | 13.67 |
| 2009_IL3 | FASTmrMLM | 3HL_37004393 | 3H: 631870705 | -0.76 | 12.94 | 12.87 |
| 2009_IL3 | pLARmEB | 3HL_37004393 | 3H: 631870705 | -0.79 | 18.15 | 13.85 |
| BLUP_IL3 | ISIS EM-BLASSO | 3HL_37004393 | 3H: 631870705 | -0.51 | 3.31 | 5.12 |
| BLUP_IL3 | pLARmEB | 3HL_37004393 | 3H: 631870705 | -0.51 | 6.34 | 5.03 |
| 2012_IL3 | mrMLM | 3_511749149 | 3H: 633068955 | -1.12 | 12.68 | 16.08 |
| 2012_IL3 | FASTmrMLM | 3_511749149 | 3H: 633068955 | -1.09 | 4.66 | 15.21 |
| BLUP_IL3 | ISIS EM-BLASSO | 3_511749149 | 3H: 633068955 | -0.48 | 3.01 | 4.88 |
| 2010_IL3 | FASTmrEMMA | 3_511668322 | 3H: 636535362 | -2.30 | 17.94 | 19.23 |
| 2010_IL3 | pLARmEB | 3_511668322 | 3H: 636535362 | -0.96 | 6.53 | 13.65 |
| 2010_IL3 | ISIS EM-BLASSO | 3_511668322 | 3H: 636535362 | -1.21 | 21.75 | 21.70 |
| 2012_IL3 | pLARmEB | 3_511668322 | 3H: 636535362 | -1.20 | 13.60 | 17.89 |
| BLUP_IL3 | mrMLM | 3_511668322 | 3H: 636535362 | -1.01 | 18.49 | 20.81 |
| BLUP_IL3 | pLARmEB | 3_511668322 | 3H: 636535362 | -0.54 | 4.55 | 6.06 |
| 2009_IL3 | mrMLM | 6_14536026 | 6H: 16165407 | 0.33 | 3.27 | 3.27 |
| 2009_IL3 | FASTmrMLM | 6_14536026 | 6H: 16165407 | 0.25 | 4.13 | 1.89 |
| 2009_IL3 | FASTmrEMMA | 6HS_18979469 | 6H: 16292787 | 0.65 | 4.78 | 2.66 |
| 2009_IL3 | pLARmEB | 6HS_18979469 | 6H: 16292787 | 0.35 | 6.71 | 3.68 |
| 2009_IL3 | ISIS EM-BLASSO | 6_18118681 | 6H: 17542081 | 0.34 | 5.72 | 3.34 |
| 2012_IL3 | ISIS EM-BLASSO | 6_18118681 | 6H: 17542081 | 0.37 | 3.15 | 2.17 |
| BLUP_IL3 | ISIS EM-BLASSO | 6_18118681 | 6H: 17542081 | 0.29 | 4.12 | 2.23 |
| 2009_IL4 | mrMLM | M_1778358_754 | 2H: 4629895 | 0.48 | 4.71 | 6.02 |
| 2009_IL4 | FASTmrMLM | M_1778358_754 | 2H: 4629895 | 0.40 | 4.48 | 4.27 |
| 2009_IL4 | pLARmEB | M_1778358_754 | 2H: 4629895 | 0.36 | 3.89 | 3.47 |
| 2009_IL4 | ISIS EM-BLASSO | M_1778358_754 | 2H: 4629895 | 0.35 | 3.58 | 3.26 |
| 2010_IL4 | mrMLM | 2_4900503 | 2H: 4950022 | 0.39 | 4.06 | 2.81 |
| 2010_IL4 | FASTmrMLM | 2_4900503 | 2H: 4950022 | 0.34 | 4.57 | 2.17 |
| 2010_IL4 | FASTmrEMMA | 2_4900503 | 2H: 4950022 | 0.67 | 3.93 | 1.79 |
| 2010_IL4 | pLARmEB | 2_4900503 | 2H: 4950022 | 0.35 | 5.09 | 2.23 |
| 2010_IL4 | ISIS EM-BLASSO | 2_4900503 | 2H: 4950022 | 0.36 | 4.58 | 2.47 |
| 2010_IL4 | mrMLM | 3HL_37004393 | 3H: 631870705 | -0.84 | 12.18 | 9.57 |
| 2010_IL4 | FASTmrMLM | 3HL_37004393 | 3H: 631870705 | -0.80 | 11.42 | 8.75 |
| 2010_IL4 | ISIS EM-BLASSO | 3HL_37004393 | 3H: 631870705 | -0.76 | 10.09 | 7.88 |
| 2009_IL4 | mrMLM | 3_511071233 | 3H: 635159926 | -0.80 | 10.22 | 12.48 |
| 2009_IL4 | FASTmrMLM | 3_511071233 | 3H: 635159926 | -0.77 | 5.69 | 11.59 |
| 2009_IL4 | pLARmEB | 3_511071233 | 3H: 635159926 | -0.79 | 9.75 | 12.09 |
| 2009_IL4 | ISIS EM-BLASSO | 3_511071233 | 3H: 635159926 | -0.68 | 7.53 | 8.87 |
| 2012_IL4 | FASTmrMLM | 3_511668322 | 3H: 636535362 | -1.07 | 6.37 | 5.73 |
| BLUP_IL4 | ISIS EM-BLASSO | 3_511668322 | 3H: 636535362 | -0.77 | 9.53 | 14.04 |
| BLUP_IL4 | pLARmEB | 3_511668322 | 3H: 636535362 | -0.82 | 14.07 | 15.97 |
| 2012_IL4 | FASTmrEMMA | 7HL_11281033 | 7H: 360793216 | -3.19 | 3.00 | 18.98 |
| 2012_IL4 | pLARmEB | 7HL_11281033 | 7H: 360793216 | -1.59 | 3.46 | 19.57 |
| BLUP_IL4 | FASTmrMLM | 7HL_11281033 | 7H: 360793216 | -0.65 | 3.03 | 11.97 |
| BLUP_IL4 | pLARmEB | 7HL_11281033 | 7H: 360793216 | -0.46 | 3.14 | 6.10 |
| 2010_MSL | mrMLM | 1_35132055 | 1H: 20685614 | -0.34 | 6.62 | 3.47 |
| 2010_MSL | FASTmrMLM | 1_35132055 | 1H: 20685614 | -0.31 | 6.61 | 2.91 |
| 2010_MSL | FASTmrEMMA | 1_35132055 | 1H: 20685614 | -0.66 | 4.95 | 2.85 |
| 2010_MSL | pLARmEB | 1_35132055 | 1H: 20685614 | -0.22 | 5.92 | 1.50 |
| BLUP_MSL | FASTmrEMMA | 1_35132055 | 1H: 20685614 | -0.57 | 3.97 | 2.53 |
| BLUP_MSL | ISIS EM-BLASSO | 1_35132055 | 1H: 20685614 | -0.21 | 3.21 | 1.49 |
| BLUP_MSL | mrMLM | 1_35132055 | 1H: 20685614 | -0.28 | 4.59 | 2.79 |
| 2010_MSL | mrMLM | 2_447773331 | 2H: 560195592 | -0.46 | 5.29 | 6.35 |
| 2010_MSL | FASTmrMLM | 2_447773331 | 2H: 560195592 | -0.34 | 5.75 | 3.46 |
| 2010_MSL | FASTmrEMMA | 2_447773331 | 2H: 560195592 | -0.90 | 8.37 | 5.08 |
| BLUP_MSL | FASTmrMLM | 2_447773331 | 2H: 560195592 | -0.29 | 4.28 | 2.92 |
| BLUP_MSL | mrMLM | 2_447773331 | 2H: 560195592 | -0.30 | 3.99 | 3.13 |
| 2010_MSL | pLARmEB | 2_522610509 | 2H: 648821931 | 0.31 | 6.68 | 2.31 |
| 2012_MSL | pLARmEB | 2_522610509 | 2H: 648821931 | 0.27 | 3.61 | 1.57 |
| BLUP_MSL | FASTmrMLM | 2_522610509 | 2H: 648821931 | 0.33 | 5.84 | 3.19 |
| BLUP_MSL | ISIS EM-BLASSO | 2_522610509 | 2H: 648821931 | 0.32 | 4.99 | 2.89 |
| BLUP_MSL | mrMLM | 2_522610509 | 2H: 648821931 | 0.36 | 5.14 | 3.67 |
| 2009_MSL | mrMLM | 2HL_34260490 | 2H: 651436685 | 0.40 | 7.85 | 4.51 |
| 2009_MSL | FASTmrMLM | 2HL_34260490 | 2H: 651436685 | 0.38 | 7.47 | 4.02 |
| 2009_MSL | pLARmEB | 2HL_34260490 | 2H: 651436685 | 0.41 | 10.16 | 4.65 |
| 2009_MSL | ISIS EM-BLASSO | 2HL_34260490 | 2H: 651436685 | 0.39 | 6.46 | 4.31 |
| BLUP_MSL | pLARmEB | 2HL_34260490 | 2H: 651436685 | 0.31 | 6.72 | 2.71 |
| 2009_MSL | mrMLM | 2_600749073 | 2H: 727985438 | -0.52 | 14.46 | 9.02 |
| 2009_MSL | FASTmrMLM | 2_600749073 | 2H: 727985438 | -0.50 | 15.62 | 8.43 |
| 2009_MSL | FASTmrEMMA | 2_600749073 | 2H: 727985438 | -1.09 | 11.89 | 8.80 |
| 2009_MSL | pLARmEB | 2_600749073 | 2H: 727985438 | -0.25 | 3.82 | 2.09 |
| 2009_MSL | ISIS EM-BLASSO | 2_600749073 | 2H: 727985438 | -0.46 | 9.88 | 7.10 |
| 2010_MSL | mrMLM | 2_600749073 | 2H: 727985438 | -0.74 | 22.85 | 15.54 |
| 2010_MSL | FASTmrMLM | 2_600749073 | 2H: 727985438 | -0.75 | 22.92 | 16.02 |
| 2010_MSL | FASTmrEMMA | 2_600749073 | 2H: 727985438 | -1.49 | 17.77 | 14.04 |
| 2010_MSL | pLARmEB | 2_600749073 | 2H: 727985438 | -0.45 | 3.29 | 5.80 |
| 2010_MSL | ISIS EM-BLASSO | 2_600749073 | 2H: 727985438 | -0.51 | 4.37 | 7.27 |
| 2012_MSL | mrMLM | 2_600749073 | 2H: 727985438 | -0.73 | 13.09 | 15.01 |
| 2012_MSL | FASTmrMLM | 2_600749073 | 2H: 727985438 | -0.72 | 12.72 | 14.49 |
| 2012_MSL | ISIS EM-BLASSO | 2_600749073 | 2H: 727985438 | -0.89 | 17.33 | 22.38 |
| BLUP_MSL | FASTmrMLM | 2_600749073 | 2H: 727985438 | -0.67 | 21.60 | 14.75 |
| BLUP_MSL | ISIS EM-BLASSO | 2_600749073 | 2H: 727985438 | -0.74 | 22.36 | 18.16 |
| BLUP_MSL | mrMLM | 2_600749073 | 2H: 727985438 | -0.68 | 18.99 | 15.29 |
| 2009_SMS | pLARmEB | 2_406934594 | 2H: 535680815 | -4.58 | 12.96 | 5.39 |
| 2010_SMS | ISIS EM-BLASSO | 2_406934594 | 2H: 535680815 | -4.20 | 5.68 | 3.91 |
| BLUP_SMS | ISIS EM-BLASSO | 2_406934594 | 2H: 535680815 | -4.41 | 3.63 | 4.40 |
| BLUP_SMS | pLARmEB | 2_406934594 | 2H: 535680815 | -3.57 | 8.46 | 3.14 |
| 2009_SMS | mrMLM | Vrs1 | 2H: 652030802 | -16.39 | 6.24 | 66.32 |
| 2009_SMS | FASTmrMLM | Vrs1 | 2H: 652030802 | -18.01 | 49.57 | 80.08 |
| 2009_SMS | FASTmrEMMA | Vrs1 | 2H: 652030802 | -36.05 | 19.47 | 80.21 |
| 2009_SMS | pLARmEB | Vrs1 | 2H: 652030802 | -17.74 | 30.51 | 68.71 |
| 2009_SMS | ISIS EM-BLASSO | Vrs1 | 2H: 652030802 | -19.71 | 59.21 | 84.53 |
| 2010_SMS | mrMLM | Vrs1 | 2H: 652030802 | -18.81 | 15.94 | 66.62 |
| 2010_SMS | FASTmrMLM | Vrs1 | 2H: 652030802 | -19.53 | 35.93 | 71.75 |
| 2010_SMS | FASTmrEMMA | Vrs1 | 2H: 652030802 | -43.84 | 35.22 | 90.41 |
| 2010_SMS | pLARmEB | Vrs1 | 2H: 652030802 | -21.20 | 57.22 | 71.96 |
| 2010_SMS | ISIS EM-BLASSO | Vrs1 | 2H: 652030802 | -21.69 | 56.11 | 88.54 |
| 2012_SMS | mrMLM | Vrs1 | 2H: 652030802 | -21.82 | 19.99 | 86.00 |
| 2012_SMS | FASTmrMLM | Vrs1 | 2H: 652030802 | -21.68 | 32.99 | 86.32 |
| 2012_SMS | FASTmrEMMA | Vrs1 | 2H: 652030802 | -35.83 | 22.00 | 65.07 |
| 2012_SMS | pLARmEB | Vrs1 | 2H: 652030802 | -21.32 | 68.02 | 76.19 |
| 2012_SMS | ISIS EM-BLASSO | Vrs1 | 2H: 652030802 | -21.91 | 64.75 | 86.43 |
| BLUP_SMS | FASTmrEMMA | vrs1 | 2H: 652030802 | -36.56 | 29.32 | 74.96 |
| BLUP_SMS | FASTmrMLM | vrs1 | 2H: 652030802 | -18.15 | 29.32 | 73.88 |
| BLUP_SMS | ISIS EM-BLASSO | vrs1 | 2H: 652030802 | -21.61 | 82.16 | 90.03 |
| BLUP_SMS | mrMLM | vrs1 | 2H: 652030802 | -19.10 | 13.63 | 81.82 |
| BLUP_SMS | pLARmEB | vrs1 | 2H: 652030802 | -18.59 | 48.68 | 72.30 |
| 2009_SLP | mrMLM | 2_524762464 | 2H: 649558019 | -112.54 | 21.01 | 42.66 |
| 2009_SLP | FASTmrMLM | 2_524762464 | 2H: 649558019 | -63.75 | 4.51 | 15.21 |
| 2009_SLP | FASTmrEMMA | 2_524762464 | 2H: 649558019 | -212.39 | 19.01 | 40.31 |
| 2009_SLP | ISIS EM-BLASSO | 2_524762464 | 2H: 649558019 | -122.24 | 25.72 | 55.05 |
| 2010_SLP | pLARmEB | 2_524762464 | 2H: 649558019 | -60.80 | 4.74 | 14.87 |
| 2012_SLP | mrMLM | Vrs1 | 2H: 652030802 | -211.36 | 3.32 | 29.16 |
| 2012_SLP | FASTmrMLM | Vrs1 | 2H: 652030802 | -286.97 | 17.19 | 68.19 |
| 2012_SLP | FASTmrEMMA | Vrs1 | 2H: 652030802 | -605.54 | 17.32 | 72.21 |
| 2012_SLP | pLARmEB | Vrs1 | 2H: 652030802 | -233.18 | 35.66 | 57.20 |
| 2012_SLP | ISIS EM-BLASSO | Vrs1 | 2H: 652030802 | -302.28 | 18.50 | 71.33 |
| BLUP_SLP | FASTmrEMMA | vrs1 | 2H: 652030802 | -245.26 | 38.81 | 68.78 |
| BLUP_SLP | FASTmrMLM | vrs1 | 2H: 652030802 | -119.45 | 41.30 | 65.26 |
| BLUP_SLP | ISIS EM-BLASSO | vrs1 | 2H: 652030802 | -131.34 | 51.41 | 77.51 |
| BLUP_SLP | mrMLM | vrs1 | 2H: 652030802 | -152.87 | 4.66 | 80.30 |
| BLUP_SLP | pLARmEB | vrs1 | 2H: 652030802 | -119.45 | 27.21 | 59.40 |
| 2009_SLP | FASTmrMLM | 2HL_17075593 | 2H: 653982961 | -48.96 | 3.90 | 8.97 |
| 2009_SLP | pLARmEB | 2HL_17075593 | 2H: 653982961 | -56.87 | 3.87 | 11.44 |
| 2010_SLP | pLARmEB | 2HL_17075593 | 2H: 653982961 | -68.55 | 5.90 | 18.90 |
| 2010_SLP | ISIS EM-BLASSO | 2HL_17075593 | 2H: 653982961 | -58.99 | 3.82 | 14.00 |
| 2009_SLP | FASTmrEMMA | 4_16553551 | 4H: 15498372 | -54.80 | 3.26 | 3.09 |
| 2009_SLP | ISIS EM-BLASSO | 4_16553551 | 4H: 15498372 | -30.19 | 4.50 | 3.92 |
| BLUP_SLP | FASTmrMLM | 4_16553551 | 4H: 15498372 | -24.57 | 5.29 | 3.22 |
| 2010_SLP | ISIS EM-BLASSO | M_1605646_794 | 4H: 16761959 | -24.30 | 4.35 | 2.82 |
| BLUP_SLP | ISIS EM-BLASSO | M_1605646_794 | 4H: 16761959 | -27.85 | 8.06 | 4.14 |
| BLUP_SLP | mrMLM | M_1605646_794 | 4H: 16761959 | -28.24 | 6.81 | 3.26 |
| BLUP_SLP | pLARmEB | M_1605646_794 | 4H: 16761959 | -28.40 | 9.76 | 3.99 |
| 2012_SP | pLARmEB | 2_522610509 | 2H: 648821931 | 0.85 | 4.02 | 7.61 |
| BLUP_SP | FASTmrEMMA | 2_522610509 | 2H: 648821931 | 1.14 | 16.22 | 31.98 |
| 2009_SP | FASTmrEMMA | 2_522600068 | 2H: 649657420 | 1.81 | 6.58 | 12.75 |
| BLUP_SP | FASTmrMLM | 2_522600068 | 2H: 649657420 | 0.60 | 17.52 | 36.37 |
| BLUP_SP | ISIS EM-BLASSO | 2_522600068 | 2H: 649657420 | 0.60 | 16.53 | 36.31 |
| BLUP_SP | mrMLM | 2_522600068 | 2H: 649657420 | 0.63 | 16.09 | 39.25 |
| 2010_SP | mrMLM | Vrs1 | 2H: 652030802 | 1.41 | 23.32 | 31.20 |
| 2010_SP | FASTmrMLM | Vrs1 | 2H: 652030802 | 1.39 | 24.31 | 36.68 |
| 2010_SP | FASTmrEMMA | Vrs1 | 2H: 652030802 | 2.77 | 10.71 | 47.33 |
| 2010_SP | pLARmEB | Vrs1 | 2H: 652030802 | 1.64 | 30.41 | 39.81 |
| 2010_SP | ISIS EM-BLASSO | Vrs1 | 2H: 652030802 | 1.49 | 22.14 | 52.32 |
| 2012_SP | mrMLM | 2_531255437 | 2H: 662335248 | 1.02 | 3.83 | 10.91 |
| 2012_SP | FASTmrMLM | 2_531255437 | 2H: 662335248 | 0.81 | 4.68 | 6.88 |
| 2012_SP | FASTmrEMMA | 2_531255437 | 2H: 662335248 | 1.76 | 4.80 | 7.51 |
| 2012_SP | ISIS EM-BLASSO | 2_531255437 | 2H: 662335248 | 0.79 | 3.96 | 6.49 |
| 2009_SP | mrMLM | M_124056_833 | 2H: 663628734 | 0.94 | 7.32 | 11.95 |
| 2009_SP | FASTmrMLM | M_124056_833 | 2H: 663628734 | 0.76 | 3.73 | 10.21 |
| 2010_GP | FASTmrMLM | 2_524762464 | 2H: 649558019 | -59.22 | 6.23 | 28.89 |
| 2010_GP | FASTmrEMMA | 2_524762464 | 2H: 649558019 | -118.41 | 6.23 | 27.59 |
| 2010_GP | pLARmEB | 2_524762464 | 2H: 649558019 | -60.18 | 7.54 | 29.84 |
| 2010_GP | ISIS EM-BLASSO | 2_524762464 | 2H: 649558019 | -49.45 | 3.46 | 20.15 |
| BLUP_GP | ISIS EM-BLASSO | M_1589358_1352 | 2H: 650438830 | -39.38 | 17.05 | 35.35 |
| BLUP_GP | mrMLM | M_1589358_1352 | 2H: 650438830 | -40.53 | 4.82 | 37.44 |
| BLUP_GP | pLARmEB | M_1589358_1352 | 2H: 650438830 | -29.30 | 5.43 | 19.56 |
| 2012_GP | mrMLM | Vrs1 | 2H: 652030802 | -119.91 | 7.31 | 21.00 |
| 2012_GP | FASTmrMLM | Vrs1 | 2H: 652030802 | -109.74 | 7.31 | 17.59 |
| 2012_GP | FASTmrEMMA | Vrs1 | 2H: 652030802 | -219.41 | 7.31 | 17.58 |
| BLUP_GP | FASTmrMLM | vrs1 | 2H: 652030802 | -38.61 | 16.71 | 34.59 |
| 2009_GP | FASTmrEMMA | 2_527241334 | 2H: 652604015 | -32.75 | 3.42 | 3.41 |
| 2009_GP | ISIS EM-BLASSO | 2_527241334 | 2H: 652604015 | -16.38 | 3.84 | 3.61 |
| 2009_GP | mrMLM | 4_497278091 | 4H: 596447744 | 23.46 | 4.76 | 7.77 |
| 2009_GP | FASTmrMLM | 4_497278091 | 4H: 596447744 | 17.18 | 3.59 | 4.41 |
| 2009_GP | FASTmrEMMA | 4_497278091 | 4H: 596447744 | 37.72 | 4.25 | 4.70 |
| 2009_GP | pLARmEB | 4_497278091 | 4H: 596447744 | 22.12 | 5.02 | 7.32 |
| 2009_GP | ISIS EM-BLASSO | 4_497278091 | 4H: 596447744 | 19.26 | 4.98 | 5.55 |
| 2012_GS | pLARmEB | 2_524762464 | 2H: 649558019 | -5.51 | 3.04 | 16.51 |
| BLUP_GS | mrMLM | 2_524762464 | 2H: 649558019 | -8.27 | 3.07 | 71.50 |
| BLUP_GS | pLARmEB | 2_524762464 | 2H: 649558019 | -3.06 | 5.00 | 9.77 |
| 2009_GS | mrMLM | 2_527636020 | 2H: 651399477 | -4.51 | 17.44 | 26.71 |
| 2009_GS | FASTmrMLM | 2_527636020 | 2H: 651399477 | -4.75 | 17.50 | 29.64 |
| 2009_GS | FASTmrEMMA | 2_527636020 | 2H: 651399477 | -7.03 | 4.85 | 15.72 |
| 2010_GS | FASTmrMLM | Vrs1 | 2H: 652030802 | -13.12 | 25.77 | 65.25 |
| 2010_GS | FASTmrEMMA | Vrs1 | 2H: 652030802 | -25.77 | 23.77 | 62.96 |
| 2010_GS | pLARmEB | Vrs1 | 2H: 652030802 | -13.19 | 11.28 | 65.93 |
| 2010_GS | ISIS EM-BLASSO | Vrs1 | 2H: 652030802 | -12.32 | 27.85 | 57.58 |
| 2012_GS | FASTmrMLM | Vrs1 | 2H: 652030802 | -9.96 | 25.21 | 53.95 |
| 2012_GS | ISIS EM-BLASSO | Vrs1 | 2H: 652030802 | -9.59 | 26.98 | 49.99 |
| BLUP_GS | FASTmrMLM | vrs1 | 2H: 652030802 | -8.31 | 22.14 | 72.25 |
| BLUP_GS | ISIS EM-BLASSO | vrs1 | 2H: 652030802 | -7.90 | 48.96 | 65.23 |
| 2009_GS | mrMLM | 2_625783669 | 2H: 764361924 | -2.17 | 3.48 | 7.80 |
| 2009_GS | FASTmrEMMA | 2_625783669 | 2H: 764361924 | -3.70 | 3.97 | 4.86 |
| 2009_GS | ISIS EM-BLASSO | 2_625783669 | 2H: 764361924 | -2.46 | 5.87 | 10.05 |
| 2010_GS | pLARmEB | 2_625783669 | 2H: 764361924 | -1.53 | 4.21 | 1.06 |
| 2010_GS | ISIS EM-BLASSO | 2_625783669 | 2H: 764361924 | -1.63 | 4.83 | 1.19 |
| 2009_GWP | mrMLM | 4_497278091 | 4H: 596447744 | 0.66 | 6.81 | 9.17 |
| 2009_GWP | FASTmrMLM | 4_497278091 | 4H: 596447744 | 0.54 | 5.49 | 6.18 |
| 2009_GWP | FASTmrEMMA | 4_497278091 | 4H: 596447744 | 0.95 | 4.03 | 4.26 |
| 2009_GWP | pLARmEB | 4_497278091 | 4H: 596447744 | 0.43 | 4.36 | 3.97 |
| 2009_GWS | FASTmrEMMA | 2_625783669 | 2H: 764361924 | -0.07 | 3.29 | 3.41 |
| 2009_GWS | pLARmEB | 2_625783669 | 2H: 764361924 | -0.04 | 5.28 | 4.95 |
| 2009_GWS | ISIS EM-BLASSO | 2_625783669 | 2H: 764361924 | -0.05 | 7.37 | 6.28 |
| 2010_GWS | pLARmEB | 2_625783669 | 2H: 764361924 | -0.04 | 4.42 | 0.74 |
| 2009_GWS | mrMLM | 4_497278091 | 4H: 596447744 | 0.05 | 5.14 | 8.83 |
| 2009_GWS | FASTmrMLM | 4_497278091 | 4H: 596447744 | 0.05 | 3.04 | 7.31 |
| 2009_GWS | ISIS EM-BLASSO | 4_497278091 | 4H: 596447744 | 0.04 | 3.61 | 5.59 |
| 2009_TGW | mrMLM | Vrs1 | 2H: 652030802 | 5.04 | 26.47 | 46.16 |
| 2009_TGW | FASTmrMLM | Vrs1 | 2H: 652030802 | 5.00 | 4.82 | 46.09 |
| 2009_TGW | FASTmrEMMA | Vrs1 | 2H: 652030802 | 9.87 | 23.84 | 44.89 |
| 2009_TGW | pLARmEB | Vrs1 | 2H: 652030802 | 4.65 | 27.80 | 39.86 |
| 2010_TGW | FASTmrMLM | Vrs1 | 2H: 652030802 | 7.48 | 43.57 | 54.79 |
| 2010_TGW | FASTmrEMMA | Vrs1 | 2H: 652030802 | 15.22 | 7.37 | 56.66 |
| 2010_TGW | pLARmEB | Vrs1 | 2H: 652030802 | 5.79 | 16.10 | 32.77 |
| 2010_TGW | ISIS EM-BLASSO | Vrs1 | 2H: 652030802 | 7.57 | 49.42 | 56.12 |
| BLUP_TGW | FASTmrEMMA | vrs1 | 2H: 652030802 | 12.32 | 7.39 | 55.32 |
| BLUP_TGW | FASTmrMLM | vrs1 | 2H: 652030802 | 5.96 | 42.66 | 51.76 |
| BLUP_TGW | ISIS EM-BLASSO | vrs1 | 2H: 652030802 | 6.01 | 36.78 | 52.55 |
| BLUP_TGW | mrMLM | vrs1 | 2H: 652030802 | 5.84 | 11.89 | 49.60 |
| BLUP_TGW | pLARmEB | vrs1 | 2H: 652030802 | 5.84 | 46.32 | 49.72 |
| 2010_TGW | mrMLM | 3HS_23539468 | 3H: 272283784 | -1.89 | 3.89 | 3.78 |
| 2010_TGW | FASTmrMLM | 3HS_23539468 | 3H: 272283784 | -1.55 | 4.84 | 2.52 |
| BLUP_TGW | FASTmrMLM | 3HS_23539468 | 3H: 272283784 | -1.31 | 5.83 | 2.68 |
| BLUP_TGW | mrMLM | 3HS_23539468 | 3H: 272283784 | -1.67 | 5.98 | 4.39 |
| BLUP_TGW | pLARmEB | 3HS_23539468 | 3H: 272283784 | -1.18 | 5.97 | 2.19 |
| 2010_TGW | mrMLM | 7HS_10887541 | 7H: 72344563 | 3.06 | 6.94 | 11.22 |
| 2010_TGW | FASTmrMLM | 7HS_10887541 | 7H: 72344563 | 2.44 | 8.79 | 7.11 |
| 2010_TGW | FASTmrEMMA | 7HS_10887541 | 7H: 72344563 | 5.05 | 8.93 | 7.15 |
| 2010_TGW | pLARmEB | 7HS_10887541 | 7H: 72344563 | 2.31 | 13.16 | 6.37 |
| 2010_TGW | ISIS EM-BLASSO | 7HS_10887541 | 7H: 72344563 | 2.37 | 10.46 | 6.70 |
| BLUP_TGW | FASTmrMLM | 7HS_10887541 | 7H: 72344563 | 1.57 | 6.27 | 4.39 |
| BLUP_TGW | mrMLM | 7HS_10887541 | 7H: 72344563 | 1.95 | 7.25 | 6.77 |

^a^ Trait ID was defined as “year+ abbreviation trait name
